# Regulatory cross-talk supports resistance to Zn intoxication in *Streptococcus*

**DOI:** 10.1371/journal.ppat.1010607

**Published:** 2022-07-21

**Authors:** Matthew J. Sullivan, Kelvin G. K. Goh, Glen C. Ulett

**Affiliations:** School of Pharmacy and Medical Sciences, and Menzies Health Institute Queensland, Griffith University, Gold Coast, Australia; Boston Children’s Hospital, UNITED STATES

## Abstract

Metals such as copper (Cu) and zinc (Zn) are important trace elements that can affect bacterial cell physiology but can also intoxicate bacteria at high concentrations. Discrete genetic systems for management of Cu and Zn efflux have been described in several bacterial pathogens, including streptococci. However, insight into molecular cross-talk between systems for Cu and Zn management in bacteria that drive metal detoxification, is limited. Here, we describe a biologically consequential cross-system effect of metal management in group B *Streptococcus* (GBS) governed by the Cu-responsive *copY* regulator in response to Zn. RNAseq analysis of wild-type (WT) and *copY*-deficient GBS subjected to metal stress revealed unique transcriptional links between the systems for Cu and Zn detoxification. We show that the Cu-sensing role of CopY extends beyond Cu and enables CopY to regulate Cu and Zn stress responses that effect changes in gene function for central cellular processes, including riboflavin synthesis. CopY also supported GBS intracellular survival in human macrophages and virulence during disseminated infection in mice. In addition, we show a novel role for CovR in modulating GBS resistance to Zn intoxication. Identification of the Zn resistome of GBS using TraDIS revealed a suite of genes essential for GBS growth in metal stress. Several of the genes identified are novel to systems that support bacterial survival in metal stress and represent a diverse set of mechanisms that underpin microbial metal homeostasis during cell stress. Overall, this study reveals a new and important mechanism of cross-system complexity driven by CopY in bacteria to regulate cellular management of metal stress and survival.

## Author summary

Metals, such as Cu and Zn, can be used by the mammalian immune system to target bacterial pathogens for destruction, and consequently, bacteria have evolved discrete genetic systems to enable subversion of this host antimicrobial response. Systems for Cu and Zn homeostasis are well characterized, including transcriptional control elements that sense and respond to metal stress. Here, we discover novel features of metal response systems in *Streptococcus*, which have broad implications for bacterial pathogenesis and virulence. We show that *Streptococcus* resists Zn intoxication by utilizing a *bona fide* Cu regulator, CopY, to manage cellular metal homeostasis, and enable the bacteria to survive stressful conditions. We identify several new genes that confer resistance to Zn intoxication in *Streptococcus*, including some that have hitherto not been linked to metal ion homeostasis in any bacterial pathogen. Identification of a novel cross-system metal management mechanism exploited by *Streptococcus* to co-ordinate and achieve metal resistance enhances our understanding of metal ion homeostasis in bacteria and its effect on pathogenesis.

## Introduction

In prokaryotic and eukaryotic cells, copper (Cu) and zinc (Zn) are important cofactors for metalloenzymes [1,2]. When present in excess, however, Cu and Zn can cause cellular toxicity and, for example, can exert antimicrobial effects in subcellular areas within infected phagocytes [3,4]. The double-edged sword of Cu and Zn for supporting cellular physiology versus toxicity offers potential antimicrobial benefit for the control of bacterial pathogens and is of interest for the study of host-pathogen interactions [5–7]. On the one hand, Cu intoxication in bacteria can reflect enzyme inactivation, deregulation of metabolism, and/or redox stress, such as higher potential to generate reactive oxygen species [8]. Zn intoxication can reflect an ablation of uptake of essential manganese (Mn) [9], which can compromise the bacterial cellular response to oxidative stress [10]; excess Zn can also disrupt central carbon metabolism [11]. Phagocytes such as macrophages and neutrophils can mobilise intracellular pools of Cu and Zn to expose internalized bacteria to metal conditions that are antimicrobial [5,12,13]. In some pathogenic bacteria, this can be counteracted by activation of metal efflux mechanisms to thwart metal intoxication [14].

In bacteria, adaptation to metal excess and limitation is complex, but several defined systems are based on efflux proteins including P-type ATPases, which confer resistance to metal stress in different pathogens [1,3]. In streptococci, discrete genetic systems for cellular management of Cu and Zn homeostasis act via the regulation of metal import and export machinery [9,15,16]; a system for Cu efflux utilizes the canonical *cop* operon, encompassing *copA* that encodes a ATPase efflux pump that extrudes cellular Cu ions, alongside a Cu-specific transcriptional regulator *copY*, that represses the operon [17,18]. A system for Zn efflux uses a Zn-specific transcriptional response regulator, *sczA*, to control a Zn efflux transporter, encoded by *czcD* [15,19]. These two systems of *copA-copY* and *czcD*-*sczA* for Cu and Zn export, respectively, have recently been characterized in group B *Streptococcus* (GBS), which responds to excess Cu and Zn by de-repressing *copA* via CopY to drive Cu export from the cell [20], and by activating *czcD* via SczA to regulate cellular Zn levels [21], respectively.

Molecular cross-talk between Cu and Zn management systems in bacteria can be proposed by several observations reported in prior studies of different pathogens. Zn was shown to bind CopY in *Enterococcus* [22], and was linked with a disruption in cellular Cu content in *Acinetobacter* [14]. In *Streptococcus pneumoniae*, Zn inhibits the expression of *copY*, which implies that Zn may act as a non-cognate co-repressor of *copY* [17]. In *Pseudomonas stutzeri*, overlapping regulation of genes that mediate Cu and Zn resistance has recently been reported [23], suggesting that a core set of bacterial genes respond to Cu and Zn, and are co-regulated. Finally, a Cu-responsive regulatory system for Cu uptake in *Candida* was recently reported to respond to Iron (Fe)-starvation [24]. Collectively, these observations point to complexity within bacterial adaptation stress responses to metal excess and limitation, and potential cross-talk mechanisms between metal management systems that might support cellular homeostasis and bacterial fitness in distinct environmental conditions. A cross-talk mechanism of Zn-mediated signalling effects through *copY* as a means of bacterial adaptation to metal stress has not been defined.

The control of cellular activities involved in pathogenesis due to beta-hemolytic streptococci is complex, and encompasses multi-level systems to engage numerous regulators that sense a variety of environmental or cellular cues [25]. A prominent two-component system that functions as a central axis for the regulation of virulence in streptococci is CovRS / CsrRS, which controls the expression of pathogenicity factors such as capsule [26], pili [27] adhesins [28] and cytotoxins modulates up to 10% of the GBS genome [29]. CovRS promotes blood-brain barrier penetration, meningitis, neonatal infection, genital tract colonisation and urinary tract infections [29–31]. Some studies have reported that CovR regulates genes for metal ion homeostasis, including genes for Zn acquisition and efflux [26,29]; however, the potential role of CovR for affording metal ion resistance to pathogenic streptococci has not been explored.

Here, we examined Zn management in GBS, as a prominent pathogenic *Streptococcus* with clearly defined metal detoxification systems in *copA-copY* and *czcD*-*sczA*, to determine whether CopY functions as a cross-system regulator of the bacteria’s response to Zn stress. We explored links between the defined systems for Cu and Zn detoxification in GBS on a transcriptome and global genomic scale to reveal the genome that supports Zn resistance, and effects on macrophage survival and virulence.

## Results

### Identification of CopY-mediated cross-over control of multiple metal efflux pathways

**Fig 1:**
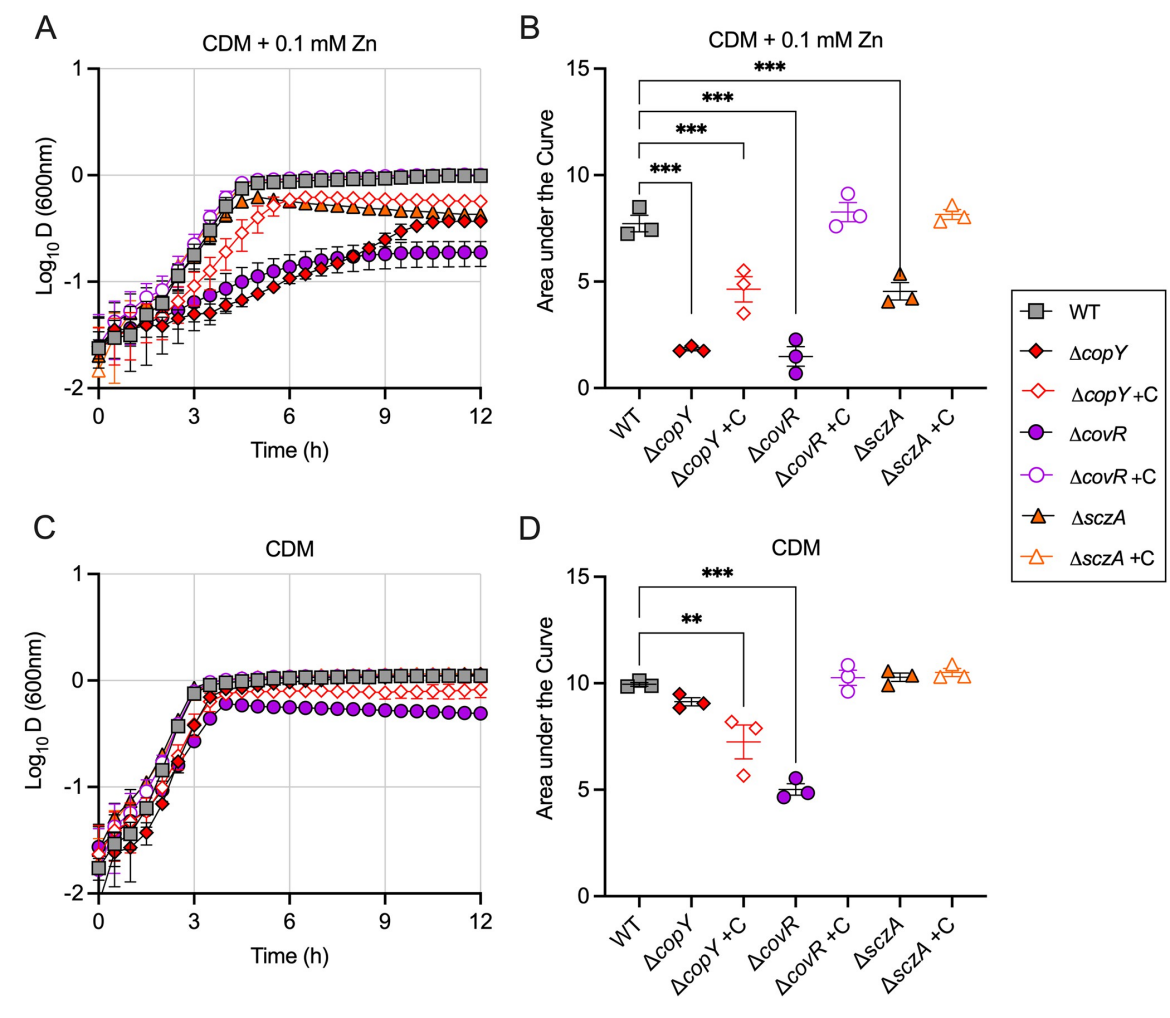
Growth of GBS in CDM using conditions of Zn stress. WT, Δ*copY*, Δ*covR* and Δ*sczA* mutants and corresponding complemented strains (indicated by +C) were grown in CDM medium supplemented with 0.1 mM Zn (A) and compared (B) using Area Under the Curve analysis followed by ordinary one-way ANOVA and Holm Sidak multiple comparisons (** P < 0.01, *** P < 0.005). Growth curves of the bacteria in control conditions (CDM medium alone) are shown for comparison (C) and compared (D) using Area Under the Curve analysis in followed by ordinary one-way ANOVA and Holm Sidak multiple comparisons (** P < 0.01, *** P < 0.005). Measures of Attenuance (*D* at 600nm) or Area Under the Curve lines shown are mean ± S.E.M (*n* = 3 biological repeats).

We recently defined that CopY regulates GBS responses to Cu stress transcriptionally via control of *copA* [20], and that *sczA* regulates responses to Zn stress via *czcD* [21]. Here, we examined the existence of cross-control of metal efflux pathways by non-cognate regulators by comparing the growth and metal stress resistance phenotypes of GBS mutants deficient in *copY* or *sczA* using defined *in vitro* conditions of either Cu stress (for Δ*sczA*) or Zn stress (for Δ*copY*). This revealed unexpected and significant cross-system regulatory effects of CopY on the capacity of GBS to resist Zn intoxication. GBS was rendered severely susceptible to Zn intoxication as a result of *copY* mutation, according to growth analysis in a chemically-defined minimal medium (CDM) supplemented with 0.1 mM Zn (Fig 1). Similarly, *copY* supported GBS resistance to Zn stress in nutritionally-rich THB medium (S1 Fig). Complementation of Δ*copY in trans* restored growth of the mutant in Zn stress, and restored repression of *copA* expression (S2 Fig). In contrast, we detected no significant perturbation of the growth of Δ*sczA* GBS in Cu stress, indicating no role for SczA in mediating Cu resistance (1.5 mM in THB or 1 mM in CDM; S3 Fig). Together, these findings establish that *copY* regulates the ability of GBS to respond to and resist Zn intoxication.

Several pathogenic *Streptococcus* spp. respond to environmental stress cues, including excess metal ions (*e*.*g*., Mg^2+^) via global response regulators, including *covRS* [28,32,33]. In GBS, a few genes that support Zn homeostasis have been linked to *covR*-regulation, with *adcR-adcCB* for Zn import (GBS 2603V/R), *adcAII-shtII* (GBS BM110 [26]), and *czcD* for Zn efflux (GBS 2603V/R and NEM316 [34,35]). In considering these studies, we examined the cross-system effect of Zn and Cu stress at the point of *covR* by analyzing growth of Δ*covR* GBS in Zn or Cu stress. This showed significantly enhanced susceptibility of *covR* GBS to Zn stress, identifying a major role for *covR* in supporting GBS resistance to Zn intoxication (Figs 1 and S1). The mutant was also rendered more susceptible to Cu intoxication but to a lesser extent (S3 Fig). Thus, *covR* facilitates metal resistance in GBS in a manner similar to the dual-metal resistance function of CopY (for Zn and Cu intoxication).

### CopY facilitates the management of multiple cell-associated metal pools during Zn stress

**Fig 2:**
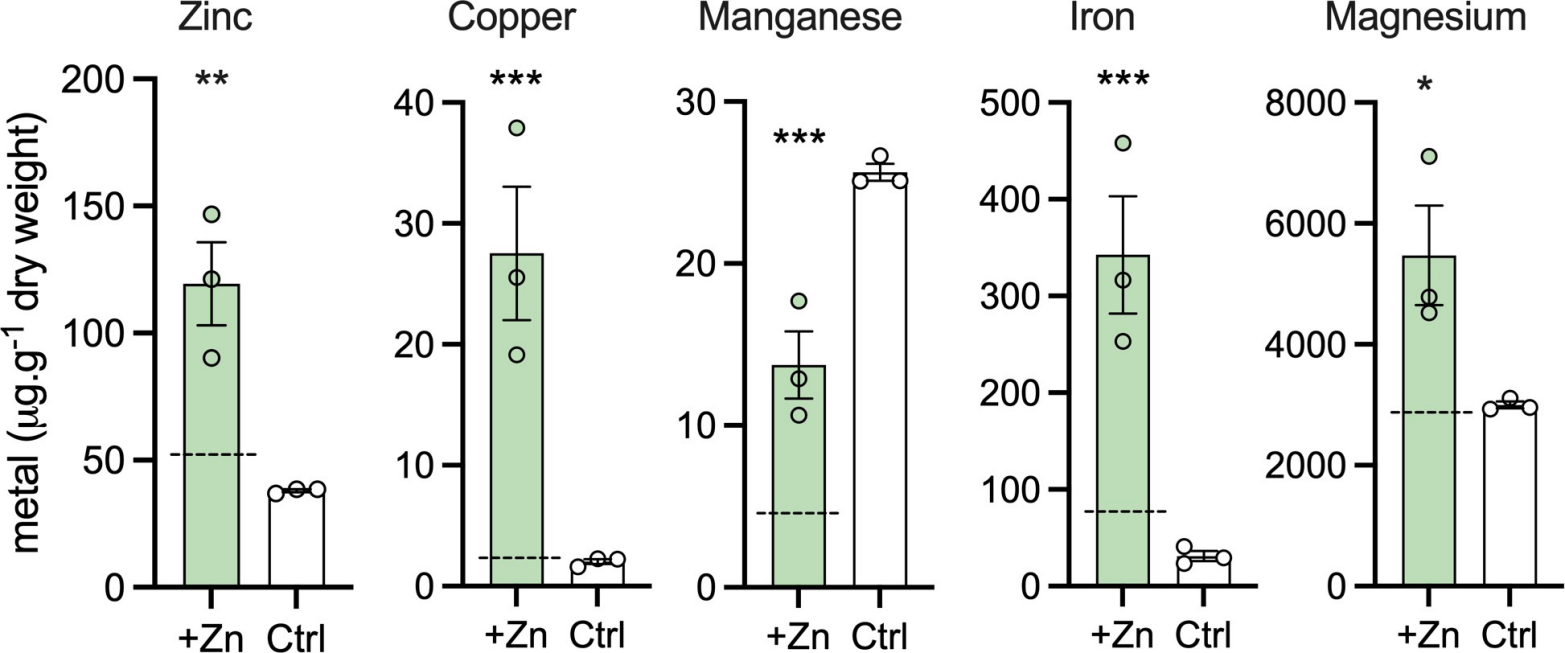
Accumulation of cell-associated Zn, Cu, Mn, Fe or Mg in Δ*copY* GBS with or without Zn stress. Δ*copY* GBS was exposed to Zn (0.25 mM; green bars) and cellular metal content was compared to non-exposed controls (THB only; white bars) using Inductively coupled plasma optical emission spectrometry (ICP-OES). Metal content is shown, with values normalised using dry weight of bacterial cell pellets (μg metal per g dry weight biomass). Bars show mean ± S.E.M (*n* = 3 biological repeats). Values for each metal for Δ*copY* during Zn stress (+Zn) were compared to WT grown in identical conditions, by ordinary one-way ANOVA and Holm-Sidak multiple comparisons (*P < 0.05, ** P < 0.01, ***P < 0.001). Mean values of WT GBS in Zn stress (0.25 mM) are shown with dotted lines; values for Zn, Cu, Mn and Fe are published elsewhere [21].

We used Inductively coupled plasma optical emission spectrometry (ICP-OES) to measure the total cellular content of metals in WT and Δ*copY* GBS that were exposed to Zn stress, which showed that mutation of *copY* caused mis-management of the cellular metal pools of multiple metals, including Zn, Cu, Mn, Fe and Mg. Δ*copY* GBS exhibited significantly elevated levels of cellular Zn, Cu, Mn, Fe and Mg compared to WT in identical conditions (Fig 2). This indicated that broad mis-management of cellular metal homeostasis in Δ*copY* GBS occurs primarily as a consequence of Zn stress; in a prior study of Cu stress in this strain, we reported modest but significant increase in cellular Zn content [20], but no change in Mn or Fe. Thus, the effects of *copY* on metal homeostasis extend beyond Cu export to facilitate the regulation of cell-associated pools of multiple metals in GBS exposed to non-cognate metal stress. Mutation in *covR* reduced cellular Mn content in THB conditions in the absence of Zn stress but no statistically significant differences were observed in comparisons to WT in identical conditions (S4 Fig).

### Role of CopY in macrophage killing of GBS

**Fig 3:**
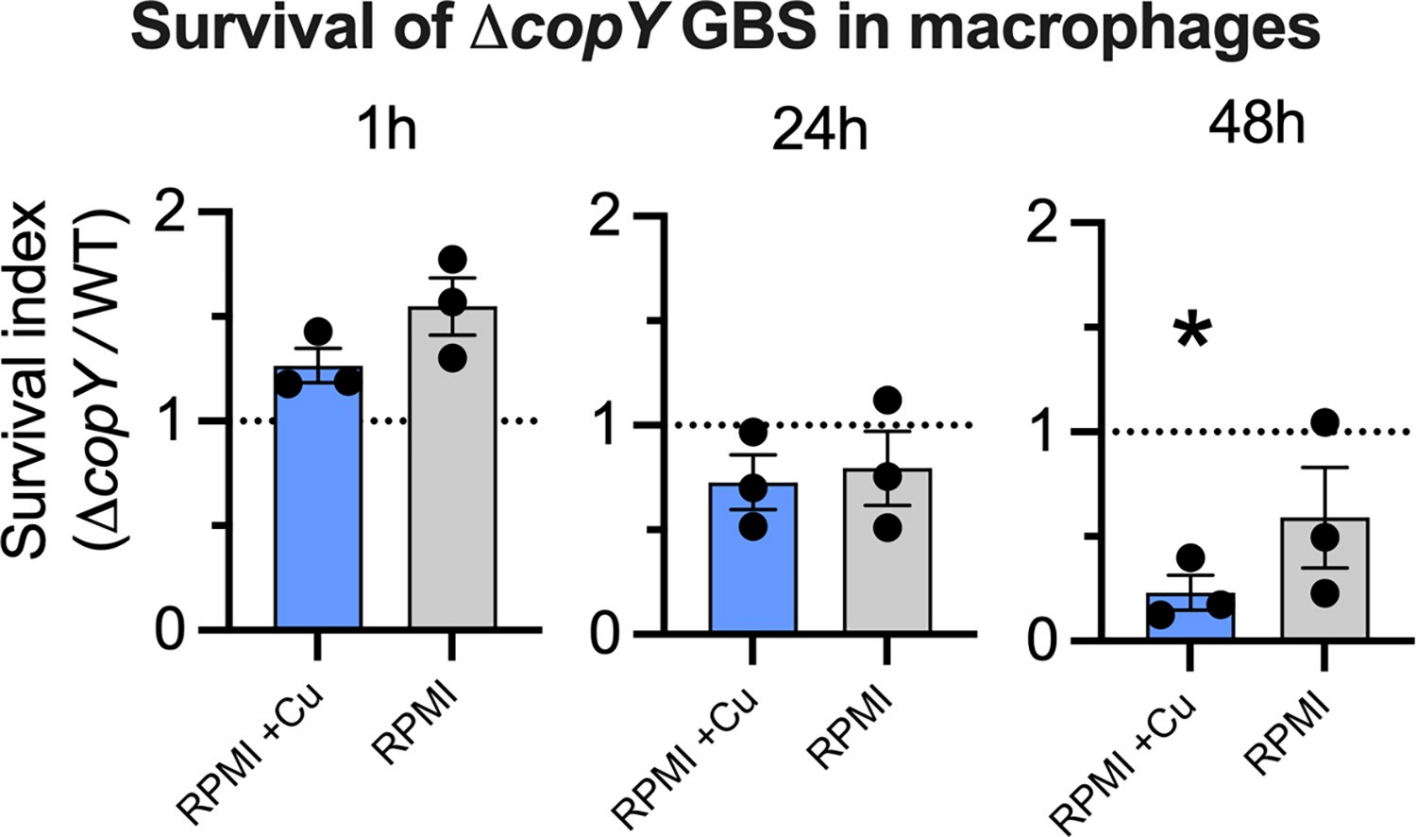
The role of *copY* in macrophage killing of GBS. Gentamicin protection assays with WT and Δ*copY* strains in human U937 MDMs in RPMI culture medium supplemented with 20 μM Cu (Cu-supp; blue bars) and standard RPMI medium (Standard; grey bars). Intracellular survival of Δ*copY* GBS is shown as a ratio of CFU per milliliter of Δ*copY* GBS divided by CFU per milliliter of WT GBS (Survival Index). Dotted lines at index = 1 show no difference to WT. Bars show mean ± S.E.M (*n* = 3 biological repeats), assays were analysed by One sample *t* tests against a survival index value of 1 (*P < 0.05).

To determine if *copY* supports survival of GBS inside phagocytic cells, we used human monocyte-derived macrophage-like cells (MDMs) in antibiotic protection assays. Monolayers of MDMs were infected with WT and Δ*copY* GBS for 1h and antibiotics (penicillin, streptomycin and gentamicin) were added to kill extracellular bacteria. Viable intracellular GBS were quantified at 1h post-antibiotic addition and at 24h and 48h to assess survival of the bacteria inside phagocytes. The survival index of the Δ*copY* strain was calculated as CFU/mL of Δ*copY* GBS divided by CFU/mL of WT (Index of 1 = no difference). We ran parallel assays comparing culture conditions of standard RPMI medium to RPMI medium supplemented with 20 μM Cu to ensure adequate availability for Cu-loading of MDMs. There was no difference in survival of Δ*copY* GBS compared to WT at 1h or 24h, regardless of the addition of Cu to the MDMs. At 48h, significantly fewer intracellular Δ*copY* GBS were recovered compared to WT in Cu-loaded MDMs (Fig 3), indicating that, after 48h, *copY* contributes to survival inside MDMs in a manner that is dependent on host-metal status. Absolute bacterial counts are shown in S5 Fig).

### The transcriptional foundation of CopY cross-system control of Zn homeostasis in GBS

**Fig 4:**
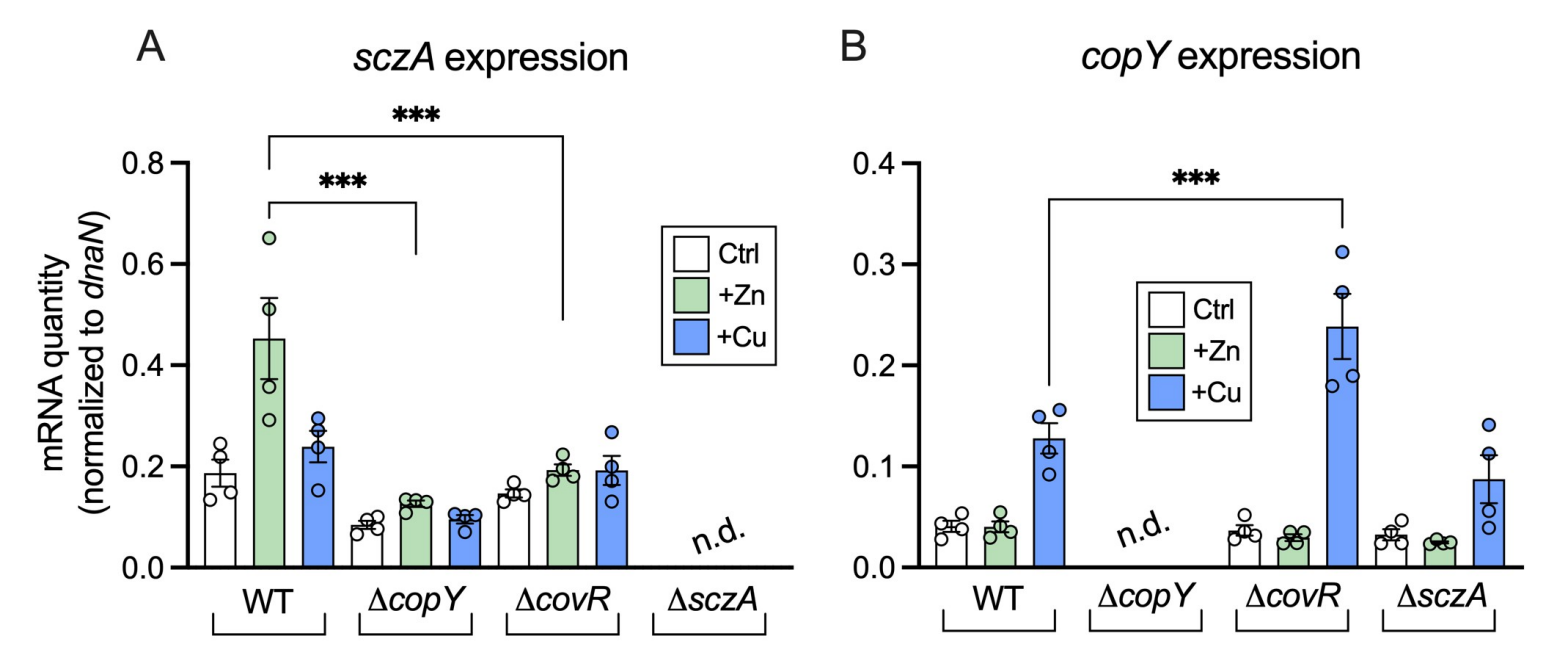
Cross-regulation of Zn and Cu stress responses by *copY* and *covR*. Transcripts of *sczA* (A) and *copY* (B) were quantified by qRTPCR from cultures of WT, Δ*copY*, Δ*covR and* Δ*sczA* mutants supplemented with Zn (0.25 mM) or Cu (0.5 mM) and compared to non-exposed (THB only) controls (*n* = 4). Absolute transcript amounts were normalized using housekeeping *dnaN* and generated from standard curves using GBS genomic DNA. Transcripts of *sczA* and *copY* were not detected (n.d.) in the Δ*sczA* and Δ*copY* mutant strains, respectively. Quantities were compared using ordinary one-way ANOVA and Holm Sidak multiple comparisons (***P < 0.001).

To discern the effect of *copY* mutation on bacterial transcriptional responses to Zn stress, we analyzed the expression of genes that contribute to Zn resistance, namely *czcD* and *sczA* using qRTPCR to compare Δ*copY* and WT GBS. Additionally, we analyzed Cu-responsive genes (*copA* and *copY*) in Zn stress, in Cu stress and in non-exposed controls. Unexpectedly, we detected a significant cross-system effect whereby *copY* was essential for the induction of *sczA* by Zn (Fig 4A). Transcriptional activation of *sczA* in response to Zn stress did not occur in the Δ*covR* strain (Fig 4A). Interestingly, we found that *copY* expression was controlled, in part, by *covR* in the response to Cu stress, but not Zn stress (Fig 4B); whereby Cu stress increased the transcription of *copY* in Δ*covR* GBS (compared to WT in Cu stress). Notably, modulation of i) *sczA* by CopY (or CovR) did not affect *czcD* expression, nor did modulation of *copY* by CovR affect *copA* expression (S6 Fig) suggesting that i) sensitivity of Δ*copY* GBS to Zn stress is not due to differences in Zn-efflux (*czcD* expression), and ii) *covR* acts as an auxiliary regulator to govern the expression of master regulatory genes (e.g., *copY)* that control Cu and Zn efflux.

### Defining the *copY*-regulated transcriptome of GBS exposed to Zn stress

**Fig 5:**
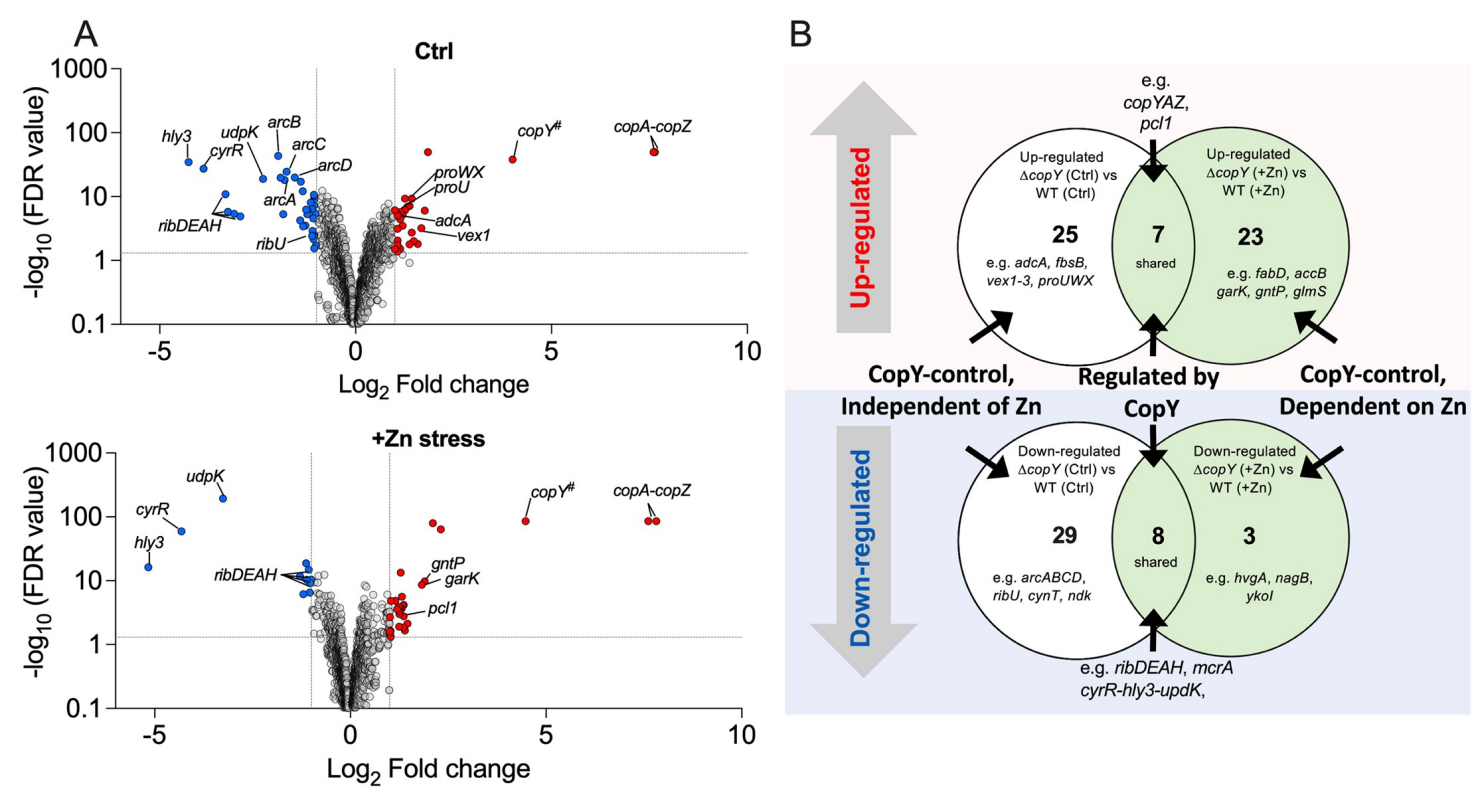
The CopY-responsive GBS transcriptome and the impact of Zn stress. (A) Volcano plots showing data from RNASeq of WT GBS cultures compared to Δ*copY* GBS in THB (Ctrl), or THB supplemented with 0.25 mM Zn (+Zn stress). Transcripts up- or down-regulated in response to Zn (n = 4, >± 2-fold, FDR <0.05) are highlighted in red and blue, respectively. Dotted lines show False discovery rate (FDR; q-value) and fold change cut-offs. Grey points indicate genes that were not significantly changed according to the defined cut-offs; selected genes are identified individually with black lines; # reads mapped to truncated *copY* gene in Δ*copY* strain. FDR values (*y*-axes) were displayed by -log_10_ (Y) transformation followed by plotting on a log_10_ y-axis to generate volcano plots. (B) Venn comparison showing the number of up-regulated and down-regulated genes shared (Regulated by CopY) or unique to control (CopY-control independent of Zn) or +Zn stress conditions (CopY-control dependent on Zn); numbers in Venn comparison exclude non-coding transcripts such as tRNAs. The complete lists of genes and transcripts are available as S1 and S2 Data.

We previously used RNA-seq to elucidate the transcriptome of GBS exposed to Zn stress, which identified >400 differentially expressed genes [21]. Here, we used RNA-seq to dissect the role of *copY* in the bacteria’s response to Zn stress by comparing Δ*copY* GBS to WT in Zn stress. The bacteria were grown in THB or THB supplemented with ± 0.25 mM Zn (not toxic for either strain) to enable cross-strain comparisons independent of bias due to growth-phase. Comparing the transcriptomes of Δ*copY* GBS to WT in the absence of Zn revealed multiple changes in gene expression (±2-fold, P-adj < 0.05) that might explain the sensitivity of Δ*copY* GBS to Zn stress (Fig 5A top panel); in total, 79 targets were significantly altered in expression as a result of *copY* mutation (see S1A Data). For example, in addition to de-repression of *copAZ* (~200-fold up-regulated) in Δ*copY* GBS, *adcA* (encoding a Zn import system [36]) was upregulated 2.3-fold, and there was a ~4-fold reduction in expression of *arcABCD* (encoding an arginine deaminase system that supports GBS survival in Zn stress [21]). Other dysregulated genes encode riboflavin (RF) synthesis (*ribDEAH* ~10-fold down, see below) and transport (*ribU* ~2-fold down), and a surface-associated virulence factor (*fbsB* 2.5-fold up) (S1A Data). Of note was a three-gene locus encoding *cyrR* (here, termed *c**op**Y*-*r*esponsive *R*egulator), *hly3* and *updK*, which was down-regulated (9 to 20-fold; (Fig 5A, S1A Data); in *E*. *coli*, CyrR is part of the MerR superfamily that includes Zn- and Cu-responsive regulators ZntR and CueR; *hly3* encodes a putative hemolysin III/membrane protein but has not been investigated in GBS.

Comparing the transcriptomes of Δ*copY* GBS and WT in Zn stress revealed 78 transcripts that were significantly altered (±2-fold, P-adj < 0.05; Fig 5A bottom panel, S1B Data). We next separated the genes up-regulated from those that were down-regulated in both comparisons of Δ*copY* GBS to WT and identified a subset of genes (7 up-regulated, 8 down-regulated) that were shared amongst both datasets. These included *copY-copA-copZ*, *cyrR-hly3*, *pcl1*, *mcrA* and *ribDEAH*, which we reasoned were likely to be directly regulated by CopY (Fig 5B, S1B Data). We identified a further 25 genes up- and 39 down-regulated that were likely controlled by CopY, but independent of Zn stress (Fig 5B, S1B Data), given they were not differentially expressed in the +Zn stress comparisons. Conversely, there were 23 up- and 3 genes down-regulated, likely representative of genes controlled by CopY and dependent on Zn stress (Fig 5B), since these were not present in the analyses in the absence of Zn stress. Interestingly, *adcA*, *arcABCD* and *ribU* were among those no longer responding to Zn stress in Δ*copY* GBS (Fig 5B, S1B Data). A complete list of the data presented in the Venn diagrams for comparative analyses are available as S2 Data.

### The *covR*-driven transcriptome of GBS in Zn stress

**Fig 6:**
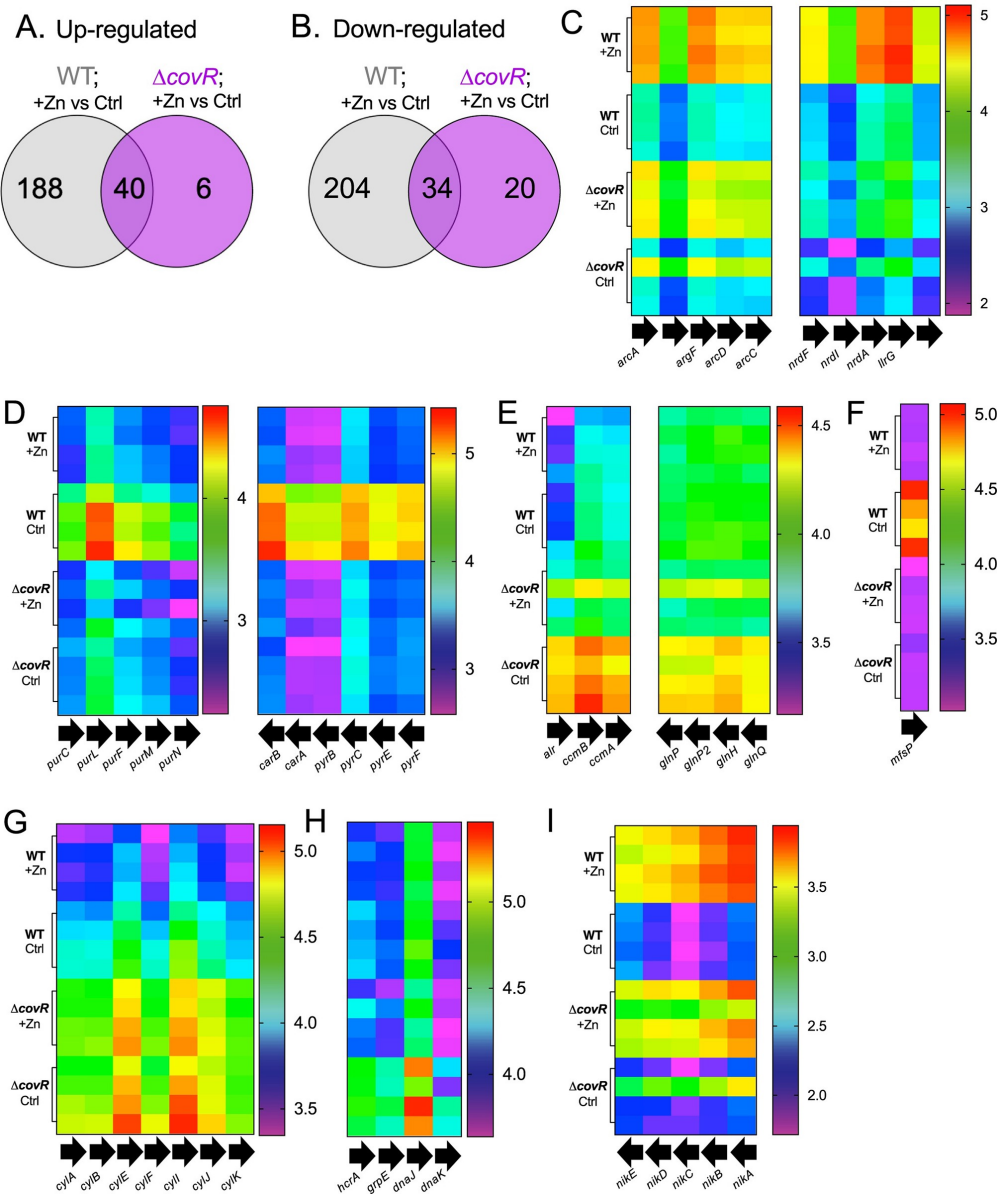
Effect of CovR on GBS transcriptional responses to Zn stress. Venn diagrams comparing the number of up-regulated (A) and down-regulated (B) transcripts that are shared or unique in WT and Δ*covR* GBS strains during exposure to Zn stress (0.25 mM; +Zn) versus control (THB medium alone). (C to I) Heatmaps displaying log_10_ FPKM values (fragments per kilobase million) of selected genes from RNA-sequencing of Δ*covR* and WT GBS with and without Zn as indicated. Columns represent individual genes and rows are biological replicates (*n* = 4), with legends showing colours in log_10_ FPKM for each panel as indicated. Black arrows indicate orientation of genes within each loci. The complete lists of genes and transcripts are available as S3 and S4 Data.

Our discovery of an unexpected role for CovRS in GBS resistance to Zn stress prompted us to examine the effect of *covR* mutation on susceptibility to Zn stress at the transcriptional level. RNAseq analysis of Δ*covR* GBS responses to Zn stress (0.25 mM) and control cultures without Zn supplementation identified 94 differentially regulated transcripts in Δ*covR* GBS; representing a more restricted transcriptional response in Δ*covR* GBS compared to WT GBS that exhibited a total of 467 differentially regulated transcripts in a prior comparison using identical conditions [21]. The transcripts regulated in the WT in response to Zn stress that were lost in the response of the Δ*covR* background are summarized in Figs 6, S7 and shown in S3 Data. Of 229 genes up-regulated in WT [21], 40 were similarly up-regulated more than 2-fold in Δ*covR* GBS (p-adj < 0.05); including genes that are known to respond to Zn stress (Fig 6A) including *czcD*, *mtsA*, *arcA*, and those that are likely involved in controlling detoxification of oxidative and heavy metal stress *(soxR*, *merR*). Among 188 up-regulated genes unique to the WT strain (S4B Data), 168 were expressed < 2-fold, with the remaining 20 not statistically significant, including multiple systems that could contribute to Zn stress resistance, including for Mn and Ni transport (*mtsBC*, *nikABCD*, *mntH2*) and metabolism and transport of ornithine (*arcB*, *arcD*). Six genes were significantly up-regulated in the Δ*covR* analysis but were absent from the WT analysis including putative transport genes for multidrug resistance (*ccmBA*) and glutamine (*glnH*, *glnP*). There was a notable reduction in the number of genes down-regulated (Fig 6B) in response to Zn stress in Δ*covR* GBS (S4C Data); of 238 genes in WT, only 34 were also down-regulated in Δ*covR* GBS, including *ribDEAH*, *adcA*, and *proUWX* and 20 unique responses in Δ*covR* GBS. Genes for purine and pyrimidine nucleotide synthesis (*pur*, *pyr*, *guaC*, *uraA*), a putative major facilitator protein (*mfsP*) strain as well as the *cyl* genes (Fig 6C–6I) no longer responded to Zn in the Δ*covR* (S4 Data C). We undertook an approach to dissect the lists of differentially expressed genes as a result of *covR* mutation and Zn stress in a similar fashion to those presented above for Δ*copY*. These analyses are presented in S7 Fig and S4D and S4E Data and show defined transcriptional changes that are associated with i) regulation by CovR, ii) CovR-control independent of Zn stress, and iii) CovR-control dependent on Zn stress. Taken together, these data show that *covR* is a key regulator of the global transcriptional responses of GBS to Zn stress.

Several genes were subsequently analyzed using qRTPCR to validate the responses of Δ*copY* GBS to Zn stress. We used WT GBS as a baseline to confirm selected Zn-dependent cross-system transcriptional effects that are controlled by CopY. This revealed five patterns of gene dysregulation, reflective of CopY cross-system effects, which are summarized in S8 Fig; the patterns were Zn-induced genes that CopY (i) represses (*e*.*g*., *garK-gntP*) or (ii) activates (*e*.*g*., *ykoI*); (iii) Zn-repressed genes that CopY activates (*e*.*g*., *pcl1*), (iv) genes subject to Zn- and CopY-dependent de-repression (*e*.*g*., *ribDEAH*); and (v) genes activated by CopY irrespective of Zn (*e*.*g*., *cyrR-hly3-udpK*). *ribD* and several other targets subject to CopY regulation (*e*.*g*., *pcl1*, *ykoI* and *hvgA*) were likely co-regulated by CovR because their transcription was altered comparing WT to Δ*covR* GBS (S8 Fig, S3 Data), as previously noted for *hvgA* and *rib* in ST-17 GBS [26]. Thus, *copY* plays a key role the the Zn stress response of GBS by regulating the transcriptional activity of a large number of genes; some of these that are regulated by *copY* cued by Zn stress effect GBS growth. Transcriptional co-regulation of Cu and Zn export responses by *covR* provides auxiliary control beyond *copY* to manage metal stress in GBS.

### Analysis of *ribD* (RF synthesis) and *hly3* (putative hemolysin III/membrane protein)

**Fig 7:**
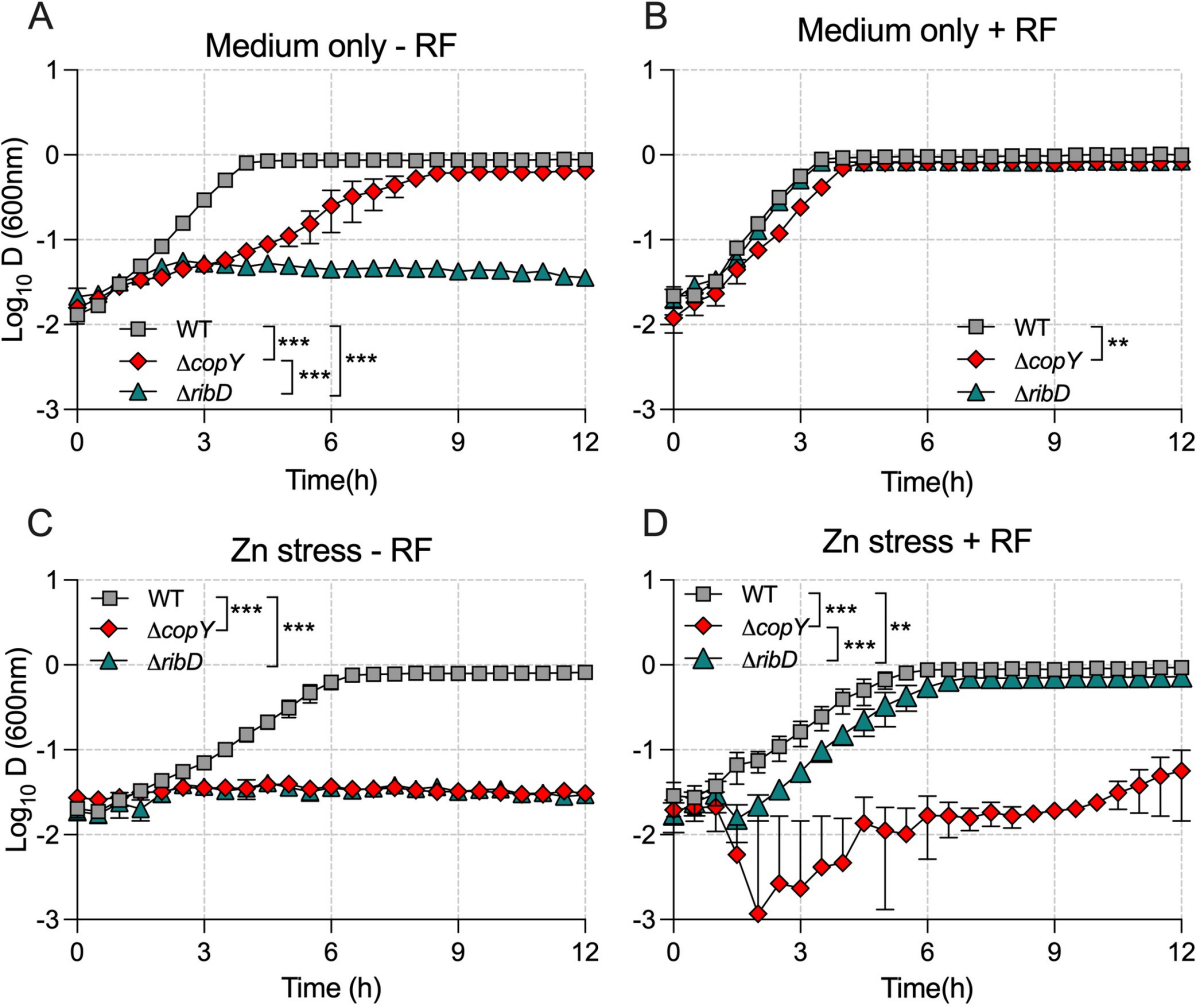
The effect of riboflavin on Zn stress resistance in GBS. WT, Δ*copY* and Δ*ribD* mutants were grown in MDM without supplemental RF (A), MDM supplemented with 0.5 mg/L RF (B), MDM supplemented with 0.1 mM Zn but without supplemental RF (C), or MDM supplemented with 0.1 mM Zn and 0.5 mg/L RF (D). Strains were compared by Area under the Curve analysis followed by ordinary one-way ANOVA and Holm Sidak multiple comparisons with significance between strains indicated via the legends in each panel (*P < 0.05, ** P < 0.01, ***P < 0.05). Data are mean ± S.E.M (*n* = 3 biological repeats) measures of attenuance (*D* at 600_nm_).

The *ribDEAH* operon encodes a putative RF synthesis pathway in GBS, and we found these genes were down-regulated in response to Zn stress in WT GBS in a prior study [21]. The down-regulation of *rib* genes in response to *copY* mutation in this study hints at a connection between CopY and Zn stress that might affect metabolism. To define the outcome of the transcriptional response of *ribDEAH* (considering its activation state was regulated by both Zn and CopY) we used an isogenic mutant in *ribD* and examined its growth in Zn stress. This approach required the synthesis of a Modified Defined Medium (MDM; *Materials and Methods* and S1 Table) to examine growth in a defined medium completely devoid of RF. In MDM lacking RF, Δ*copY* GBS was significantly attenuated (P < 0.001) and Δ*ribD* GBS did not grow at all (P < 0.001), in contrast to WT (Fig 7A). Supplementation of the medium with 0.5 mg/L RF completely restored growth to the Δ*ribD* strain, and there was a modest but significant attenuation (P = 0.01) in the Δ*copY* strain vs WT (Fig 7B). Growth curves generated from titration assays that used RF at 2.5 mg/L and 5.0 mg/L were essentially identical to those at 0.5 mg/L RF (personal communication, M.J. Sullivan). In conditions of Zn stress (0.1mM, without added RF), neither the Δ*ribD* nor Δ*copY* GBS grew in MDM at all (P < 0.001); entry into stationary phase was delayed for the WT but otherwise growth was unaffected (P = 0.14) at this Zn concentration (Fig 7C). In conditions of Zn stress in medium replete with 0.5 mg/L RF there was moderate attenuation (P < 0.001) of Δ*ribD* GBS but the impact of Δ*copY* mutation was more severe (Fig 7D). Thus, RF synthesis in GBS supports resistance to Zn stress and the Δ*copY* strain is attenuated for growth in the absence of RF.

To analyze the effect of an additional *copY*-regulated target cued by Zn stress in GBS on bacterial growth, we mutated *hly3* and compared growth in Zn stress using nutritive and nutrient-limited media. The Δ*hly3* strain was significantly impaired for growth in THB (S9A Fig) but this attenuation was absent from comparisons of WT to Δ*hly3* GBS in Zn stress in THB (S9B Fig) and CDM (S9C and S9D Fig). These data suggest that *hly3* contributes to growth activities in THB but not CDM, and these growth activities are disrupted during Zn stress. Thus, *hly3* is a target of *copY* in response to Zn stress, and it likely contributes to the growth of GBS in certain conditions.

### CopY is conserved among streptococci and supports virulence

**Fig 8:**
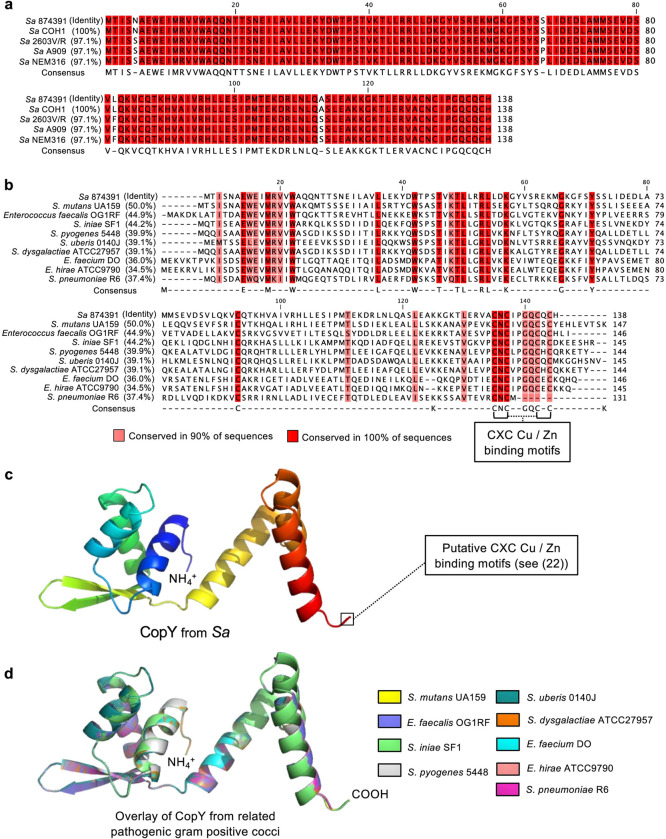
CopY likely operates as a cross-system regulator in numerous gram-positive bacteria. Alignment of CopY shows a high degree of conservation (>97% identity between reference *S*. *agalactiae* strains) (a). Alignment of *S*. *agalactiae* CopY with other *Streptococcus* and *Enterococcus* strains. Highlighted are two conserved putative CXC motifs that are predicted to bind Cu and/or Zn at the C-terminus; amino acids that are >90% conserved are shaded in red, as indicated (b). Predicted structural model of *S*. *agalactiae* CopY and putative region of metal binding at the C-terminus of the protein (c). Structural alignments of predicted CopY proteins from *Streptococcus* and *Enterococcus* strains indicate overlapping protein conformation despite modest conservation of amino acid identity (d).

**Fig 9:**
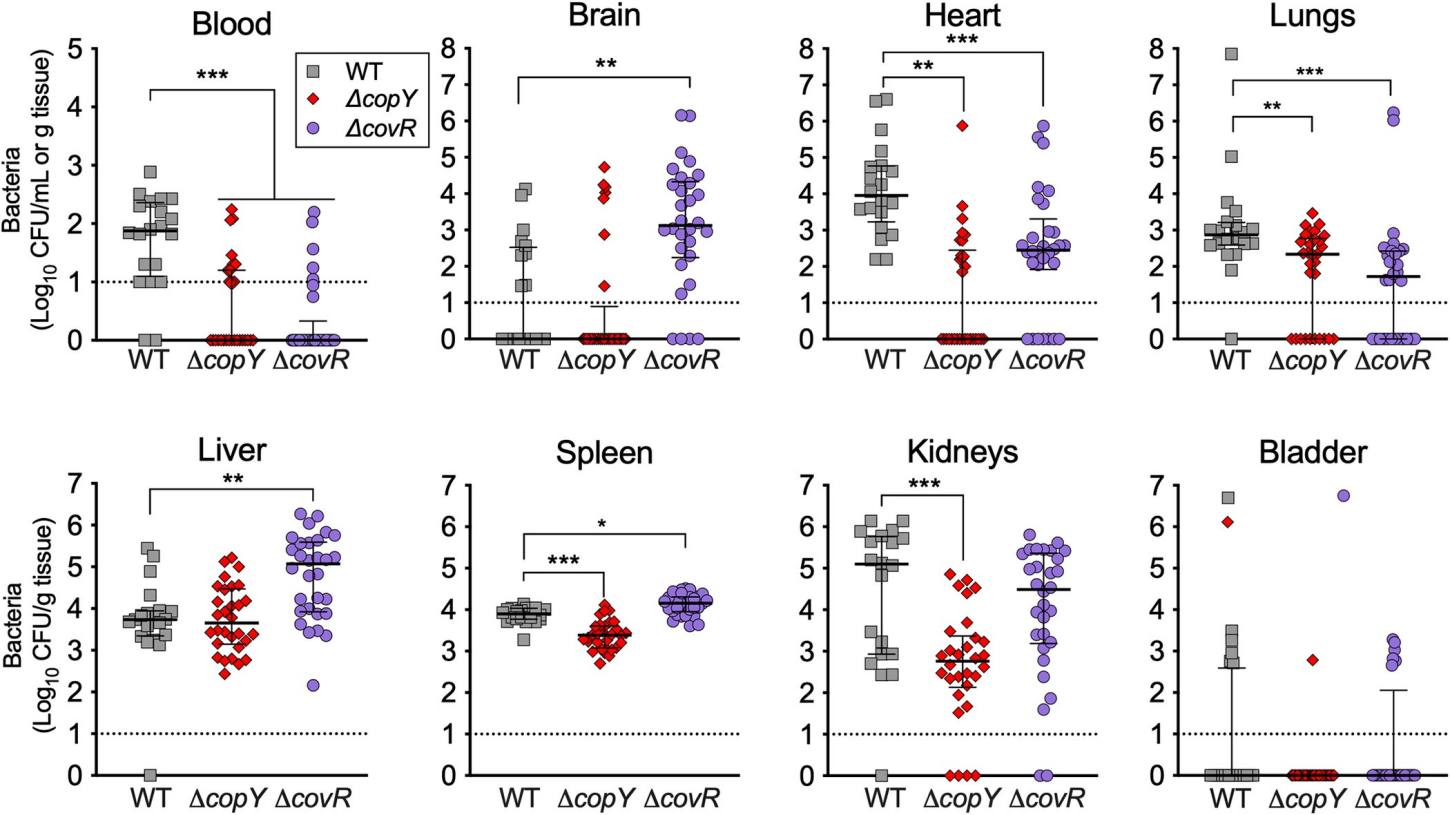
Mutations in *copY* and *covR* have major implications in colonization and disseminated spread of GBS bloodstream infection. Virulence of WT (grey squares), Δ*copY* (red diamonds) or Δ*covR* GBS (purple circles) in a mouse model of disseminated infection. C57BL/6 mice (6–8 weeks old) were intravenously injected with 10^7^ bacteria; bacteremia and disseminated spread of bacteria to brain, heart, lungs, liver, spleen, kidneys and bladder were monitored at 24h post infection. CFU were enumerated and counts were normalized using tissue mass in g. Viable Cell counts of 0 CFU/mL were assigned a value of 1 to enable visualisation on log_10_ y-axes. Lines and bars show median and interquartile ranges and data are pooled from 2–3 independent experiments each containing n = 10 mice; groups infected with mutants were compared to WT group using Kruskal-Wallis ANOVA with Dunn’s corrections for multiple comparisons (*P < 0.05, **P < 0.01, *** P < 0.001).

Identification of a novel function of *copY* that confers a transcriptional mechanism of cross-system control in GBS to respond to Zn stress prompted us to examine its conservation among other pathogenic streptococci, and determine whether it contributes to pathogenesis. Sequence analysis and structural modelling revealed that *copY* is highly conserved among multiple GBS strains, as well as other pathogenic *Streptococcus* spp., and other pathogenic gram-positive cocci (Fig 8). This modelling enabled a schematic representation of the putative metal binding site at the C-terminus of GBS CopY (Fig 8). In the absence of any reported role for *copY* in the pathogenicity of any bacterial species, we tested whether *copY* contributes to GBS virulence. Remarkably, *in vivo* assays based on systemic disseminated infection in mice showed that *copY* was essential to GBS virulence; Δ*copY* GBS was severely attenuated in the blood, heart, lungs, spleen and kidneys of mice at 24h post-inoculation (Fig 9). Expectedly, Δ*covR* GBS was also attenuated in the blood, heart and lungs but was recovered in higher numbers from brain and liver compared to WT (Fig 9). Thus, *copY*, similar to *covR*, can function to support bacterial virulence.

### Forward genetic screen for mediators of GBS resistance to Zn stress

**Fig 10:**
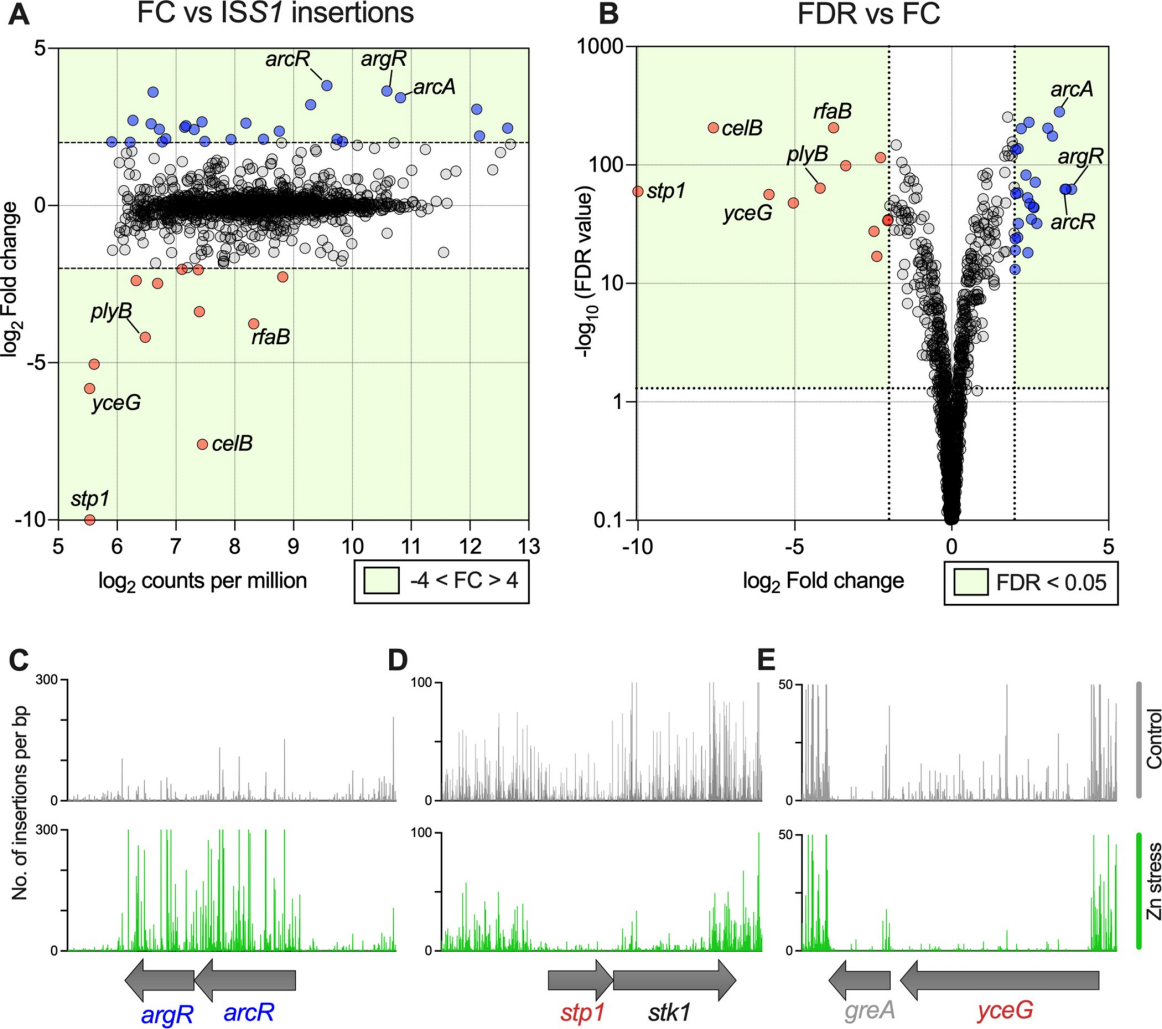
Defining the Zn stress resistome of GBS to identify novel factors in bacterial responses to Zn intoxication. A super-saturated IS*S1* GBS insertion library was subjected to Zn stress and compared to control incubation without Zn to define the Zn resistome. Transposon-directed insertion sequencing (TraDIS) identified 26 genes over-represented (blue) and 12 under-represented (red) during Zn stress. Plots showing Fold change (FC) of each gene compared to number of IS*S1-*insertions per million reads mapped (A) and false discovery rate (FDR; q-value) compared to fold change (B), with green shading indicating genes that satisfied cutoffs as indicated (fold-change ±4; FDR < 0.05). Illustrative read-mapping of IS*S1* insertion sites (C-E) displaying differences between non-exposed control (grey) or Zn stress conditions (green) for selected genes; over-represented *argR/arcR* (C) and under-represented *stp1/stk1* (D) or *yceG* (E). Vertical lines in C-E represent pooled read counts at each base within each locus, with coding sequences of genes represented by grey arrows beneath. Data are compiled from 3 independent experiments. FDR values (Y) were displayed by -log_10_ (Y) transformation followed by plotting on a log_10_ y-axis to generate volcano plot (B).

**Fig 11:**
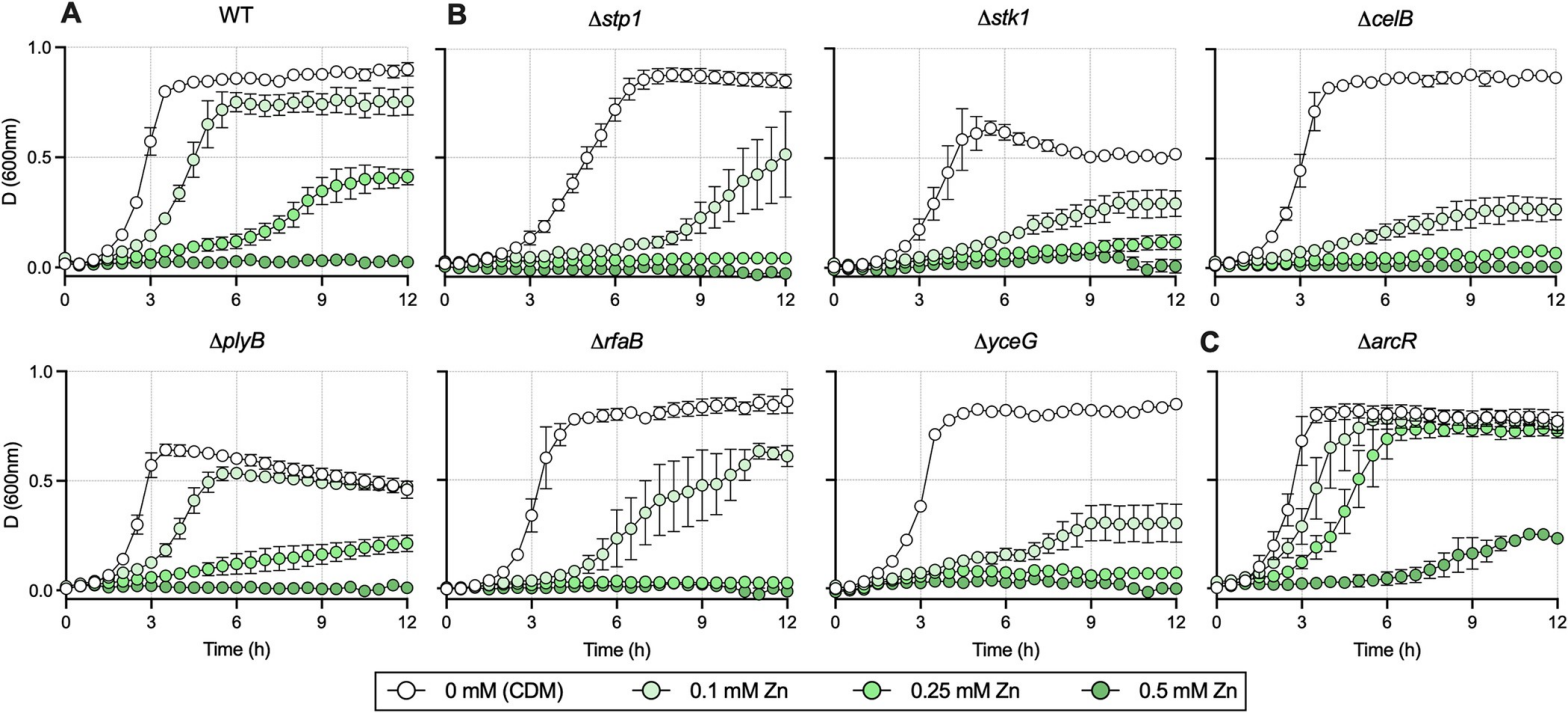
Validation of TraDIS hits by isogenic mutation and phenotypic comparisons for selected genes of the Zn stress resistomes. WT GBS (A) and mutants with deletions in genes identified as important during in Zn intoxication by TraDIS (B-C) were examined for growth phenotypes in CDM (a nutrient-limited medium) or CDM supplemented with 0.1, 0.25 and 0.5mM Zn as indicated. Points show means of attenuance (600nm) and bars show s.e.m. (*n*≥3).

To examine the entire GBS genome for functionally related regions that contribute to resistance to Zn stress we used an open-ended approach based on a super-saturated ~480,000-mutant library, generated using pGh9-IS*S1* [37]. We exposed the bacteria to Zn stress, comparing to non-exposed controls *en masse*. Stringent selection criteria (±4-fold, P-adj < 0.05) identified 12 genes that were essential for GBS to survive during Zn stress; insertional site mapping revealed the frequency of insertions was significantly under-represented in these 12 genes (Fig 10 and S5 Data). Conversely, 26 genes for which the mapped insertions were over-represented were identified, suggesting that these constrain GBS growth in Zn stress. Representative mapping is shown for selected genes in Fig 10C–10E. To validate these hits, we generated targeted isogenic mutants of several candidate genes of the Zn stress resistome, including *stp1* and *stk1* (CHF17_00435 and CHF00436; serine/threonine phosphatase and kinase pair), *celB* (CHF17_01596; EIIC disaccharide transporter), *rfaB* (CHF17_00838; glycosyltransferase), and *yceG* (CHF17_01646; *mltG*-like endolytic transglycosylase). In comparing the growth of WT GBS to mutants in Zn stress, we observed attenuation in all mutants (for under-represented genes) (Fig 11A and 11B). Notably, some mutants exhibited growth defects in the absence of Zn stress (*e*.*g*., Δ*stp1*, Δ*stk1* and Δ*plyB*). Mutation of *arcR* (over-represented in TraDIS) showed a hyper-resistance phenotype; Δ*arcR* GBS grew in high Zn (CDM with 0.25 mM; Fig 11C), which approached inhibitory for the WT (Fig 11A). We complemented the phenotypes *in trans* for Δ*rfaB*, Δ*yceG*, Δ*stp1* and Δ*plyB* (S10A Fig), and used TPEN to restore growth to WT levels in assays with Δ*arcR* and Δ*celB* (S10B Fig). Together, these findings identify a suite of genes in the GBS genome that contribute to the bacteria’s ability to resist Zn intoxication, and which function either by supporting or constraining growth of GBS.

## Discussion

One important function of metalloregulatory proteins is to bind and respond to cognate effectors, while ignoring non-cognate (competing) metals. In bacteria, this functional feature facilitates co-ordinated expression of metal acquisition systems in conditions of metal limitation, whereas, during metal excess, it enables bacteria to drive efflux systems to underpin divergent, contrary responses and resist metal intoxication [3]. The dogma of the function of the CopY transcriptional regulator in bacteria has until now centred on the management of Cu homeostasis via direct effects on the *cop* operon, in response to cues from its cognate effector, Cu. Despite the essential role of CopY in Cu homeostasis in bacteria, a hypothesis that CopY is nonresponsive to competing metals has not directly been addressed until now. The key findings of this study on cellular responses to Zn stress in GBS include (i) a new and biologically consequential function of *copY* in conferring bacterial resistance to Zn intoxication, (ii) robust regulatory inputs from *copY* in response to Zn stress cues, which drive cross-system effects to support bacterial Zn homeostasis, (iii) a virulence function of *copY* that promotes GBS survival in macrophages and during acute disseminated infection, (iv) recognition of CovR as a key player in mediating GBS responses to Zn intoxication, and (v) a defined 38-member family of targets in GBS that comprise the Zn resistome. Overall, this study shows that *copY* controls discrete systems for Cu and Zn homeostasis in *Streptococcus* and establishes a collection of novel genomic elements that enable the bacteria to survive Zn intoxication. More broadly, these findings show that GBS engages CopY in response to multiple metal stress cues to co-ordinate gene expression responses beyond the *cop* operon to support bacterial survival during metal stress.

Recently, we showed that Cu stress in Δ*copY* GBS leads to increased cellular Zn content [20]. The transcriptional landscape of GBS in response to Zn stress, as defined in the present study, elucidates a CopY-regulon of Zn-responsive targets, representing the first described in a bacterial pathogen and supporting a regulatory link between CopY and both Cu and Zn stress. In defining the cross-system effects of CopY that stem from exposure to Zn stress, this study confirms that *copY* in GBS controls robust expression of all the genes that make up the *cop* operon via a mechanism of de-repression in the presence of Cu [19]. We found that *copY* also likely regulates a small but distinct group of additional targets, including multiple genes that have no known links with metal stress responses in bacteria nor virulence (*e*.*g*., *hly3*, *cyrR*, *ribD*, *ykoI*, *garK*). One of these, CyrR, is of the MerR superfamily that includes Zn- and Cu-responsive regulators ZntR and CueR of *E*. *coli* [1,38]. Identification of a three-gene locus of *cyrR*, *hly3* and *updK*, which GBS down-regulates in response to Zn stress, supports the hypothesis that this locus responds to various regulatory inputs, as reported previously [39,40]. Intriguingly, in our study, this response required intact *copY*, revealing a novel mechanism of transcriptional control of the *cyrR*-*hly3*-*updK* locus. The transcriptomic response of Δ*copY* GBS to Zn stress whereby the expression of *cyrR*-*hly3-udpK* is down-regulated ~20-fold (the most significantly differentially expressed in our RNAseq datasets) leads us to hypothesise that *cyrR*-*hly3-udpK* are directly regulated by CopY. We further suggest that CopY regulates *cyrR*-*hly3-udpK* in an opposing fashion to *copYAZ* because CopY appears necessary to activate (rather than de-repress) *cyrR*-*hly3-udpK*. CyrR is a putative metalloregulator and member of the MerR-family of metal-binding transcriptional regulators, and so it will be of interest to study the direct and indirect actions of CopY with DNA-binding assays and any potential role for CyrR in metal ion resistance.

Another locus of the CopY-regulon of Zn-responsive targets, *ribDEAH*, supports RF biosynthesis; *ribD* has no prior known links with Zn in bacteria [41,42]. Testing of a targeted mutant for *ribD* revealed a contribution of RF synthesis to resisting Zn stress, and impairment in growth of the Δ*copY* strain related RF-linked metabolism. Riboflavin supports an array of metabolic processes because it’s downstream products are flavin coenzymes, flavin mononucleotide (FMN) and flavin adenine dinucleotide (FAD), as required for oxidative metabolism and other processes. In addition, FMN can act as a precursor to cobalamin synthesis [43] and FMN as a product of the RF synthesis pathway is detected by FMN-sensing riboswitches that likely control *rib* gene expression in GBS by transcriptional termination [44,45]. GBS contains a putative homologue of a RF import protein RibU (ASZ01710.1) which is down-regulated in the Δ*copY* background (S1 Data) and likely represents a CopY-controlled factor independent of Zn stress (S2 Data); there is evidence to show that CovR also controls *ribDEAH* and *ribU* (S3 Data). Precisely how RF synthesis, versus uptake (in bacteria such as GBS that can do both), contributes to attenuation of growth during Zn stress will need to be examined in future work. In addition, the potential for CopY and CovR to influence the expression of *rib* genes could be contextualised by quantification of cellular FMN and further detailed studies on *rib* transcription and translation during Zn intoxication. In *S*. *pyogenes*, well studied for Zn intoxication resistance [11,15,46], the *ribDEAH* genes are absent, such that the organism relies solely on RF import [47]. In *S*. *pneumoniae*, differential expression of *ribDEAH* leads to differences in host responses to different clinical isolates [48].

In analyzing the function of *copY* compared to the Zn responsive regulator *sczA*, this study reveals the opposing nature of these two metalloregulatory proteins. The former responds to non-cognate Zn cues, but the latter is essentially non-responsive to Cu. Our observation that the *copY* mutant has enhanced *adcA* expression implies Zn-deficiency, however, this mutant shows simultaneous non-altered *czcD* expression, which implies a functional Zn efflux system. These findings might reflect mechanisms beyond transcriptional influences that converge to effect cellular Zn regulation (*i*.*e*., import and export) in ways independent of the SczA-CzcD efflux pathway. On the one hand, our qRTPCR data show *copY* influences the expression of *sczA* but our transposon screen of genes that contribute to resisting Zn intoxication did not identify *sczA* or *czcD*; considered together, we suggest these findings point to additional factors beyond the SczA-CzcD efflux pathway that contribute GBS management of Zn homeostasis. In two other bacterial pathogens, Zn responsive genes other than *sczA* and *czcD*, which contribute to cellular management of Zn levels have been described; for example, in uropathogenic *Escherichia coli* [49] several genes (*e*.*g*., *cpxR*, *envC*, *envZ*, *ompR*, *pstB*, *ldcA*, *yciB*, and *tolB*, which share commonality in being linked to stress responses associated with perturbation of membrane integrity, appear to contribute to susceptibility to Zn; four putative Zn resistance efflux pumps that are responsive to Zn stress are identified in *Acinetobacter* [14]. It is perhaps relevant that GBS is considered distinct in possessing three Zn-binding proteins, AdcA, AdcAII, and Lmb, as discussed elsewhere [50], which were studied in the context of Zn stress and calprotectin that binds Zn and thereby restricts bioavailability for bacteria. That study highlighted *cadD* as contributing to fitness for growth in media containing calprotectin [50] and several other genes in GBS encoding metal transporters implicated in survival in calprotectin.

**Fig 12:**
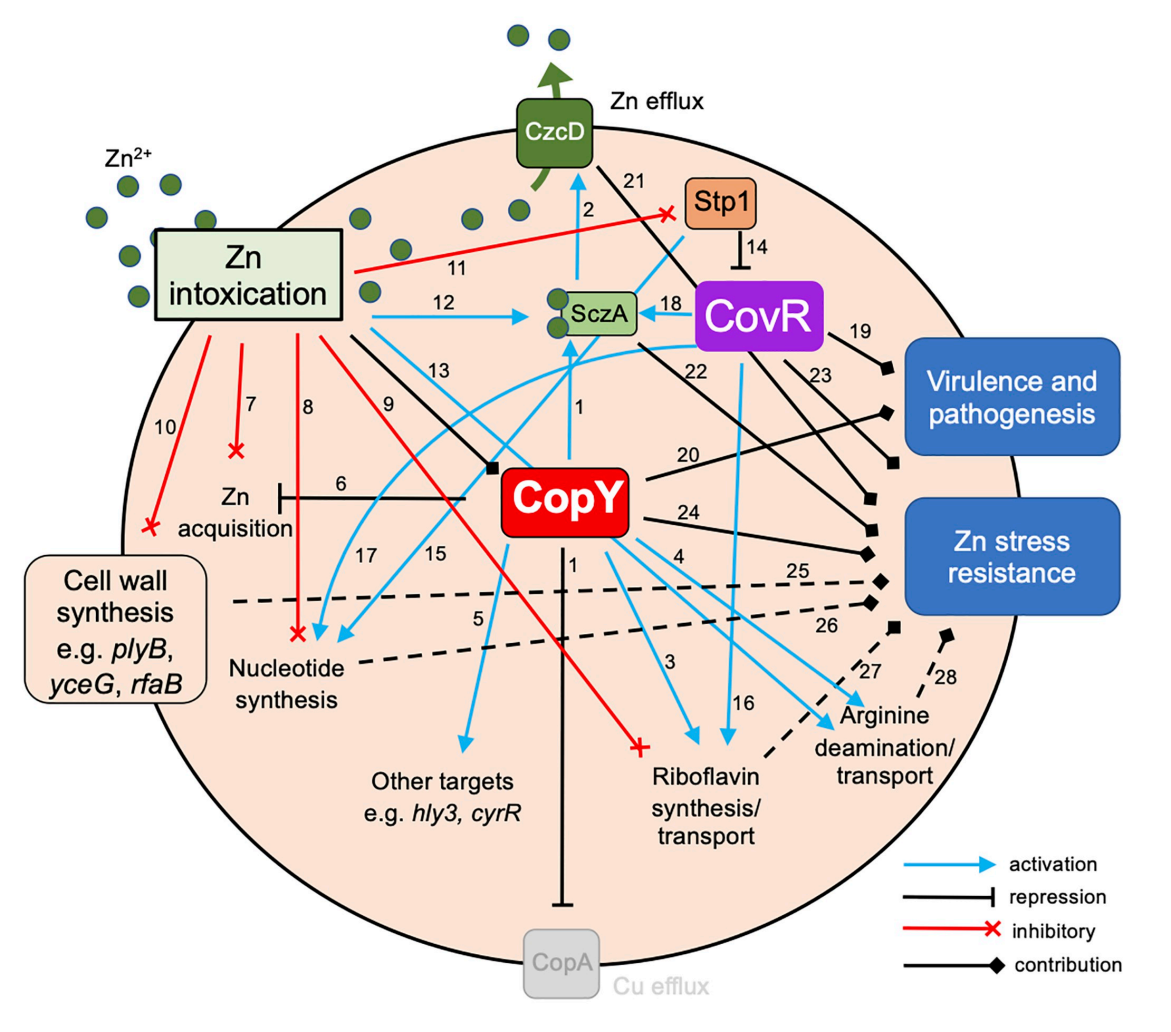
A genetic network in GBS for resisting Zn intoxication. CopY represses *copA* but also activates *sczA* expression^1^; which in turn controls *czcD* for Zn efflux^2^; CopY also activates riboflavin synthesis and/or transport^3^, arginine deamination and transport^4^, other targets^5^ and represses *adcA*^6^. Zn intoxication is inhibitory towards Zn acquisition^7^, *de novo* nucleotide^8^ and riboflavin synthesis^9^ pathways, cell wall synthesis genes^10^ and Stp1^11^; Zn intoxication activates *sczA*^12^ and arginine deaminase^13^. Stp1 represses activity of CovR^14^ and activates de novo purine synthesis^15^. CovR has activation roles for riboflavin^16^ and nucleotide synthesis genes^17^ and *sczA*^18^; Virulence and pathogenesis require contributions from CovR^19^ and CopY^20^. Resistance to Zn stress requires functional *czcD*^21^, *sczA*^22^ *covR*^23^, *copY*^24^ and there are yet to be determined mechanisms of cell wall synthesis genes^25^, nucleotide^26^ and riboflavin synthesis^27^ and arginine deamination^28^ processes that contribute to Zn resistance in GBS. Dotted lines indicate contributions that remain to be characterized. Terms activation and repression refer to gene or protein regulatory activities, inhibitory and contribution refer to pathway or process level interactions. Interactions are based on gene expression data, phenotypes of isogenic mutants, cell and animal assays or prior published literature [20,21,28,35,92,93].

We demonstrate that GBS in Zn stress utilizes CopY to regulate the pools of cell-associated metals in addition to Cu, and that CopY supports survival inside macrophages. This supports a model in which CopY facilitates crosstalk in controlling both Zn and Cu stress by specific and functionally distinct actions depending on the Zn or Cu status of the bacterial and/or host mammalian cell. In this way, CopY represents a connection of different metal management systems in GBS; integrating the findings of the present study on Zn stress with those described for Cu stress enables us to a propose a model of how GBS integrates metal management through CopY (Fig 12). In this model, CopY and CovR are proposed to contribute to the regulation or inhibition of processes and pathways (*e*.*g*., arginine, RF, nucleotide metabolism) as well as activation and repression of specific gene targets effecting Zn resistance and virulence factor function. We used a consensus operator sequence RNYKACANNYGTMRNY for the CopR-CopY family of bacterial Cu exporter system repressors, as described by O’Brien et al. [18], to probe the genome of GBS 874391 for potential CopY binding sites. We found a total of eighteen potential binding sites among which, only four were upstream of genes and in the correct orientation; among these four, two were located upstream of *copY* (the distal and proximal operator sites); all other genes with promoter-proximal sequences that matched the consensus *cop*-operator were not detected as significantly changed in expression in our transcriptomic studies. Taken together, we consider this suggestive that this sequence alone might be required, but not sufficient, for CopY binding to genomic targets in GBS.

CopY is a member of the BlaI superfamily of transcriptional regulators due to shared homology in the N-terminal DNA-binding domain [51] that bind near-identical operator sequences. The family also includes CopR of *Lactobacillus lactis* [52] and β-lactam and methicillin resistance regulators BlaI and MecI of *S*. *aureus* [53]. In support of our identification of alternate regulatory targets of CopY, the related proteins CopR [52] and BlaI/MecI [54] are reported to *trans-*regulate genes in addition to their cognate targets (*copA* for *Lactococcus* CopR; *blaZ* for *Staphylococcus blaI*), including genes involved in metabolism and regulation [52,54].

Structural modelling of CopY reveals a high degree of conservation among pathogens closely related to GBS, which implies that cross-system effects for management of responses to metal stress at the point of *copY* may operate in other bacteria. In support of this, prior biochemical studies of *S*. *pneumoniae* showed that CopY strongly binds Zn [55,56], leading to homotetramerization and activation/enhancement of DNA binding, implying a potential for altered regulatory outcomes of Zn-bound CopY. It is noteworthy, then, that prior studies [18,55,56] use the *cop* operator sequence upstream of *copY* in binding assays to report the degree of de-repression, or, the expression of *copA*, as a proxy measure of CopY-function *in vitro* or *in vivo*, respectively. This is the commonly accepted view of CopY functioning as a DNA-binding repressor protein (at this locus). Our study now reveals an array of new targets and loci that are potentially regulated by CopY; some of these may require Zn to stabilise CopY [56] instead of Cu, for DNA-binding and regulatory activity. Such interaction between Zn-bound CopY and regions of the genome external to the 5′ end of *copYAZ* may i) utilise different operator sites due to altered confirmation, ii) result in activation, rather than de-repression, of a target gene, and iii) only occur in conditions in which Zn outcompetes Cu for binding to CopY. A caveat to these hypotheses follows that: studies on the biochemical basis for CopY function are predominated by work on *S*. *pneumoniae* CopY which lacks two C-terminal cysteine residues (visible in Fig 8) located in the metal-binding domain of the protein, and, carries two additional cysteine residues at positions 52 and 101 [56]. Thus, *S*. *pneumoniae* CopY may differ markedly in it’s metal binding properties or potential for disulfide bond formation during oligomerization, compared to CopY in other streptococcal (including GBS) and enterococcal pathogens. These aspects of CopY among related bacteria suggest that there will be utility in testing the function of *copY* in response to non-cognate metal stress in other bacterial pathogens.

Characterisation of the Zn stress resistome of GBS in this study identifies multiple targets new to the bacterial metal detoxification field [57]. Several identified as being most strongly associated with GBS survival in Zn stress (*e*.*g*., *arcR*, *rfaB*, *plyB*, *yceG*, *celB*, *stp1*) have not previously been linked to Zn stress responses in any bacteria, with *yceG*, *stp1*, *rfaB* and *rfaB* recently shown to contribute to Cu stress resistance [58]. Similar transposon-directed approaches were used recently to study GBS survival in blood [50,59,60], analysis of Zn acquisition and the role of calprotectin *in vivo* using a murine model of systemic infection by Burcham et al. [50] was particularly informative for our *in vivo* studies that show a role for CopY in systemic GBS infection. The precise nature of Zn and other metal stress conditions *in vivo* in these models is unclear. However, Zn levels in blood serum of infected mice can reach up to 900 μM [9], and Zn is mobilised during systemic inflammation and the acute phase response [61,62]. Furthermore, it exhibits spatial and temporal redistribution in murine lung tissue during *S*. *pneumoniae* infection [63] underscoring the dynamic nature of metal ion mobilization responses during infection *in vivo*. Other approaches using defined murine models, including subcutaneous inoculation leading to dissemination [15], intranasal inoculation, and intraperitoneal inoculation leading to sepsis [64] more broadly support the value in examining murine models for study of gene function in bacterial resistance to metal stress even where the precise nature of the metal stress *in vivo* is undefined.

The suite of genes encoding regulators and putative effectors that confer GBS resistance to Zn stress identified in this study dramatically expands our understanding of metal management in bacteria by offering new insight into the diversity of genes that mediate resistance to Zn intoxication. Our findings show this diversity encompasses genes that encode enzymes for metabolism and cell wall synthesis, transporters, and global transcriptional regulators, for example. In the present study, we interrogated a targeted selection of genes in our Zn TraDIS dataset (*e*.*g*., *plyB*, *yceG*, *celB*, *rfaB*, *stp1*) to determine their contribution to resisting Zn stress, using isogenic mutation and growth curve analysis to confirm susceptibility of these mutants to Zn intoxication. Of note, *plyB*, *yceG*, *rfaB* and *stp1* have been previously reported to contribute to resistance to Cu stress resistance [58], indicating a potential for additional layers of ‘cross-talk’ in cellular responses to Cu and Zn that extends beyond *copY* and *covR*.

Our TraDIS approach identified *arcR* and *argR*, two adjacent regulators that likely co-ordinate arginine deaminase (encoded by *arcABDC*) expression, as constraining Zn resistance in GBS, since IS*S1* insertions were significantly enriched in *arcR* and *argR*. Isogenic mutation in *arcR* enhanced resistance to Zn, consistent with the TraDIS finding. A previous study identified a role for *arcA* in conferring resistance to Zn stress, since an isogenic *arcA* mutation attenuated growth under Zn intoxication conditions [21]. Interestingly, in contrast to this observation, we detected enrichment, rather than reduction, in IS*S1* insertions in *arcA*. This could be explained by a potential for polar effects of IS*S1* insertion on the *arcABDC* locus. It would be of interest to examine the contribution of *arcBC* and *arcD* to Zn resistance, since these encode proteins that produce or import ornithine, which was recently shown to rescue Zn sensitivity in GBS [21].

Analysis of CovR/CovS in the GBS response to Zn stress revealed a role for the Stk1/Stp1-CovR regulation axis in mediating Zn resistance. Stp1/Stk1 phosphorylates CovR to drive its effects [29,65] and we found *stp1/stk1* were essential for Zn resistance. Our transposon screen identified *stp1* as the most significantly under-represented gene in the GBS genome required for resistance to Zn stress. We note that there are reports of the emergence of suppressor mutations in streptococci arising in *stk1* during mutation of *stp1* [66,67] but our approach of complementation based on a strategy of supplying both *stp1* and *stk1 in trans* with the native promoter, similar to that reported elsewhere [68], restored the phenotype of the mutant to WT in relation to Zn stress. These findings support an interpretation that the Stk1/Stp1-CovR regulation axis has a role in mediating Zn resistance in GBS, but we cannot exclude secondary *stk1* mutations in our Δ*stp1* mutant, should this strain be used in any follow-up work relating to the actions of the phosphatase (Stp1) and kinase (Stk1) pair. The Stk1/Stp1-CovR regulatory axis has been linked to streptococcal virulence and nucleotide biosynthesis but has not previously been linked with a response to Zn stress. Intriguingly, a prior study identified the pyrimidine and purine nucleotide biosynthesis pathways (via the *pyr* and *pur* genes) as significantly down-regulated in response to Zn stress [21]. We now provide evidence to show that *covR* is required for activating the *pur* and *pyr* pathways, since their expression is abrogated, regardless of Zn stress status, circumstantially supporting a model in which *de novo* nucleotide biosynthesis, which is required for GBS colonization [69], also contributes to Zn stress resistance. The precise roles of the nucleotide biosynthesis pathways in contributing to Zn stress remain to be elucidated.

That *covR* promotes resistance to Zn intoxication in GBS can be used to suggest a parallel between the *covRS* system and the dual-metal resistance regulatory function of *copY*, whereby the regulator governs resistance of the bacteria to multiple metals. A two-component system in *Caulobacter crescentus*, UzcRS, is highly responsive to both Zn and Cu (and uranium) to couple a response regulator to different extra-cytoplasmic metal stress responses [70]. In *Pseudomonas stutzeri*, overlapping regulation for Cu and Zn resistance genes was recently reported [23], with cross-regulation achieved by a core set of *P*. *stutzeri* Cu and Zn-responsive genes. In *Mycobacterium tuberculosis*, two paralogous ATPases, CtpD and CtpJ that are activated by Co(2+) and Ni(2+) appear to mediate metal efflux, but play non-redundant roles in virulence and metal efflux [71]. Further elucidation of cross-talk mediated by CopY, and CovR as a regulator governing resistance of GBS to multiple metals will be help to more clearly define their functions in comparison to systems in other bacterial pathogens. In particular, further work to tease apart how CovRS functions as a regulator of Zn transcriptional responses in GBS is now warranted.

Bacterial resistance to metal stress is used by some pathogens to evade host defences [12,49]. Cu management contributes to virulence in some infections, but a role for CopY in virulence has not been reported. For example, *S*. *pneumoniae* regulates central metabolism in response to metal stress to support bacterial survival [72], and uses CopA to drive virulence during host infection [17]. In *E*. *coli*, Cu-transporting ATPases, including CopA are required for bacteria survival in an *in vitro* host-pathogen interface in macrophages [73]. We found that *copY* contributes to the virulence of GBS because Δ*copY* GBS was attenuated in multiple organs, including the blood, heart, lungs, and kidneys of mice following systemic infection. These findings are consistent with prior observations that have alluded to a role of CopY in supporting bacterial virulence. For example, increased expression of *copY* in *S*. *pneumoniae* in the lungs of mice was reported [63], and higher Cu levels along with co-incidental up-regulation of *copYAZ* in the blood of mice infected with *S*. *pyogenes* was reported [16]. It may be that GBS encounters elevated levels of Zn in mice during dissemination via the blood, either during interactions with immune cells or in transiting to different tissue microenvironments; such exposure *in vivo* could account for attenuation of the Δ*copY* mutant in this model. Notwithstanding a role of physiological metal concentrations and CopY in the host, it is important to note that we also identified differential expression of some key virulence factors in the Δ*copY* strain, including *hvgA* (encoding hypervirulence adhesin A), *fbsB* (fibrinogen binding streptococcal protein B) and *mcrA* (myosin cross reactive antigen; oleate hydratase) that may account for altered virulence profiles in the Δ*copY* mutant. Our finding of attenuation of the *copY* mutant *in vivo* may be related to exposure of the bacteria to Cu or Zn during infection; however, Δ*copY* GBS overexpresses *copA-copZ* (so should be more resistant to Cu) [20]; additionally, we did not measure tissue levels of metal ions in this study pointing to a need to further characterise the basis of the attenuation of Δ*copY* GBS *in vivo*. Our findings for the Δ*covR* mutant show that this global virulence regulator supports GBS survival but this depends on tissue context; we expected to find elevated bacterial loads in the brain for Δ*covR* GBS, based on a prior study that used similar doses and intravenous inoculation [29], our findings for brain infection are consistent with this prior report. We also note the prior study did not quantify tissue colonisation of the liver, kidneys, heart, or lungs as we report here. The reduction in tissue loads in these organs is consistent with reduced virulence following intraperitoneal injection and a subsequent increase in LD50 for *ΔcovR* strains as reported in [34].

The finding of massive upregulation (~200-fold) of *copA-copZ* in Δ*copY* GBS is notable because this is based on a non-polar, unmarked deletion that we generated in this study. It would be of interest to determine if such transcription is translated into higher amounts of CopA-CopZ proteins to this magnitude. If so, this would cause significant metabolic alterations, including for example the demand on RNA and protein synthesis machinery and a potential for hyper-decoration of the membrane with CopA, which presumably might attenuate GBS growth. A limitation to this part of our study is the absence of analysis of a defined *copYAZ* triple deletion mutant that would enable definitive identification of the contribution of *copA-copZ* overexpression to effects on sensitivity to Zn. Aside from differences in Zn or Cu concentrations *in vivo* (discussed above), such effects could account for attenuated virulence in the Δ*copY* strain. Interestingly, differential insertion of IS*S1* was not detected in *copY* under Zn stress in our transposon screen. This could be accounted for by the potential for polar effects of IS*S1* insertion in *cis*, thus abolishing, rather than up-regulating, *copA-copZ* transcription. Pointedly though, although growth rate of the Δ*copY* strain was reduced in THB medium conditioned for Zn stress (S1A Fig), this strain was able to achieve significant culture densities after 12h of growth (the time at which the transposon pool was processed for insertion sequencing). In support of this, several of the novel Zn resistome targets (*e*.*g*., *rfaB*, *stp1*, *celB*) exhibited a more dramatic attenuation phenotype compared to the Δ*copY* strain (S11 Fig). Future studies might utilise a nutrient-limited medium such as CDM or MDM with TraDIS and Zn stress to yield new factors that support GBS Zn resistance. It is also relevant here to note the influence of culture media on data from distinct *in vitro* assay conditions, and the need to consider this when interpreting such findings, as we discussed elsewhere [20]. Finally, we made several unsuccessful attempts to generate *copY*-deficient mutants in GBS reference strains other than GBS 874391, including COH1 and NEM316 and A909 to analyze *copY*-mutant phenotypes in other GBS background. Thus, it would be of interest in future studies to investigate other GBS strains for the contribution of *copY* to bacterial responses to Zn stress and, in particular, if the regulatory responses reported here are conserved across multiple GBS strains.

In summary this study identifies a new role of *copY* in responding to Zn stress in GBS, revealing novel regulatory cross-talk between this Cu-sensing repressor that results in modulation of Zn homeostasis. The Zn sensitivity phenotype of *copY*-deficient GBS is not attributed to a single Zn-resistance effector (such as modification the CzcD efflux system) but arises from pleiotropic effects that encompass multiple factors that underpin GBS survival during Zn stress. These likely include arginine deaminase expression (via *arcABDC*), Zn import (via *adcA*) RF synthesis (via *ribDEAH*) and an as-yet undefined role for the *cyrR-hly3* locus.

## Materials and methods

### Ethics statement

This study was carried out in accordance with the guidelines of the Australian National Health and Medical Research Council. The Griffith University Animal Ethics Committee reviewed and approved all experimental protocols for animal usage according to the guidelines of the National Health and Medical Research Council (approval: MSC/01/18/AEC).

### Bacterial strains, plasmids and growth conditions

GBS, *E*. *coli* and plasmids used are listed in S2 Table. GBS 874391 is a human vaginal isolate and genome sequenced reference strain of the hypervirulent sequence type 17 lineage that comprises serotype III clones that are associated with a disproportionately high number of cases of invasive neonatal disease, particularly meningitis [74]. GBS was routinely grown in Todd-Hewitt Broth (THB) or on TH agar (1.5% w/v). *E*. *coli* was grown in Lysogeny Broth (LB) or on LB agar. Routine retrospective colony counts were performed by plating dilutions of bacteria on tryptone soya agar containing 5% defibrinated horse blood (Thermo Fisher Scientific). Media were supplemented with antibiotics (spectinomycin (Sp) 100μg/mL; chloramphenicol (Cm) 10 μg/mL), as indicated. Growth assays used 200μL culture volumes in 96-well plates (Greiner) sealed using Breathe-Easy membranes (Sigma-Aldrich) and measured attenuance (*D*, at 600nm) using a ClarioSTAR multimode plate reader (BMG Labtech) in Well Scan mode using a 3mm 5x5 scan matrix with 5 flashes per scan point and path length correction of 5.88mm, with agitation at 300rpm and recordings taken every 30min. Media for growth assays were THB and a modified Chemically-Defined Medium (CDM) [36] (with 1g/L glucose, 0.11g/L pyruvate and 50mg/L L-cysteine), or Modified Defined Medium (MDM; see S1 Table) supplemented with Cu or Zn (supplied as CuSO_4_ or ZnSO_4_) as indicated. Riboflavin supplementation (0.5 mg/L, 2.5 mg/L and 5 mg/L) was performed using MDM; to make MDM devoid of RF, ‘Solution G’ (S1 Table) was excluded and the formulation was supplemented with only 5 mg/L folic acid. For attenuance baseline correction, control wells without bacteria were included for Cu or Zn in media alone.

### DNA extraction and genetic modification of GBS

Plasmid DNA was isolated using miniprep kits (QIAGEN), with modifications for GBS as described elsewhere [75]. Mutant strains (S2 Table) were generated by isogenic gene-deletions, constructed by markerless allelic exchange using pHY304aad9 as described previously [21,76]. Plasmids and primers are listed in S2 and S3 Tables, respectively. Mutants were validated by PCR using primers external to the mutation site and DNA sequencing. Complementation *in trans* was achieved using the *E*. *coli*-streptococcal shuttle vector pDL278 [77,78].

### RNA extraction, qRTPCR

For Cu and Zn exposure experiments, 1mL of overnight THB cultures were back-diluted 1/100 in 100mL of THB (prewarmed at 37°C in 250mL Erlenmeyer flasks) supplemented with 0.25 mM Zn or 0.5 mM Cu. Cultures were grown shaking (200rpm) at 37°C; after exactly 2.5h, 10-50mL volumes containing approximately 500 million mid-log bacteria were harvested; RNA was preserved and isolated as described previously [31]. RNA quality was analysed by RNA LabChip using GX Touch (Perkin Elmer). RNA (1000ng) was reverse-transcribed using Superscript IV according to manufacturer’s instructions (Life Technologies) and cDNA was diluted 1:50 in water prior to qPCR. Primers (S3 Table) were designed using Primer3 Plus [79,80] to quantify transcripts using Universal SYBR Green Supermix (Bio-Rad) using a Quantstudio 6 Flex (Applied Biosystems) system in accordance with MIQE guidelines [81]. Standard curves were generated using five-point serial dilutions of genomic DNA (5-fold) from WT GBS 874391 [74]. Expression ratios were calculated using C_T_ values and primer efficiencies as described elsewhere [82] using *dnaN*, encoding DNA polymerase III β-subunit as housekeeper.

### Whole bacterial cell metal content determination

Metal content in cells was determined as described [10]. Cultures were prepared essentially as described for *RNA extraction*, *qRTPCR* with the following modifications; THB medium was supplemented with 0.25 mM Zn or 0.5 mM Cu or not supplemented (Ctrl), and following exposure for 2.5h, bacteria were harvested by centrifugation at 4122 x g at 4°C. Cell pellets were washed 3 times in PBS + 5mM EDTA to remove extracellular metals, followed by 3 washes in PBS. Pelleted cells were dried overnight at 80°C and resuspended in 1mL of 32.5% nitric acid and incubated at 95°C for 1h. The metal ion containing supernatant was collected by centrifugation (14,000 x g, 30min) and diluted to a final concentration of 3.25% nitric acid for metal content determination using inductively coupled plasma optical emission spectroscopy (ICP-OES). ICP-OES was carried out on an Agilent 720 ICP-OES with axial torch, OneNeb concentric nebulizer and Agilent single pass glass cyclone spray chamber. The power was 1.4kW with 0.75L/min nebulizer gas, 15L/min plasma gas and 1.5L/min auxiliary gas flow. Cu was analysed at 324.75nm, Zn at 213.85nm, Fe at 259.94nm and Mn at 257.61nm with detection limits at <1.1ppm. The final quantity of each metal was normalised using dry weight biomass of the cell pellet prior to nitric acid digestion, expressed as μg.g^-1^dry weight.

### Mammalian cell culture

U937 monocytes were differentiated into human monocyte-derived macrophages (MDMs) as follows. Briefly, monocytes were seeded (5x10^5^ per well such that 1x10^5^ adhere) into the wells of a 96-well tissue culture-treated plate (Falcon) essentially as described elsewhere [83,84], except that U937 monocytes were differentiated by exposure to 30ng/mL phorbol 12-myristate 13-acetate (PMA) for 48h and cells subsequently rested in media without PMA for 72h to enhance morphological and phenotypic markers of MDMs [85]. A multiplicity of infection (MOI) of 100 bacteria: macrophage for 1h was used in RPMI without antibiotics. Non-adherent bacteria were removed by five washes of 200μL PBS using a Well Wash Versa (Thermo Scientific). RPMI containing 250U/mL penicillin, streptomycin (Gibco) and 50μg/mL gentamicin (Sigma-Aldrich) were used for antibiotic protection assays to kill extracellular bacteria as described previously by incubating for 1h at 37°C in 5% CO_2_ [84]. After 1h, 24h or 48h incubation with antibiotics, monolayers were washed five times with 200μL PBS and lysed by brief exposure to 50μL of 0.25% trypsin EDTA (Gibco) and 0.1% Triton-X-100 (10 min) prior to dilution with 150μL PBS and enumeration of CFU/mL by serial dilution and plate counts on agar. Additional assays that ran in parallel were identical except that 20 μM Cu was added to RPMI culture media at all stages post PMA-treatment of U937 cells. Experiments were repeated 3 times independently; CFU per milliter values were derived from 4 technical replicate wells for each strain, condition and timepoint. The survival index for intracellular Δ*copY* GBS was calculated as follows: CFU per milliliter of Δ*copY* / CFU per milliter of WT GBS, for each independent experiment.

### RNA sequencing and bioinformatics

Cultures were prepared as described above for *RNA extraction*, *qRTPCR* to compare mid-log phase WT or Δ*copY* cells grown in THB + 0.25 mM Zn or in THB without added Zn. RNase-free DNase-treated RNA that passed Bioanalyzer 2100 (Agilent) analysis was used for RNA sequencing (RNA-seq) using the Illumina NextSeq 500 platform. We used a Bacterial Ribosomal RNA (rRNA) Depletion kit (Invitrogen) prior to library construction, and TruSeq library generation kits (Illumina, San Diego, California). Library construction consisted of random fragmentation of the RNA, and cDNA production using random primers. The ends of the cDNA were repaired and A-tailed, and adaptors were ligated for indexing (with up to 12 different barcodes per lane) during the sequencing runs. The cDNA libraries were quantitated using qPCR in a Roche LightCycler 480 with the Kapa Biosystems kit (Kapa Biosystems, Woburn, Massachusetts) prior to cluster generation. Clusters were generated to yield approximately 725K–825K clusters/mm^2^. Cluster density and quality was determined during the run after the first base addition parameters were assessed. We ran single-end 75–bp sequencing runs to align the cDNA sequences to the reference genome. For data preprocessing and bioinformatics, STAR (version 2.7.3a) was used (parameters used:—outReadsUnmapped Fastx—outSAMtype BAM SortedByCoordinate—outSAMattributes All) to align the raw RNA sequencing fastq reads to the WT *S*. *agalactiae* 874391 reference genome [74]. HTSeq-count, version 0.11.1 (parameters used: -r pos -t exon -i gene_id -a 10 -s no -f bam), was used to estimate transcript abundances [86]. DESeq2 was then used to normalized and test for differential expression and regulation following their vignette. Genes that met certain criteria (*i*.*e*., fold change of > ±2.0, q value (false discovery rate, FDR of <0.05) were accepted as significantly altered [87]. Raw and processed data were deposited in Gene Expression Omnibus (accession no. GSE167894 for WT *S*. *agalactiae* 874391 control condition; GSE161127 for WT *S*. *agalactiae* 874391 Zn condition; GSE167898 for Δ*covR S*. *agalactiae* 874391 control condition; GSE167899 for Δ*covR S*. *agalactiae* 874391 Zn condition; GSE167896 for Δ*copY S*. *agalactiae* 874391 control condition; GSE167031 for Δ*copY S*. *agalactiae* 874391 Zn condition).

### Animal model

Virulence was tested using a mouse model of disseminated infection based on intravenous challenge with 10^7^ GBS *(*WT, Δ*copY* or Δ*covR*) as described elsewhere [88].

### Transposon Directed Insertion Site Sequencing (TraDIS)

Generation and screening of the 874391:IS*S1* library was performed essentially as previously described [89], with some modifications. Briefly, the pGh9:IS*S1* plasmid (provided by A. Charbonneau *et al*.) was transformed into WT GBS, and successful transformants were selected by growth on THB agar supplemented with 0.5μg/mL Erythromycin (Em). A single colony was picked and grown in 10mL of THB with 0.5μg/mL Em at 28°C overnight. The overnight cultures were incubated at 40°C for 3h to facilitate random transposition of IS*S1* into the bacterial chromosome. Transposon mutants were selected by plating cultures onto THB agar supplemented with Em and growing overnight at 37°C. Pools of the transposon mutants were harvested with a sterile spreader and stored in THB supplemented with 25% glycerol at -80°C. The final library of approximately 470,000 mutants was generated by pooling two independent batches of mutants.

Exposure of the library used approximately 1.9 x 10^8^ bacteria inoculated into 100mL of THB (non-exposed Ctrl) or THB supplemented with 1mM Zn in THB. The cultures were grown for 12h at 37°C (shaking), and subsequently, 10mL of culture were removed and washed once with PBS. Genomic DNA was extracted from three cell pellets per condition (prepared as independent biological samples) using the DNeasy UltraClean Microbial Kit (Qiagen) according the manufacturer’s instructions, except that the cell pellets were incubated with 100 units of mutanolysin and 40mg of RNase A at 37°C for 90min.

Genomic DNA was subjected to library preparation as previously described [89], with slight modifications. Briefly, the NEBNext dsDNA fragmentase (New England BioLabs) was used to generate DNA fragments in the range of 200-800bp. An in-house Y-adapter was generated by mixing and incubating adaptor primers 1 and 2 (100μM, S3 Table) for 2min at 95°C, and chilling the reaction to 20°C by ramping down in temperature by 0.1°C per second. The reaction was placed on ice for 5min, and ice cold ultra-pure water was added to dilute the reaction to 15μM. The Y-adaptor was ligated to the ends of the fragments using the NEBNext Ultra II DNA Library Prep Kit for Illumina (New England BioLabs) according to the manufacturer’s instructions. All adaptor ligated fragments were incubated with *Not*I.HF (New England BioLabs) for 2h at 37°C to deplete plasmid fragments. The digested fragments were PCR amplified as per the protocol outlined in the NEBNext Ultra II DNA Library Prep Kit using a specific IS*S1* primer and reverse indexing primer (S3 Table). DNA quantification was undertaken using a QuBit dsDNA HS Assay Kit (Invitrogen) and purified using AMPure XP magnetic beads (Beckman Coulter). All libraries were pooled and submitted for sequencing on the MiSeq platform at the Australian Centre for Ecogenomics (University of Queensland, Australia).

The sequencing data generated from TraDIS libraries were analysed used the Bio-TraDIS scripts [90] on raw demultiplexed sequencing reads. Reads containing the transposon tag (CAGAAAACTTTGCAACAGAACC) were filtered and mapped to the genome of WT GBS 874391 using the bacteria_tradis script with the “—smalt_y 1” and “—smalt_r 0” parameters to ensure accuracy of insertion mapping. Subsequent analysis steps to determine log_2_ fold-change (log_2_FC), false discovery rate (FDR) and P value were carried out with the AlbaTraDIS script [91]. To identify genes in GBS 874391 required for resistance to Zn intoxication condition used, we used a stringent criterion of log_2_FC ≤ -2 or ≥ 2, FDR <0.001 and P value <0.05. The TraDIS reads are deposited in the Sequence Read Archive (SRA) under BioProject ID: PRJNA674399.

### Statistical methods

All statistical analyses used GraphPad Prism V9 and are defined in respective Figure Legends. Statistical significance was accepted at P values of ≤0.05.

## Supporting information

S1 Table Modified Defined Medium (MDM) components and recipe. (DOCX)

S2 Table Bacterial strains and plasmids used in this study. (DOCX)

S3 Table Primers used in this study. (DOCX)

S1 Fig Growth of GBS in THB using conditions of Zn stress. WT, Δ*copY*, Δ*covR* and Δ*sczA* mutants and corresponding complemented strains (indicated by +C) were grown in THB medium supplemented with 1.5 mM Zn (A) and compared (B) using Area Under the Curve analysis in followed by ordinary one-way ANOVA and Holm Sidak multiple comparisons (** P < 0.01, *** P < 0.005). Growth curves of the bacteria in control conditions (THB medium alone) are shown for comparison (C) and compared (D) using Area Under the Curve analysis in followed by ordinary one-way ANOVA and Holm Sidak multiple comparisons (** P < 0.01, *** P < 0.005). Measures of Attenuance (*D* at 600nm) or Area Under the Curve lines shown are mean ± S.E.M (*n* = 3 biological repeats).
(TIFF)

S2 Fig Complementation of *copY* using expression of *copA* as a measure of CopY-mediated repression of transcription. WT, Δ*copY* and complemented strains (Δ*copY* + C) were grown in THB medium to mid-log phase prior to analysis of *copA* expression using qRT-PCR. Transcript abundance was normalised using *dnaN* and relative mRNA quantity (A) and Fold change (B) values compared to WT were calculated using *dnaN* as housekeeper and ΔΔ^CT^ values incorporated primer efficiency values as previously described [82]. Bars show mean ± S.E.M (*n* = 3 biological repeats). Data were compared using (A) one-way ANOVA with Holm-Sidak multiple comparisons or (B) unpaired *t-*tests B (** P < 0.01, *** P < 0.005).
(TIFF)

S3 Fig Growth of GBS in THB and CDM in conditions of Cu stress. Additional growth analyses of GBS WT (squares), Δ*sczA* (triangles) and Δ*covR* (circles) mutants in rich (THB) or limiting (CDM) medium and subjected to Cu intoxication (filled; 1.5 mM Cu for THB, 1 mM for CDM) vs control conditions (empty; medium alone without supplemental Cu) as indicated. Bars show mean ± S.E.M (*n*≥3 biological repeats). Data (A, B) were compared using one-way ANOVA with Holm-Sidak multiple comparisons (C,D; * P < 0.05 ** P < 0.01, *** P < 0.005).
(TIFF)

S4 Fig Intracellular accumulation of Zn, Cu, Mn, Fe or Mg in Δ*covR* GBS with or without Zn stress. Cells of Δ*covR* GBS were exposed to Zn (0.25 mM; green bars) and cellular metal content was compared to unexposed controls (THB only; white bars) using Inductively coupled plasma optical emission spectrometry (ICP-OES). Metal content was normalised using pellet dry weight (μg.g^-1^ dry weight biomass). Dotted lines indicate mean values from WT GBS from the same condition, published previously [21]. Bars show mean ± S.E.M (*n* = 3 biological repeats). Means were compared to mean data for WT values in the same conditions using ordinary one-way ANOVA followed by Holm-Sidak multiple comparisons (* P < 0.05).
(TIFF)

S5 Fig Absolute recovery of WT and Δ*copY* GBS from human monocyte-derived macrophages. Intramacrophage survival index assays of Δ*copY* vs WT shown in Fig 3 comprise three independent experiments (E1-E3), derived from colony forming units per millilitre (CFU/mL) data of WT (grey squares) and Δ*copY* GBS (red diamonds) quantified following gentamicin protection assays at 1h, 24h and 48h after addition of antibiotics. Blue shading indicate assays performed using RPMI with 20 μM supplemental Cu, and grey shading indicate RPMI without supplemental Cu. Numbers above brackets indicate the survival index from each strain-strain comparison at each timepoint, for each experiment, relating to the individual datapoints shown in Fig 3. Bars show means ± S.E.M and individual technical replicates (n = 4) are shown for each experiment.
(TIFF)

S6 Fig Cross-regulation of Zn and Cu stress responses by *copY* and *covR*. Transcripts of *czcD* (A) and *copA* (B) were quantified by qRTPCR from cultures of WT, Δ*copY*, Δ*covR and* Δ*sczA* mutants supplemented with Zn (0.25 mM) or Cu (0.5 mM) and compared to non-exposed (THB only) controls (*n* = 4). Absolute transcript amounts were normalized using housekeeping *dnaN* and generated from standard curves using GBS genomic DNA. Bars show mean ± S.E.M (*n* = 3–4 biological repeats). Quantities were compared using ordinary one-way ANOVA and Holm Sidak multiple comparisons (***P < 0.001) noting (#) de-regulation of *copA* in the Δ*copY* strain was significantly different to all other GBS strains and conditions.
(TIFF)

S7 Fig The covR-driven transcriptome of GBS in Zn stress. (A) Volcano plots showing data from RNASeq of WT GBS cultures compared to Δ*covR* GBS in THB (Ctrl), or THB supplemented with 0.25 mM Zn (+Zn stress). Transcripts up- or down-regulated in response to Zn (n = 4, >± 2-fold, FDR <0.05) are highlighted in red and blue, respectively. Dotted lines show False discovery rate (FDR; q-value) and fold change cut-offs (± 2-fold). Grey points indicate genes that were unchanged, selected genes are identified individually with black lines. FDR values (*y*-axes) were displayed by -log_10_ (Y) transformation followed by plotting on a log_10_ y-axis to generate volcano plots. (B) Venn comparison showing the number of up-regulated and down-regulated genes shared (Regulated by CovR) or unique to control (CovR-control independent of Zn) or +Zn stress conditions (CovR-control dependent on Zn). Selected genes are listed and the complete list is available as S4 Data.
(TIFF)

S8 Fig Regulation of selected genes by CovR. (A) Expression ratios (log_2_FC) of selected genes, identified by RNAseq as linked to CopY and Zn stress, were compared using qRTPCR as indicated, using RNA isolated from WT or *copY*^-^ GBS grown in THB (-Zn) or THB supplemented with 0.25 mM Zn (+Zn). Five distinct activation states were apparent based on Zn and/or CopY dependency. Fold change values were calculated using *dnaN* as housekeeper and ΔΔ^CT^ values incorporated primer efficiency values. (B) Transcripts of *pcl1*, *ribD*, *ykoI* and *hvgA* were quantified from WT and Δ*covR* GBS grown in THB by qRT-PCR. Fold change values (ratio of *covR-* / WT) were calculated using *dnaN* as housekeeper and ΔΔ^CT^ values incorporated primer efficiency values as previously described [82]. Bars show mean ± S.E.M (*n* = 4 biological repeats). Dotted horizontal lines in A and B indicate ±2 fold change.
(TIFF)

S9 Fig Growth analysis of Δ*hly3* and WT GBS in conditions of Zn stress. WT and Δ*hly3* GBS strains were grown in THB without Zn (A) or THB supplemented with 1.0 mM Zn (B), or (C) CDM without Zn, or (D) CDM supplemented with 0.25 mM Zn. Bars show mean ± S.E.M (*n* = 3 biological repeats) measures of attenuance (*D* at 600_nm_). Strains were compared by Area Under the Curve analysis followed by unpaired *t-*tests for each condition (*** P<0.001).
(TIFF)

S10 Fig Growth analysis of WT GBS, mutants and complemented strains in conditions of Zn stress. WT and isogenic mutants from TraDIS assays were grown in CDM (ctrl; grey) or CDM +0.1 mM Zn as indicated (A) and compared to growth of complemented strains for each mutant. Bars show mean ± S.E.M (*n* = 3 biological repeats) measures of attenuance (*D* at 600_nm_). Strains were compared by Area Under the Curve analysis followed by unpaired *t-*tests for each condition (** P < 0.01, *** P<0.001). WT, and the Δ*arcA* and Δ*celB* mutants, for which complementation plasmids are not available (B), were grown in high Zn stress (CDM +0.25 mM Zn), high Zn stress supplemented with TPEN (CDM +0.25 mM Zn + 0.2 mM TPEN) or control conditions (CDM). Bars show mean ± S.E.M (*n* = 3 biological repeats) measures of attenuance (*D* at 600_nm_).
(TIFF)

S11 Fig Growth of GBS strains in THB + 1.0 mM Zn. WT GBS and Δ*copY*, Δ*celB*, Δ*rfaB* and Δ*stp1* mutants were grown in THB supplemented with 1.0 mM Zn for 12h, to monitor growth in conditions identical to those used for TraDIS analysis. Bars show mean ± S.E.M (*n* = 3 biological repeats) measures of attenuance (*D* at 600_nm_).
(TIFF)

S1 Data The Zn-dependent and *copY*-dependent transcriptomes of GBS. Significantly differentially expressed genes (*n =* 4 replicates; > ± 2-fold difference, Q<0.05) identified by RNA-sequencing analysis of: Δ*copY* GBS grown in THB versus WT GBS grown in THB (Sheet A); Δ*copY* GBS grown in THB + 0.25 mM Zn versus WT GBS grown in THB + 0.25 mM Zn (Sheet B).
(XLSX)

S2 Data Comparative analysis of *copY-*dependent transcriptome data. Lists of the significantly differentially expressed genes identified by RNA-sequencing analysis of Δ*copY* GBS grown in THB versus WT GBS grown in THB or Δ*copY* GBS grown in THB + 0.25 mM Zn versus WT GBS grown in THB + 0.25 mM Zn (A). Genes shared and unique to the Δ*copY* vs WT GBS strain-to-strain comparisons in THB (Ctrl) or THB + 0.25 mM Zn (+Zn stress); separated by up-regulated (B) and down-regulated genes (C) used to construct Venn diagrams in Fig 5.
(XLSX)

S3 Data The *covR*-dependent transcriptomes of GBS. Significantly differentially expressed genes (*n =* 4 replicates; > ± 2-fold difference, Q<0.05) identified by RNA-sequencing analysis of: **Δ***covR* GBS grown in THB versus WT GBS grown in THB (Sheet a); **Δ***covR* GBS grown in THB + 0.25 mM Zn versus WT GBS grown in THB + 0.25 mM Zn (Sheet b). Δ*covR* GBS grown in THB + 0.5 mM Cu versus WT GBS grown in THB + 0.5 mM Cu (Sheet c).
(XLSX)

S4 Data Comparative analysis of *covR-*dependent transcriptome data. Lists of the significantly differentially expressed genes identified by RNA-sequencing analysis of Δ*covR* GBS grown in THB (Ctrl) versus WT GBS grown in THB, or, Δ*covR* GBS grown in THB + 0.25 mM Zn (+Zn stress) versus WT GBS grown in THB + 0.25 mM Zn, or, Δ*covR* GBS grown in THB + 0.25 mM Zn versus Δ*covR* GBS grown in THB (A). Genes shared and unique in the WT and Δ*covR* GBS condition-to-condition comparisons of THB versus THB + 0.25 mM Zn; separated by up-regulated (B) and down-regulated genes (C). Genes shared and unique in the Ctrl vs +Zn stress conditions in strain-to-strain comparisons to dissect the effect of Δ*covR* mutation; separated by up-regulated (D) and down-regulated genes (E). These were used to construct Venn diagrams in S6 Fig.
(XLSX)

S5 Data The Zn resistome of GBS. Genes identified by TraDIS with significant differential IS*S1* insertions (*n =* 3 replicates; FDR <0.001 and P value <0.05) comparing THB containing 1.0 mM Zn to THB without Zn; those with major enrichment or reduction (4 ≤ FC ≤ -4) are highlighted in blue, and moderate enrichment or reduction (2–4 ≤ FC ≤ -2–4) highlighted in green.
(XLSX)

## References

[1] Watly J, Potocki S, Rowinska-Zyrek M. Zinc Homeostasis at the Bacteria/Host Interface-From Coordination Chemistry to Nutritional Immunity. Chemistry. 2016;22(45):15992–6010. Epub 2016/10/22. doi: 10.1002/chem.201602376 . 27555527

[2] Osman D, Cavet JS. Copper homeostasis in bacteria. Adv Appl Microbiol. 2008;65:217–47. Epub 2008/11/26. doi: 10.1016/S0065-2164(08)00608-4 . 19026867

[3] Chandrangsu P, Rensing C, Helmann JD. Metal homeostasis and resistance in bacteria. Nature Reviews Microbiology. 2017;15(6):338–50. Epub 2017/03/28. doi: 10.1038/nrmicro.2017.15 ; PubMed Central PMCID: PMC5963929. 28344348 PMC5963929

[4] German N, Doyscher D, Rensing C. Bacterial killing in macrophages and amoeba: do they all use a brass dagger? Future Microbiol. 2013;8(10):1257–64. Epub 2013/09/26. doi: 10.2217/fmb.13.100 . 24059917

[5] Djoko KY, Ong CL, Walker MJ, McEwan AG. The Role of Copper and Zinc Toxicity in Innate Immune Defense against Bacterial Pathogens. J Biol Chem. 2015;290(31):18954–61. Epub 2015/06/10. doi: 10.1074/jbc.R115.647099 ; PubMed Central PMCID: PMC4521016. 26055706 PMC4521016

[6] Ladomersky E, Petris MJ. Copper tolerance and virulence in bacteria. Metallomics. 2015;7(6):957–64. Epub 2015/02/06. doi: 10.1039/c4mt00327f ; PubMed Central PMCID: PMC4464932. 25652326 PMC4464932

[7] Besold AN, Culbertson EM, Culotta VC. The Yin and Yang of copper during infection. Journal of Biological and Inorganic Chemistry. 2016;21(2):137–44. Epub 2016/01/23. doi: 10.1007/s00775-016-1335-1 ; PubMed Central PMCID: PMC5535265. 26790881 PMC5535265

[8] Djoko KY, Goytia MM, Donnelly PS, Schembri MA, Shafer WM, McEwan AG. Copper(II)-Bis(Thiosemicarbazonato) Complexes as Antibacterial Agents: Insights into Their Mode of Action and Potential as Therapeutics. Antimicrob Agents Chemother. 2015;59(10):6444–53. Epub 2015/08/05. doi: 10.1128/AAC.01289-15 ; PubMed Central PMCID: PMC4576059. 26239980 PMC4576059

[9] McDevitt CA, Ogunniyi AD, Valkov E, Lawrence MC, Kobe B, McEwan AG, et al. A molecular mechanism for bacterial susceptibility to zinc. PLoS Pathog. 2011;7(11):e1002357. Epub 2011/11/11. doi: 10.1371/journal.ppat.1002357 ; PubMed Central PMCID: PMC3207923. 22072971 PMC3207923

[10] Eijkelkamp BA, Morey JR, Ween MP, Ong CL, McEwan AG, Paton JC, et al. Extracellular zinc competitively inhibits manganese uptake and compromises oxidative stress management in *Streptococcus pneumoniae*. PLoS ONE. 2014;9(2):e89427. Epub 2014/02/22. doi: 10.1371/journal.pone.0089427 ; PubMed Central PMCID: PMC3928430. 24558498 PMC3928430

[11] Ong CL, Walker MJ, McEwan AG. Zinc disrupts central carbon metabolism and capsule biosynthesis in *Streptococcus pyogenes*. Scientific Reports. 2015;5:10799. Epub 2015/06/02. doi: 10.1038/srep10799 ; PubMed Central PMCID: PMC4450579. 26028191 PMC4450579

[12] Kapetanovic R, Bokil NJ, Achard ME, Ong CL, Peters KM, Stocks CJ, et al. *Salmonella* employs multiple mechanisms to subvert the TLR-inducible zinc-mediated antimicrobial response of human macrophages. FASEB J. 2016;30(5):1901–12. Epub 2016/02/04. doi: 10.1096/fj.201500061 . 26839376

[13] Achard Maud ES, Stafford Sian L, Bokil Nilesh J, Chartres J, Bernhardt Paul V, Schembri Mark A, et al. Copper redistribution in murine macrophages in response to *Salmonella* infection. Biochem J. 2012;444(1):51–7. doi: 10.1042/BJ20112180 22369063

[14] Hassan KA, Pederick VG, Elbourne LD, Paulsen IT, Paton JC, McDevitt CA, et al. Zinc stress induces copper depletion in *Acinetobacter baumannii*. BMC Microbiol. 2017;17(1):59. Epub 2017/03/13. doi: 10.1186/s12866-017-0965-y ; PubMed Central PMCID: PMC5346208. 28284195 PMC5346208

[15] Ong CL, Gillen CM, Barnett TC, Walker MJ, McEwan AG. An antimicrobial role for zinc in innate immune defense against group A streptococcus. J Infect Dis. 2014;209(10):1500–8. doi: 10.1093/infdis/jiu053 . 24449444

[16] Stewart LJ, Ong CY, Zhang MM, Brouwer S, McIntyre L, Davies MR, et al. Role of Glutathione in Buffering Excess Intracellular Copper in *Streptococcus pyogenes*. mBio. 2020;11(6). Epub 2020/12/03. doi: 10.1128/mBio.02804-20 . 33262259 PMC7733945

[17] Shafeeq S, Yesilkaya H, Kloosterman TG, Narayanan G, Wandel M, Andrew PW, et al. The *cop* operon is required for copper homeostasis and contributes to virulence in *Streptococcus pneumoniae*. Mol Microbiol. 2011;81(5):1255–70. Epub 2011/07/09. doi: 10.1111/j.1365-2958.2011.07758.x . 21736642

[18] O’Brien H, Alvin JW, Menghani SV, Sanchez-Rosario Y, Van Doorslaer K, Johnson MDL. Rules of Expansion: an Updated Consensus Operator Site for the CopR-CopY Family of Bacterial Copper Exporter System Repressors. mSphere. 2020;5(3). Epub 2020/05/29. doi: 10.1128/mSphere.00411-20 ; PubMed Central PMCID: PMC7253601. 32461276 PMC7253601

[19] Kloosterman TG, van der Kooi-Pol MM, Bijlsma JJ, Kuipers OP. The novel transcriptional regulator SczA mediates protection against Zn2+ stress by activation of the Zn2+-resistance gene *czcD* in *Streptococcus pneumoniae*. Mol Microbiol. 2007;65(4):1049–63. Epub 2007/07/21. doi: 10.1111/j.1365-2958.2007.05849.x . 17640279

[20] Sullivan MJ, Goh KGK, Gosling D, Katupitiya L, Ulett GC. Copper Intoxication in Group B Streptococcus Triggers Transcriptional Activation of the cop Operon That Contributes to Enhanced Virulence during Acute Infection. J Bacteriol. 2021;203(19):e0031521. Epub 20210908. doi: 10.1128/JB.00315-21 ; PubMed Central PMCID: PMC8447484. 34251869 PMC8447484

[21] Sullivan MJ, Goh KGK, Ulett GC. Cellular Management of Zinc in Group B Streptococcus Supports Bacterial Resistance against Metal Intoxication and Promotes Disseminated Infection. mSphere. 2021;6(3). Epub 2021/05/21. doi: 10.1128/mSphere.00105-21 . 34011683 PMC8265624

[22] Cobine PA, Jones CE, Dameron CT. Role for zinc(II) in the copper(I) regulated protein CopY. J Inorg Biochem. 2002;88(2):192–6. Epub 2002/01/23. doi: 10.1016/s0162-0134(01)00378-6 . 11803039

[23] Garber ME, Rajeev L, Kazakov AE, Trinh J, Masuno D, Thompson MG, et al. Multiple signaling systems target a core set of transition metal homeostasis genes using similar binding motifs. Mol Microbiol. 2018;107(6):704–17. Epub 2018/01/18. doi: 10.1111/mmi.13909 . 29341298

[24] Culbertson EM, Bruno VM, Cormack BP, Culotta VC. Expanded role of the Cu-sensing transcription factor Mac1p in *Candida albicans*. Mol Microbiol. 2020;114(6):1006–18. Epub 2020/08/19. doi: 10.1111/mmi.14591 . 32808698 PMC7856572

[25] Thomas L, Cook L. Two-Component Signal Transduction Systems in the Human Pathogen *Streptococcus agalactiae*. Infect Immun. 2020;88(7). Epub 2020/01/29. doi: 10.1128/IAI.00931-19 ; PubMed Central PMCID: PMC7309623. 31988177 PMC7309623

[26] Mazzuoli MV, Daunesse M, Varet H, Rosinski-Chupin I, Legendre R, Sismeiro O, et al. The CovR regulatory network drives the evolution of Group B Streptococcus virulence. PLoS Genet. 2021;17(9):e1009761. Epub 2021/09/08. doi: 10.1371/journal.pgen.1009761 ; PubMed Central PMCID: PMC8448333. 34491998 PMC8448333

[27] Nakata M, Kreikemeyer B. Genetics, Structure, and Function of Group A Streptococcal Pili. Frontiers in Microbiology. 2021;12:616508. Epub 2021/02/27. doi: 10.3389/fmicb.2021.616508 ; PubMed Central PMCID: PMC7900414. 33633705 PMC7900414

[28] Lamy MC, Zouine M, Fert J, Vergassola M, Couve E, Pellegrini E, et al. CovS/CovR of group B streptococcus: a two-component global regulatory system involved in virulence. Mol Microbiol. 2004;54(5):1250–68. doi: 10.1111/j.1365-2958.2004.04365.x . 15554966

[29] Lembo A, Gurney MA, Burnside K, Banerjee A, de los Reyes M, Connelly JE, et al. Regulation of CovR expression in Group B Streptococcus impacts blood-brain barrier penetration. Mol Microbiol. 2010;77(2):431–43. Epub 2010/05/26. doi: 10.1111/j.1365-2958.2010.07215.x ; PubMed Central PMCID: PMC2909351. 20497331 PMC2909351

[30] Patras KA, Wang NY, Fletcher EM, Cavaco CK, Jimenez A, Garg M, et al. Group B Streptococcus CovR regulation modulates host immune signalling pathways to promote vaginal colonization. Cell Microbiol. 2013;15(7):1154–67. Epub 2013/01/10. doi: 10.1111/cmi.12105 ; PubMed Central PMCID: PMC3657335. 23298320 PMC3657335

[31] Sullivan MJ, Leclercq SY, Ipe DS, Carey AJ, Smith JP, Voller N, et al. Effect of the *Streptococcus agalactiae* Virulence Regulator CovR on the Pathogenesis of Urinary Tract Infection. J Infect Dis. 2017;215(3):475–83. doi: 10.1093/infdis/jiw589 . 28011914 PMC6455028

[32] Gryllos I, Grifantini R, Colaprico A, Jiang S, Deforce E, Hakansson A, et al. Mg(2+) signalling defines the group A streptococcal CsrRS (CovRS) regulon. Mol Microbiol. 2007;65(3):671–83. Epub 2007/07/05. doi: 10.1111/j.1365-2958.2007.05818.x . 17608796

[33] Rosinski-Chupin I, Sauvage E, Fouet A, Poyart C, Glaser P. Conserved and specific features of *Streptococcus pyogenes* and *Streptococcus agalactiae* transcriptional landscapes. BMC Genomics. 2019;20(1):236. Epub 2019/03/25. doi: 10.1186/s12864-019-5613-5 ; PubMed Central PMCID: PMC6431027. 30902048 PMC6431027

[34] Jiang SM, Cieslewicz MJ, Kasper DL, Wessels MR. Regulation of virulence by a two-component system in group B streptococcus. J Bacteriol. 2005;187(3):1105–13. Epub 2005/01/22. doi: 10.1128/JB.187.3.1105-1113.2005 ; PubMed Central PMCID: PMC545708. 15659687 PMC545708

[35] Jiang SM, Ishmael N, Dunning Hotopp J, Puliti M, Tissi L, Kumar N, et al. Variation in the group B Streptococcus CsrRS regulon and effects on pathogenicity. J Bacteriol. 2008;190(6):1956–65. Epub 2008/01/22. doi: 10.1128/JB.01677-07 ; PubMed Central PMCID: PMC2258897. 18203834 PMC2258897

[36] Moulin P, Patron K, Cano C, Zorgani MA, Camiade E, Borezee-Durant E, et al. The Adc/Lmb System Mediates Zinc Acquisition in *Streptococcus agalactiae* and Contributes to Bacterial Growth and Survival. J Bacteriol. 2016;198(24):3265–77. Epub 2016/09/28. doi: 10.1128/JB.00614-16 ; PubMed Central PMCID: PMC5116930. 27672194 PMC5116930

[37] Maguin E, Prevost H, Ehrlich SD, Gruss A. Efficient insertional mutagenesis in lactococci and other gram-positive bacteria. J Bacteriol. 1996;178(3):931–5. Epub 1996/02/01. doi: 10.1128/jb.178.3.931-935.1996 ; PubMed Central PMCID: PMC177749. 8550537 PMC177749

[38] Stoyanov JV, Hobman JL, Brown NL. CueR (YbbI) of *Escherichia coli* is a MerR family regulator controlling expression of the copper exporter CopA. Mol Microbiol. 2001;39(2):502–11. Epub 2001/01/03. doi: 10.1046/j.1365-2958.2001.02264.x . 11136469

[39] Spencer BL, Deng L, Patras KA, Burcham ZM, Sanches GF, Nagao PE, et al. Cas9 Contributes to Group B Streptococcal Colonization and Disease. Frontiers in Microbiology. 2019;10:1930. Epub 2019/09/10. doi: 10.3389/fmicb.2019.01930 ; PubMed Central PMCID: PMC6712506. 31497003 PMC6712506

[40] Deng L, Mu R, Weston TA, Spencer BL, Liles RP, Doran KS. Characterization of a Two-Component System Transcriptional Regulator, LtdR, That Impacts Group B Streptococcal Colonization and Disease. Infect Immun. 2018;86(7). Epub 2018/04/25. doi: 10.1128/IAI.00822-17 ; PubMed Central PMCID: PMC6013667. 29685987 PMC6013667

[41] Tarrant E G PR, McIlvin MR, Stevenson J, Barwinska-Sendra A, Stewart LJ, et al. Copper stress in *Staphylococcus aureus* leads to adaptive changes in central carbon metabolism. Metallomics. 2019;11(1):183–200. Epub 2018/11/18. doi: 10.1039/c8mt00239h ; PubMed Central PMCID: PMC6350627. 30443649 PMC6350627

[42] Baker J, Sitthisak S, Sengupta M, Johnson M, Jayaswal RK, Morrissey JA. Copper stress induces a global stress response in *Staphylococcus aureus* and represses *sae* and *agr* expression and biofilm formation. Appl Environ Microbiol. 2010;76(1):150–60. Epub 2009/11/03. doi: 10.1128/AEM.02268-09 ; PubMed Central PMCID: PMC2798663. 19880638 PMC2798663

[43] Abbas CA, Sibirny AA. Genetic control of biosynthesis and transport of riboflavin and flavin nucleotides and construction of robust biotechnological producers. Microbiol Mol Biol Rev. 2011;75(2):321–60. Epub 2011/06/08. doi: 10.1128/MMBR.00030-10 ; PubMed Central PMCID: PMC3122625. 21646432 PMC3122625

[44] Mironov AS, Gusarov I, Rafikov R, Lopez LE, Shatalin K, Kreneva RA, et al. Sensing small molecules by nascent RNA: a mechanism to control transcription in bacteria. Cell. 2002;111(5):747–56. Epub 2002/12/05. doi: 10.1016/s0092-8674(02)01134-0 . 12464185

[45] Winkler WC, Cohen-Chalamish S, Breaker RR. An mRNA structure that controls gene expression by binding FMN. Proc Natl Acad Sci U S A. 2002;99(25):15908–13. Epub 2002/11/29. doi: 10.1073/pnas.212628899 ; PubMed Central PMCID: PMC138538. 12456892 PMC138538

[46] Ong CY, Berking O, Walker MJ, McEwan AG. New Insights into the Role of Zinc Acquisition and Zinc Tolerance in Group A Streptococcal Infection. Infect Immun. 2018;86(6). Epub 2018/03/28. doi: 10.1128/IAI.00048-18 ; PubMed Central PMCID: PMC5964520. 29581188 PMC5964520

[47] Vitreschak AG, Rodionov DA, Mironov AA, Gelfand MS. Regulation of riboflavin biosynthesis and transport genes in bacteria by transcriptional and translational attenuation. Nucleic Acids Res. 2002;30(14):3141–51. Epub 2002/07/24. doi: 10.1093/nar/gkf433 ; PubMed Central PMCID: PMC135753. 12136096 PMC135753

[48] Hartmann N, McMurtrey C, Sorensen ML, Huber ME, Kurapova R, Coleman FT, et al. Riboflavin Metabolism Variation among Clinical Isolates of *Streptococcus pneumoniae* Results in Differential Activation of Mucosal-associated Invariant T Cells. Am J Respir Cell Mol Biol. 2018;58(6):767–76. Epub 2018/01/23. doi: 10.1165/rcmb.2017-0290OC ; PubMed Central PMCID: PMC6002660. 29356555 PMC6002660

[49] Stocks CJ, Phan MD, Achard MES, Nhu NTK, Condon ND, Gawthorne JA, et al. Uropathogenic *Escherichia coli* employs both evasion and resistance to subvert innate immune-mediated zinc toxicity for dissemination. Proc Natl Acad Sci U S A. 2019;116(13):6341–50. Epub 2019/03/09. doi: 10.1073/pnas.1820870116 ; PubMed Central PMCID: PMC6442554. 30846555 PMC6442554

[50] Burcham LR, Le Breton Y, Radin JN, Spencer BL, Deng L, Hiron A, et al. Identification of Zinc-Dependent Mechanisms Used by Group B *Streptococcus* To Overcome Calprotectin-Mediated Stress. mBio. 2020;11(6). Epub 2020/11/12. doi: 10.1128/mBio.02302-20 ; PubMed Central PMCID: PMC7667036. 33173000 PMC7667036

[51] Portmann R, Poulsen KR, Wimmer R, Solioz M. CopY-like copper inducible repressors are putative ’winged helix’ proteins. Biometals. 2006;19(1):61–70. Epub 2006/02/28. doi: 10.1007/s10534-005-5381-3 . 16502332

[52] Magnani D, Barre O, Gerber SD, Solioz M. Characterization of the CopR regulon of *Lactococcus lactis* IL1403. J Bacteriol. 2008;190(2):536–45. Epub 2007/11/13. doi: 10.1128/JB.01481-07 ; PubMed Central PMCID: PMC2223693. 17993525 PMC2223693

[53] Garcia-Castellanos R, Mallorqui-Fernandez G, Marrero A, Potempa J, Coll M, Gomis-Ruth FX. On the transcriptional regulation of methicillin resistance: MecI repressor in complex with its operator. J Biol Chem. 2004;279(17):17888–96. Epub 2004/02/13. doi: 10.1074/jbc.M313123200 . 14960592

[54] Choe D, Szubin R, Dahesh S, Cho S, Nizet V, Palsson B, et al. Genome-scale analysis of Methicillin-resistant *Staphylococcus aureus* USA300 reveals a tradeoff between pathogenesis and drug resistance. Scientific Reports. 2018;8(1):2215. Epub 2018/02/06. doi: 10.1038/s41598-018-20661-1 ; PubMed Central PMCID: PMC5797083. 29396540 PMC5797083

[55] Neubert MJ, Dahlmann EA, Ambrose A, Johnson MDL. Copper Chaperone CupA and Zinc Control CopY Regulation of the Pneumococcal cop Operon. mSphere. 2017;2(5). Epub 2017/10/25. doi: 10.1128/mSphere.00372-17 ; PubMed Central PMCID: PMC5646241. 29062896 PMC5646241

[56] Glauninger H, Zhang Y, Higgins KA, Jacobs AD, Martin JE, Fu Y, et al. Metal-dependent allosteric activation and inhibition on the same molecular scaffold: the copper sensor CopY from *Streptococcus pneumoniae*. Chemical Science. 2018;9(1):105–18. Epub 2018/02/06. doi: 10.1039/c7sc04396a ; PubMed Central PMCID: PMC5772342. 29399317 PMC5772342

[57] Capdevila DA, Wang J, Giedroc DP. Bacterial Strategies to Maintain Zinc Metallostasis at the Host-Pathogen Interface. J Biol Chem. 2016;291(40):20858–68. doi: 10.1074/jbc.R116.742023 27462080 PMC5076499

[58] Goh KGK, Sullivan MJ, Ulett GC. The Copper Resistome of Group B Streptococcus Reveals Insight into the Genetic Basis of Cellular Survival during Metal Ion Stress. J Bacteriol. 2022;204(5):e0006822. Epub 20220411. doi: 10.1128/jb.00068-22 ; PubMed Central PMCID: PMC9112871. 35404113 PMC9112871

[59] Zhu L, Yerramilli P, Pruitt L, Saavedra MO, Cantu CC, Olsen RJ, et al. Genome-wide assessment of *Streptococcus agalactiae* genes required for fitness in human whole blood and plasma. Infect Immun. 2020. Epub 2020/08/05. doi: 10.1128/IAI.00357-20 . 32747604 PMC7504961

[60] Hooven TA, Catomeris AJ, Bonakdar M, Tallon LJ, Santana-Cruz I, Ott S, et al. The *Streptococcus agalactiae* Stringent Response Enhances Virulence and Persistence in Human Blood. Infect Immun. 2018;86(1). Epub 2017/11/08. doi: 10.1128/IAI.00612-17 ; PubMed Central PMCID: PMC5736797. 29109175 PMC5736797

[61] Hoeger J, Simon TP, Doemming S, Thiele C, Marx G, Schuerholz T, et al. Alterations in zinc binding capacity, free zinc levels and total serum zinc in a porcine model of sepsis. Biometals. 2015;28(4):693–700. Epub 2015/05/06. doi: 10.1007/s10534-015-9858-4 . 25940830

[62] Kushner I. The phenomenon of the acute phase response. Ann N Y Acad Sci. 1982;389:39–48. Epub 1982/01/01. doi: 10.1111/j.1749-6632.1982.tb22124.x . 7046585

[63] Eijkelkamp BA, Morey JR, Neville SL, Tan A, Pederick VG, Cole N, et al. Dietary zinc and the control of *Streptococcus pneumoniae* infection. PLoS Pathog. 2019;15(8):e1007957. Epub 2019/08/23. doi: 10.1371/journal.ppat.1007957 ; PubMed Central PMCID: PMC6705770. 31437249 PMC6705770

[64] Bayle L, Chimalapati S, Schoehn G, Brown J, Vernet T, Durmort C. Zinc uptake by *Streptococcus pneumoniae* depends on both AdcA and AdcAII and is essential for normal bacterial morphology and virulence. Mol Microbiol. 2011;82(4):904–16. Epub 2011/10/26. doi: 10.1111/j.1365-2958.2011.07862.x . 22023106

[65] Burnside K, Lembo A, Harrell MI, Gurney M, Xue L, BinhTran NT, et al. Serine/threonine phosphatase Stp1 mediates post-transcriptional regulation of hemolysin, autolysis, and virulence of group B Streptococcus. J Biol Chem. 2011;286(51):44197–210. Epub 2011/11/15. doi: 10.1074/jbc.M111.313486 ; PubMed Central PMCID: PMC3243546. 22081606 PMC3243546

[66] Martin JE, Lisher JP, Winkler ME, Giedroc DP. Perturbation of manganese metabolism disrupts cell division in *Streptococcus pneumoniae*. Mol Microbiol. 2017;104(2):334–48. Epub 2017/01/28. doi: 10.1111/mmi.13630 ; PubMed Central PMCID: PMC5380469. 28127804 PMC5380469

[67] Rued BE, Zheng JJ, Mura A, Tsui HT, Boersma MJ, Mazny JL, et al. Suppression and synthetic-lethal genetic relationships of DeltagpsB mutations indicate that GpsB mediates protein phosphorylation and penicillin-binding protein interactions in *Streptococcus pneumoniae* D39. Mol Microbiol. 2017;103(6):931–57. Epub 2016/12/24. doi: 10.1111/mmi.13613 ; PubMed Central PMCID: PMC5344783. 28010038 PMC5344783

[68] Rajagopal L, Clancy A, Rubens CE. A eukaryotic type serine/threonine kinase and phosphatase in *Streptococcus agalactiae* reversibly phosphorylate an inorganic pyrophosphatase and affect growth, cell segregation, and virulence. J Biol Chem. 2003;278(16):14429–41. Epub 2003/02/04. doi: 10.1074/jbc.M212747200 . 12562757

[69] Ipe DS, Sullivan MJ, Goh KGK, Hashimi SM, Munn AL, Ulett GC. Conserved bacterial *de novo* guanine biosynthesis pathway enables microbial survival and colonization in the environmental niche of the urinary tract. The ISME Journal. 2021;15(7):2158–62. Epub 2021/03/03. doi: 10.1038/s41396-021-00934-w ; PubMed Central PMCID: PMC8245529. 33649549 PMC8245529

[70] Park DM, Overton KW, Liou MJ, Jiao Y. Identification of a U/Zn/Cu responsive global regulatory two-component system in *Caulobacter crescentus*. Mol Microbiol. 2017;104(1):46–64. Epub 2016/12/31. doi: 10.1111/mmi.13615 . 28035693

[71] Raimunda D, Long JE, Padilla-Benavides T, Sassetti CM, Arguello JM. Differential roles for the Co(2+) /Ni(2+) transporting ATPases, CtpD and CtpJ, in *Mycobacterium tuberculosis* virulence. Mol Microbiol. 2014;91(1):185–97. Epub 2013/11/22. doi: 10.1111/mmi.12454 ; PubMed Central PMCID: PMC3885230. 24255990 PMC3885230

[72] Burcham LR, Hill RA, Caulkins RC, Emerson JP, Nanduri B, Rosch JW, et al. *Streptococcus pneumoniae* metal homeostasis alters cellular metabolism. Metallomics. 2020;12(9):1416–27. Epub 2020/07/18. doi: 10.1039/d0mt00118j ; PubMed Central PMCID: PMC7530088. 32676626 PMC7530088

[73] White C, Lee J, Kambe T, Fritsche K, Petris MJ. A role for the ATP7A copper-transporting ATPase in macrophage bactericidal activity. J Biol Chem. 2009;284(49):33949–56. Epub 2009/10/08. doi: 10.1074/jbc.M109.070201 ; PubMed Central PMCID: PMC2797165. 19808669 PMC2797165

[74] Sullivan MJ, Forde BM, Prince DW, Ipe DS, Ben Zakour NL, Davies MR, et al. Complete Genome Sequence of Serotype III *Streptococcus agalactiae* Sequence Type 17 Strain 874391. Genome Announcements. 2017;5(42). Epub 2017/10/21. doi: 10.1128/genomeA.01107-17 . 29051249 PMC5646402

[75] Sullivan MJ, Ulett GC. Stable Expression of Modified Green Fluorescent Protein in Group B Streptococci To Enable Visualization in Experimental Systems. Appl Environ Microbiol. 2018;84(18). Epub 2018/07/15. doi: 10.1128/AEM.01262-18 . 30006391 PMC6121993

[76] Ipe DS, Ben Zakour NL, Sullivan MJ, Beatson SA, Ulett KB, Benjamin WHJ, et al. Discovery and Characterization of Human-Urine Utilization by Asymptomatic-Bacteriuria-Causing *Streptococcus agalactiae*. Infect Immun. 2015;84(1):307–19. doi: 10.1128/IAI.00938-15 ; PubMed Central PMCID: PMC4694007. 26553467 PMC4694007

[77] Dunny GM, Lee LN, LeBlanc DJ. Improved electroporation and cloning vector system for gram-positive bacteria. Appl Environ Microbiol. 1991;57(4):1194–201. Epub 1991/04/01. PubMed Central PMCID: PMC182867. doi: 10.1128/aem.57.4.1194-1201.1991 1905518 PMC182867

[78] LeBlanc DJ, Lee LN, Abu-Al-Jaibat A. Molecular, genetic, and functional analysis of the basic replicon of pVA380-1, a plasmid of oral streptococcal origin. Plasmid. 1992;28(2):130–45. Epub 1992/09/01. doi: 10.1016/0147-619x(92)90044-b . 1409970

[79] Untergasser A, Cutcutache I, Koressaar T, Ye J, Faircloth BC, Remm M, et al. Primer3—new capabilities and interfaces. Nucleic Acids Res. 2012;40(15):e115. Epub 2012/06/26. doi: 10.1093/nar/gks596 ; PubMed Central PMCID: PMC3424584. 22730293 PMC3424584

[80] Untergasser A, Nijveen H, Rao X, Bisseling T, Geurts R, Leunissen JA. Primer3Plus, an enhanced web interface to Primer3. Nucleic Acids Res. 2007;35(Web Server issue):W71–4. doi: 10.1093/nar/gkm306 ; PubMed Central PMCID: PMC1933133. 17485472 PMC1933133

[81] Bustin SA, Benes V, Garson JA, Hellemans J, Huggett J, Kubista M, et al. The MIQE guidelines: minimum information for publication of quantitative real-time PCR experiments. Clin Chem. 2009;55(4):611–22. doi: 10.1373/clinchem.2008.112797 . 19246619

[82] Pfaffl MW. A new mathematical model for relative quantification in real-time RT-PCR. Nucleic Acids Res. 2001;29(9):e45. Epub 2001/05/09. doi: 10.1093/nar/29.9.e45 ; PubMed Central PMCID: PMC55695. 11328886 PMC55695

[83] Acharya D, Sullivan MJ, Duell BL, Goh KGK, Katupitiya L, Gosling D, et al. Rapid Bladder Interleukin-10 Synthesis in Response to Uropathogenic *Escherichia coli* Is Part of a Defense Strategy Triggered by the Major Bacterial Flagellar Filament FliC and Contingent on TLR5. mSphere. 2019;4(6). Epub 2019/11/30. doi: 10.1128/mSphere.00545-19 ; PubMed Central PMCID: PMC6881718. 31776239 PMC6881718

[84] Leclercq SY, Sullivan MJ, Ipe DS, Smith JP, Cripps AW, Ulett GC. Pathogenesis of *Streptococcus* urinary tract infection depends on bacterial strain and beta-hemolysin/cytolysin that mediates cytotoxicity, cytokine synthesis, inflammation and virulence. Scientific Reports. 2016;6:29000. doi: 10.1038/srep29000 ; PubMed Central PMCID: PMC4935997. 27383371 PMC4935997

[85] Valdes Lopez JF, Urcuqui-Inchima S. Synergism between phorbol-12-myristate-13-acetate and vitamin D3 in the differentiation of U937 cells to monocytes and macrophages. Morphologie. 2018;102(338):205–18. Epub 2018/08/05. doi: 10.1016/j.morpho.2018.06.001 . 30075941

[86] Anders S, Pyl PT, Huber W. HTSeq—a Python framework to work with high-throughput sequencing data. Bioinformatics. 2015;31(2):166–9. Epub 2014/09/28. doi: 10.1093/bioinformatics/btu638 ; PubMed Central PMCID: PMC4287950. 25260700 PMC4287950

[87] Love MI, Huber W, Anders S. Moderated estimation of fold change and dispersion for RNA-seq data with DESeq2. Genome Biol. 2014;15(12):550. Epub 2014/12/18. doi: 10.1186/s13059-014-0550-8 ; PubMed Central PMCID: PMC4302049. 25516281 PMC4302049

[88] Sullivan MJ, Ulett GC. Evaluation of hematogenous spread and ascending infection in the pathogenesis of acute pyelonephritis due to group B streptococcus in mice. Microb Pathog. 2020;138:103796. Epub 2019/10/16. doi: 10.1016/j.micpath.2019.103796 . 31614193

[89] Charbonneau ARL, Forman OP, Cain AK, Newland G, Robinson C, Boursnell M, et al. Defining the ABC of gene essentiality in streptococci. BMC Genomics. 2017;18(1):426. Epub 2017/06/02. doi: 10.1186/s12864-017-3794-3 ; PubMed Central PMCID: PMC5452409. 28569133 PMC5452409

[90] Barquist L, Mayho M, Cummins C, Cain AK, Boinett CJ, Page AJ, et al. The TraDIS toolkit: sequencing and analysis for dense transposon mutant libraries. Bioinformatics. 2016;32(7):1109–11. Epub 2016/01/23. doi: 10.1093/bioinformatics/btw022 ; PubMed Central PMCID: PMC4896371. 26794317 PMC4896371

[91] Page AJ, Bastkowski S, Yasir M, Turner AK, Le Viet T, Savva GM, et al. AlbaTraDIS: Comparative analysis of large datasets from parallel transposon mutagenesis experiments. PLoS Comput Biol. 2020;16(7):e1007980. Epub 2020/07/18. doi: 10.1371/journal.pcbi.1007980 . 32678849 PMC7390408

[92] Rajagopal L, Vo A, Silvestroni A, Rubens CE. Regulation of purine biosynthesis by a eukaryotic-type kinase in *Streptococcus agalactiae*. Mol Microbiol. 2005;56(5):1329–46. doi: 10.1111/j.1365-2958.2005.04620.x ; PubMed Central PMCID: PMC2366208. 15882424 PMC2366208

[93] Rajagopal L, Vo A, Silvestroni A, Rubens CE. Regulation of cytotoxin expression by converging eukaryotic-type and two-component signalling mechanisms in *Streptococcus agalactiae*. Mol Microbiol. 2006;62(4):941–57. Epub 2006/09/29. doi: 10.1111/j.1365-2958.2006.05431.x ; PubMed Central PMCID: PMC2593684. 17005013 PMC2593684

