# Supplementary material for: Regulatory cross-talk supports resistance to Zn intoxication in Streptococcus
Source: PLoS Pathog. 2022 Jul 21;18(7):e1010607. doi: 10.1371/journal.ppat.1010607 (PMC9345489; doi:10.1371/journal.ppat.1010607)
Supplement: S1 Table — (DOCX) [file ppat.1010607.s001.docx]

**Supplementary Table S1**. Modified Defined Medium (MDM) components and recipe

| **A - Inorganics** | | | **F - Vitamins, Water soluble I** | | |
| --- | --- | --- | --- | --- | --- |
| **Chemical** | **Per L:** | **Solvent** | **Chemical** | **Per L:** | **Solvent** |
| CaCl_2_ | 0.2 g | H_2_O | D-Biotin | 1 mg | H_2_O |
| KCl | 0.4 g | H_2_O | Choline chloride | 5 mg | H_2_O |
| NaCl | 6.4 g | H_2_O | Myo-inositol | 9.2 mg | H_2_O |
| NaH_2_PO_4_ | 0.109 g | H_2_O | Niacinamide | 5 mg | H_2_O |
| MgSO_4_ | 97.67 mg | H_2_O | D-Pantothenic Acid • 1⁄2Ca | 5 mg | H_2_O |
| **B - Amino Acids, Water soluble I** | | | Pyridoxal • HCl | 5 mg | H_2_O |
| L-Arginine.HCl | 84 mg | H_2_O | Thiamine • HCl | 5 mg | H_2_O |
| L-Alanine | 100 mg | H_2_O | **G - Vitamins, Alkali soluble (NaOH)** | | |
| Glycine | 30 mg | H_2_O | Folic Acid | 5 mg | 1M NaOH |
| L-Histidine • HCl • H_2_O | 42 mg | H_2_O | Riboflavin | 0.5 mg | 0.1M NaOH |
| L-Isoleucine | 105 mg | H_2_O | **H - Other** | | |
| L-Leucine | 105 mg | H_2_O | α-lipoic acid | 2.5 mg | Ethanol |
| L-Lysine • HCl | 146 mg | H_2_O | Fe(NO_3_)_3_ • 9H2O | 0.1 mg | H_2_O |
| **C - Amino Acids, Water soluble II** | | | NaHCO_3_ | 3.7 g | H_2_O |
| L-cysteine | 50 mg | H_2_O | **I - Carbon source** | | |
| L-Phenylalanine | 66 mg | H_2_O | Sodium pyruvate | 0.11 g | H_2_O |
| L-proline | 100 mg | H_2_O | Glucose | 1.0 g | H_2_O |
| L-Serine | 42 mg | H_2_O | MDM was prepared as follows, using MilliQ H_2_O:  Solution A- 10 x solution dissolved in 1L H_2_O  Solution B- 10 x solution dissolved in 0.1 L H_2_O*  Solution C- 10 x solution dissolved in 0.1 L H_2_O*  Solution D- 10 x solution dissolved in 0.1 L 1M HCl  Solution E- 10 x solution dissolved in 20mL 1M NaOH  Solution F- 10 x solution dissolved in 10mL H_2_O  Folic Acid 50 mg dissolved in 5 mL 1M NaOH, combined with Solution F (15 mL final).  H – dissolved 25 mg α-lipoic acid in 1mL 100% ethanol  Dissolved 20 mg Fe(NO_3_)_3_ • 9H2O in 10 mL H_2_O  Solution I- 10 x solution dissolved in 50mL H_2_O  * to assist dissolving, NaOH was added drop-wise  **To make 1L of MDM**:  -To ~800 mL of MilliQ H_2_O combine 100 mL Solution A; 10 mL of Solutions B, C, D, E; 2 mL of Solution E; 1.5 mL of Solution F; 5 mL of Solution I, 50 uL of Fe(NO_3_)_3_ • 9H2O solution, 100 uL α-lipoic acid in ethanol.  - Add 3.7 g NaHCO_3_  - Adjust pH to 7.4, then add H_2_O to final volume of 1L. Filter sterilise and store at 4ºC | | |
| L-Threonine | 95 mg | H_2_O |  |  |  |
| L-Tyrosine • 2Na • 2H_2_O | 103.79 mg | H_2_O |  |  |  |
| L-Valine | 94 mg | H_2_O |  |  |  |
| **D - Amino Acids, Acid(HCl) soluble** | | |  |  |  |
| L-aspartic acid | 150 mg | 1M HCl |  |  |  |
| L-asparagine | 100 mg | 1M HCl |  |  |  |
| L-Cystine.2HCl | 62.6 mg | 1M HCl |  |  |  |
| L-glutamic acid | 150 mg | 1M HCl |  |  |  |
| L-Glutamine | 100 mg | 1M HCl |  |  |  |
| L-Methionine | 30 mg | 1M HCl |  |  |  |
| L-Tryptophan | 166 mg | 1M HCl |  |  |  |
| **E - Nucleotides** | | |  |  |  |
| Uracil | 10 mg | 1M NaOH |  |  |  |
| Adenine | 10 mg | 1M NaOH |  |  |  |
| Guanine | 10 mg | 1M NaOH |  |  |  |
| Xanthine | 10 mg | 1M NaOH |  |  |  |
