# Supplementary material for: Regulatory cross-talk supports resistance to Zn intoxication in Streptococcus
Source: PLoS Pathog. 2022 Jul 21;18(7):e1010607. doi: 10.1371/journal.ppat.1010607 (PMC9345489; doi:10.1371/journal.ppat.1010607)
Supplement: S2 Table — (DOCX) [file ppat.1010607.s002.docx]

Supplementary Table S2. Bacterial strains and plasmids used in this study.

| **Bacteria** | | |
| --- | --- | --- |
| **Strains** | **Characteristics*** | **Source** |
| *E. coli* DH5α | *huA2 lac(*Δ*)U169 phoA glnV44 Φ80' lacZ(*Δ*)M15 gyrA96 recA1 relA1 endA1 thi-1 hsdR17* | Bethesda Research Labs |
| *S. agalactiae* 874391 | Wild type, Sequence type-17, Serotype III strain | [1] |
| *S. agalactiae* GU2400 | 874391Δ*covR* (*covR*^-^ mutant); Cm  Locus tag: CHF17_RS08405 | [2] |
| *S. agalactiae* GU2791 | 874391Δ*sczA* (*sczA*^-^ mutant)  Locus tag: CHF17_RS02860 | [3] |
| *S. agalactiae* GU2857 | 874391Δ*copY* (*copY*^-^ mutant)  Locus tag: CHF17_RS02565 | [4] |
| *S. agalactiae* GU2978 | 874391Δ*hly3* (*hly3*^-^ mutant)  Locus tag: CHF17_RS06900 | This work |
| *S. agalactiae* GU2991 | 874391Δ*celB* (*celB*^-^ mutant)  Locus tag: CHF17_RS08095 | This work |
| *S. agalactiae* GU3021 | 874391Δ*rfaB* (*rfaB*^-^ mutant)  Locus tag: CHF17_RS04275 | [5] |
| *S. agalactiae* GU3039 | 874391Δ*ribD* (*ribD*^-^ mutant)  Locus tag: CHF17_RS04465 | [5] |
| *S. agalactiae* GU3041 | 874391Δ*arcR* (*arcR*^-^ mutant)  Locus tag: CHF17_RS11035 | This work |
| *S. agalactiae* GU3050 | 874391Δ*plyB* (*plyB*^-^ mutant)  Locus tag: CHF17_RS04510 | [5] |
| *S. agalactiae* GU3055 | 874391Δ*stp1* (*stp1*^-^ mutant)  Locus tag: CHF17_RS02200 | [5] |
| *S. agalactiae* GU3063 | 874391Δ*stk1* (*stk1*^-^ mutant)  Locus tag: CHF17_RS02205 | This work |
| *S. agalactiae* GU3075 | 874391Δ*yceG* (*yceG*^-^ mutant)  Locus tag: CHF17_RS08350 | [5] |
| *S. agalactiae* GU3123 | *copY*-complement (pGU3112) in GU2857, Sp | This work |
| *S. agalactiae* GU3126 | 874391Δ*stp1* pGU3119 (pDL278::*stp1*/*stk1*) | [5] |
| *S. agalactiae* GU3151 | 874391Δ*hisMJP* pGU3130 (pDL278::*hisMJP*) | [5] |
| *S. agalactiae* GU3153 | 874391Δ*plyB* pGU3104 (pDL278::*plyB*) | [5] |
| *S. agalactiae* GU3165 | 874391Δ*rfaB* pGU3118 (pDL278::*rfaB*) | [5] |
| *S. agalactiae* GU3154 | 874391Δ*yceG* pGU3134 (pDL278::*yceG*) | [5] |
| *S. agalactiae* GU3123 | 874391Δ*copY* pGU3112 *(*pDL278::*copYAZ*) | This work |
| *S. agalactiae* GU2894 | 874391Δ*sczA* pGU2861 (pDL278::*sczA*) | [3] |
| *S. agalactiae* GU2579 | 874391Δ*covR*::*covR* (Marker-rescue complemented *covR*- strain) | [2] |
| **Plasmids** | | |
| pHY304*aad9* | *ori* (Ts); temperature-sensitive shuttle vector; Sp | [6] |
| pDL278 | *E. coli Streptococcus* shuttle vector; Sp | [7] |
| pGh9-IS*S1* | *ori* (Ts); temperature-sensitive IS*S1* mutagenesis vector; Erm | [8] |
| pGU2878 | pHY304*aad9-*derivative Δ*celB* construct; Sp | This work |
| pGU3016 | pHY304*aad9-*derivative Δ*stk1* construct; Sp | This work |
| pGU2956 | pHY304*aad9-*derivative Δ*arcR* construct; Sp | This work |
| pGU2969 | pHY304*aad9-*derivative Δ*hly3* construct; Sp | This work |
| pGU3112 | pDL278 + *copYAZ* complement construct, Sp | [4] |
| pGU2861 | pDL278 + *sczA* complement construct, Sp | [3] |

* Sp = Spectinomycin-resistant; Cm = Chloramphenicol-resistant; Ts = temperature-sensitive; Erm = Erythromycin-resistant

**References**

1. Takahashi S, Nagano Y, Nagano N, Hayashi O, Taguchi F, Okuwaki Y. Role of C5a-ase in group B streptococcal resistance to opsonophagocytic killing. Infect Immun. 1995;63(12):4764-9. PubMed PMID: 7591133; PubMed Central PMCID: PMCPMC173682.

2. Sullivan MJ, Leclercq SY, Ipe DS, Carey AJ, Smith JP, Voller N, et al. Effect of the *Streptococcus agalactiae* Virulence Regulator CovR on the Pathogenesis of Urinary Tract Infection. J Infect Dis. 2017;215(3):475-83. doi: 10.1093/infdis/jiw589. PubMed PMID: 28011914.

3. Sullivan MJ, Goh KGK, Ulett GC. Cellular Management of Zinc in Group B Streptococcus Supports Bacterial Resistance against Metal Intoxication and Promotes Disseminated Infection. mSphere. 2021;6(3). Epub 2021/05/21. doi: 10.1128/mSphere.00105-21. PubMed PMID: 34011683.

4. Sullivan MJ, Goh KGK, Gosling D, Katupitiya L, Ulett GC. Copper Intoxication in Group B Streptococcus Triggers Transcriptional Activation of the cop Operon That Contributes to Enhanced Virulence during Acute Infection. J Bacteriol. 2021;203(19):e0031521. Epub 20210908. doi: 10.1128/JB.00315-21. PubMed PMID: 34251869; PubMed Central PMCID: PMCPMC8447484.

5. Goh KGK, Sullivan MJ, Ulett GC. The Copper Resistome of Group B Streptococcus Reveals Insight into the Genetic Basis of Cellular Survival during Metal Ion Stress. J Bacteriol. 2022;204(5):e0006822. Epub 20220411. doi: 10.1128/jb.00068-22. PubMed PMID: 35404113; PubMed Central PMCID: PMCPMC9112871.

6. Ipe DS, Ben Zakour NL, Sullivan MJ, Beatson SA, Ulett KB, Benjamin WHJ, et al. Discovery and Characterization of Human-Urine Utilization by Asymptomatic-Bacteriuria-Causing *Streptococcus agalactiae*. Infect Immun. 2015;84(1):307-19. doi: 10.1128/IAI.00938-15. PubMed PMID: 26553467; PubMed Central PMCID: PMC4694007.

7. LeBlanc DJ, Lee LN, Abu-Al-Jaibat A. Molecular, genetic, and functional analysis of the basic replicon of pVA380-1, a plasmid of oral streptococcal origin. Plasmid. 1992;28(2):130-45. Epub 1992/09/01. PubMed PMID: 1409970.

8. Maguin E, Prevost H, Ehrlich SD, Gruss A. Efficient insertional mutagenesis in lactococci and other gram-positive bacteria. J Bacteriol. 1996;178(3):931-5. Epub 1996/02/01. doi: 10.1128/jb.178.3.931-935.1996. PubMed PMID: 8550537; PubMed Central PMCID: PMCPMC177749.
