# Supplementary material for: Regulatory cross-talk supports resistance to Zn intoxication in Streptococcus
Source: PLoS Pathog. 2022 Jul 21;18(7):e1010607. doi: 10.1371/journal.ppat.1010607 (PMC9345489; doi:10.1371/journal.ppat.1010607)
Supplement: S3 Table — (DOCX) [file ppat.1010607.s003.docx]

**Supplementary Table S3.** Primers used in this study

| **Oligos** | | |
| --- | --- | --- |
| **ID*** | **Sequence** | **Usage** |
| M13F | GTAAAACGACGGCCAG | Sequencing |
| M13R | CAGGAAACAGCTATGAC | Sequencing |
| pDL278_seq_F1 | GCCTCTTCGCTATTACGCCA | Sequencing |
| pDL278_seq_R1 | CTGGAAAGCGGGCAGTGA | Sequencing |
| stp1_chkF1 | ATGCATGTAGTGCACCAGGA | Sequencing |
| stp1_chkR1 | TTTTCTGCAAGGATTGATGG | Sequencing |
| stk1_chkF1 | TGCTAGCGAGATGACTGTAACTG | Sequencing |
| stk1_chkR1 | AACATTATCCTGCCCAGATACAA | Sequencing |
| *****01646_chkF1 | TGGTGAGGTGAAGGTAGAAGA | Sequencing |
| *****01646_chkR1 | TGCTTGTTCGTCTTTTGCTG | Sequencing |
| *****01047_chkF1 | CCACGTAATTTGGCAAAAGC | Sequencing |
| *****01049_chkR1 | GCCGAAGCAGACCCTAGTAA | Sequencing |
| *****00876_chkF1 | CCAAAAAGAGCTATTGTGAGGAA | Sequencing |
| *****00876_chkR1 | CATTTCAGCCGCACAAATAA | Sequencing |
| *****02173_chkF1 | CCATCACGCCAATTAAAACA | Sequencing |
| *****02173_chkR1 | TGTTACTATCATATCACGCTAAAATCA | Sequencing |
| *****01596_chkF1 | CAACCTGTGTCAACGCAAAA | Sequencing |
| *****01596_chkR1 | TTGATGGAATATTTCCTTTATTACTGA | Sequencing |
| *****00885_chkF1 | TTAAAATCGGTAACAAAAGAGGA | Sequencing |
| *****00885_chkR1 | CAAGGCATGAAGGTAAAGGTG | Sequencing |
| *****00838_chkF1 | TCACGATACGCAAAGTGGAC | Sequencing |
| *****00838_chkR1 | ATCAATTTGCCTTTTGCGATA | Sequencing |
| dnaN-1F | CAACAAGAAAGCCGTCCAAT | qPCR |
| dnaN-1R | TCTGTCGCAACAGCCTTAAA | qPCR |
| czcD-1F | TCAATATCTGGTCAATGGATGG | qPCR |
| czcD-1R | TAATGTTGGCAAATCGTTCG | qPCR |
| copY-1F | CAGAAAGTTTGCCAGACCAA | qPCR |
| copY-1R | GCATTGACACTGACCAGGAA | qPCR |
| copA-1F | CCTTATCGCCAAACGTGATT | qPCR |
| copA-1R | ATTGCTAATTGGTGCCGTTC | qPCR |
| sczA-1F | GGAAGTTACCCGATTGAGCA | qPCR |
| sczA-1R | TGCCAGGAGGAGAATAGGAA | qPCR |
| *****00876_F1 | CGGGCGATTCTAAGTGGATA | qPCR |
| *****00876_R1 | CGGCAAGTTAAACGTGGATT | qPCR |
| *****01359_1F | CCCAAAATGGGAAGAATTACC | qPCR |
| *****01359_1R | TCATCGAAGCGGTGAGTAGTT | qPCR |
| *****00923_1F | GCACAAGGATTGGCAGATTT | qPCR |
| *****00923_1R | GCGGTCCAAAAATCGAACTA | qPCR |
| *****00928_1F | TTGCGCGTGAAGAGAAACTA | qPCR |
| *****00928_1R | CCATTGAGCATGTTCGTGAC | qPCR |
| *****02108_1F | GCGCATAACCATCACTCAGA | qPCR |
| *****02108_1R | TTGGCAGTTTCTAAGGCAGTC | qPCR |
| AdptrPrimer1 | P-GATCGGAAGAGCACACGTCT | TraDIS Adaptor primer 1 |
| AdptrPrimer2 | ACACTCTTTCCCTACACGACGCTCTTCCGATC*T | TraDIS Adaptor primer 2 |
| IndexPr1 | CAAGCAGAAGACGGCATACGAGATCGGTTCGCCTTAACACTCTTTCCCTACACGACGCTCTTCCGATCT | TraDIS Indexing PCR primer 1 |
| IndexPr2 | CAAGCAGAAGACGGCATACGAGATCGGTCTAGTACGACACTCTTTCCCTACACGACGCTCTTCCGATCT | TraDIS Indexing PCR primer 2 |
| IndexPr3 | CAAGCAGAAGACGGCATACGAGATCGGTTTCTGCCTACACTCTTTCCCTACACGACGCTCTTCCGATCT | TraDIS Indexing PCR primer 3 |
| IndexPr4 | CAAGCAGAAGACGGCATACGAGATCGGTGCTCAGGAACACTCTTTCCCTACACGACGCTCTTCCGATCT | TraDIS Indexing PCR primer 4 |
| IndexPr5 | CAAGCAGAAGACGGCATACGAGATCGGTAGGAGTCCACACTCTTTCCCTACACGACGCTCTTCCGATCT | TraDIS Indexing PCR primer 5 |
| IndexPr6 | CAAGCAGAAGACGGCATACGAGATCGGTCATGCCTAACACTCTTTCCCTACACGACGCTCTTCCGATCT | TraDIS Indexing PCR primer 6 |
| IndexPr7 | CAAGCAGAAGACGGCATACGAGATCGGTGTAGAGAGACACTCTTTCCCTACACGACGCTCTTCCGATCT | TraDIS Indexing PCR primer 7 |
| IndexPr8 | CAAGCAGAAGACGGCATACGAGATCGGTCCTCTCTGACACTCTTTCCCTACACGACGCTCTTCCGATCT | TraDIS Indexing PCR primer 8 |
| IndexPr9 | CAAGCAGAAGACGGCATACGAGATCGGTAGCGTAGCACACTCTTTCCCTACACGACGCTCTTCCGATCT | TraDIS Indexing PCR primer 9 |
| Cust-R1-Seq | GTTCATTGATATATCCTCGCTGTCATTTTTATTCATTTTACACTAAAATAGACTTAT | TraDIS Custom read 1 sequencing primer |
| Cust-Ind-Read-Seq | AGATCGGAAGAGCGTCGTGTAGGGAAAGAGTGT | TraDIS Custom index read sequencing primer |

*****numbers relate to ‘Old_locus_tag’ identifiers for *S. agalactiae* 874391 genome sequence, NCBI accession NZ_CP022537.1
