# Supplementary figures and images for: Regulatory cross-talk supports resistance to Zn intoxication in Streptococcus
Source: PLoS Pathog. 2022 Jul 21;18(7):e1010607. doi: 10.1371/journal.ppat.1010607 (PMC9345489; doi:10.1371/journal.ppat.1010607)

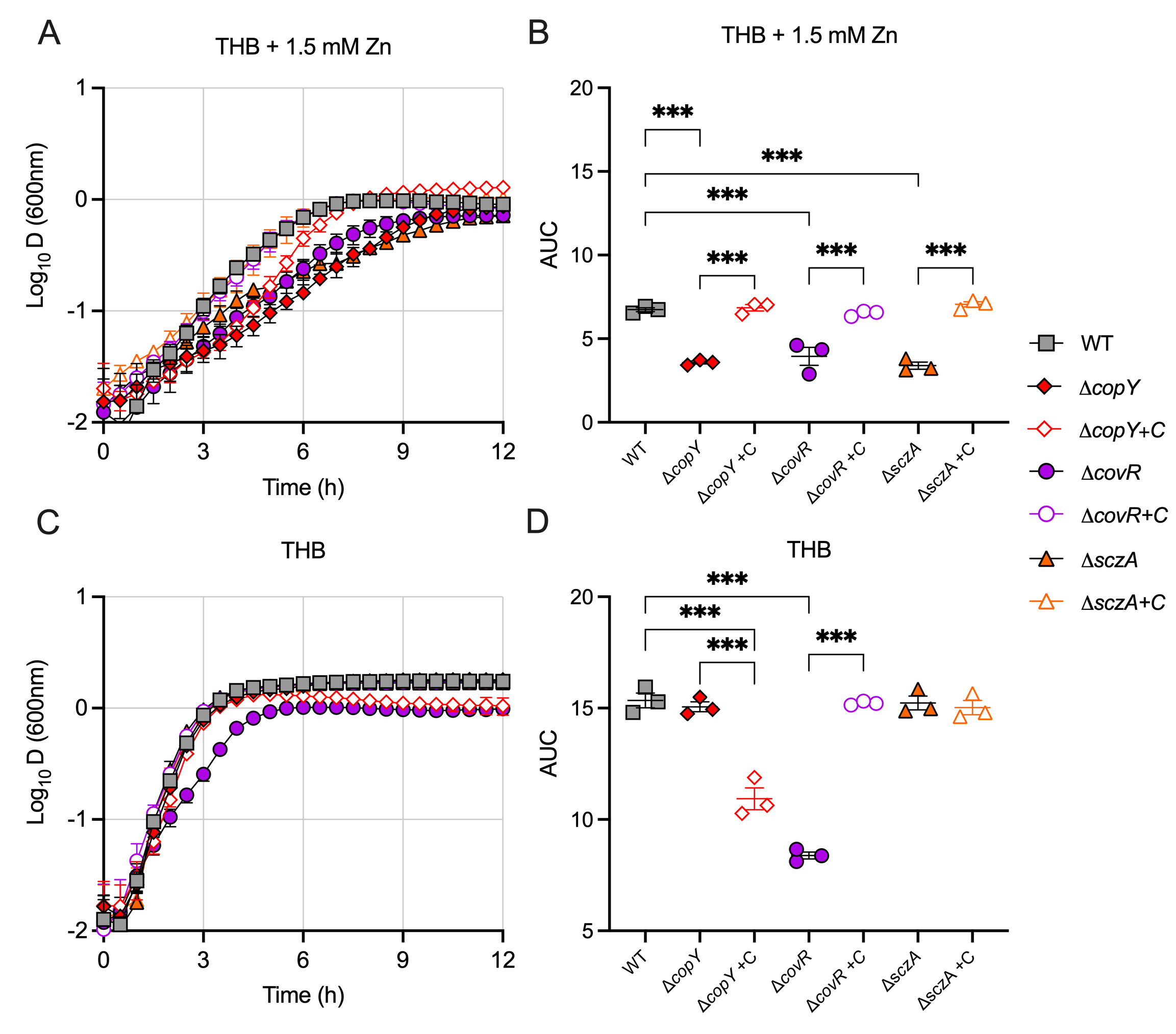

Supplement: S1 Fig — WT, ΔcopY, ΔcovR and ΔsczA mutants and corresponding complemented strains (indicated by +C) were grown in THB medium supplemented with 1.5 mM Zn (A) and compared (B) using Area Under the Curve analysis in followed by ordinary one-way ANOVA and Holm Sidak multiple comparisons (** P < 0.01, *** P < 0.005). Growth curves of the bacteria in control conditions (THB medium alone) are shown for comparison (C) and compared (D) using Area Under the Curve analysis in followed by ordinary one-way ANOVA and Holm Sidak multiple comparisons (** P < 0.01, *** P < 0.005). Measures of Attenuance (D at 600nm) or Area Under the Curve lines shown are mean ± S.E.M (n = 3 biological repeats). (TIFF) [file ppat.1010607.s004.tiff]

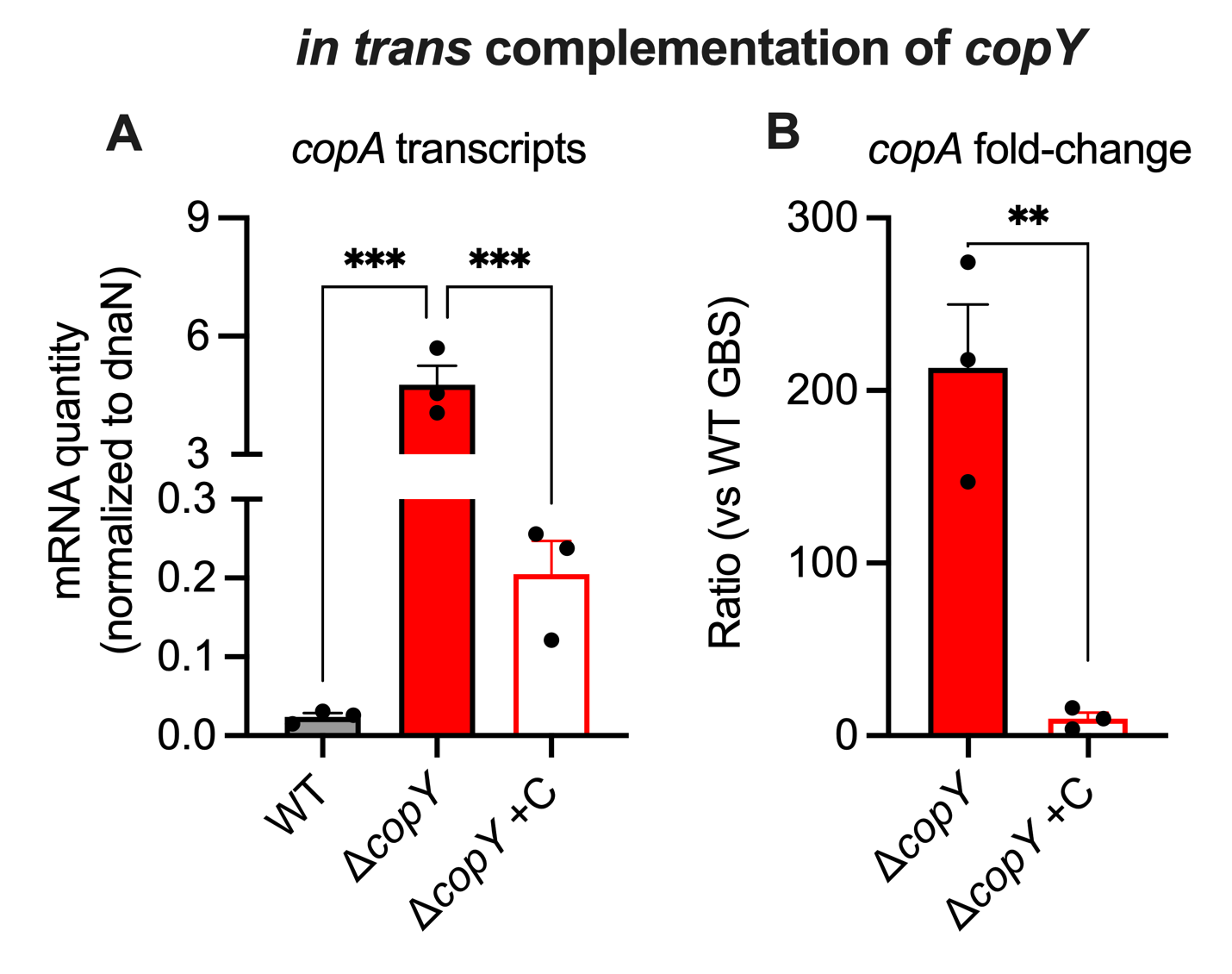

Supplement: S2 Fig — WT, ΔcopY and complemented strains (ΔcopY + C) were grown in THB medium to mid-log phase prior to analysis of copA expression using qRT-PCR. Transcript abundance was normalised using dnaN and relative mRNA quantity (A) and Fold change (B) values compared to WT were calculated using dnaN as housekeeper and ΔΔCT values incorporated primer efficiency values as previously described [82]. Bars show mean ± S.E.M (n = 3 biological repeats). Data were compared using (A) one-way ANOVA with Holm-Sidak multiple comparisons or (B) unpaired t-tests B (** P < 0.01, *** P < 0.005). (TIFF) [file ppat.1010607.s005.tiff]

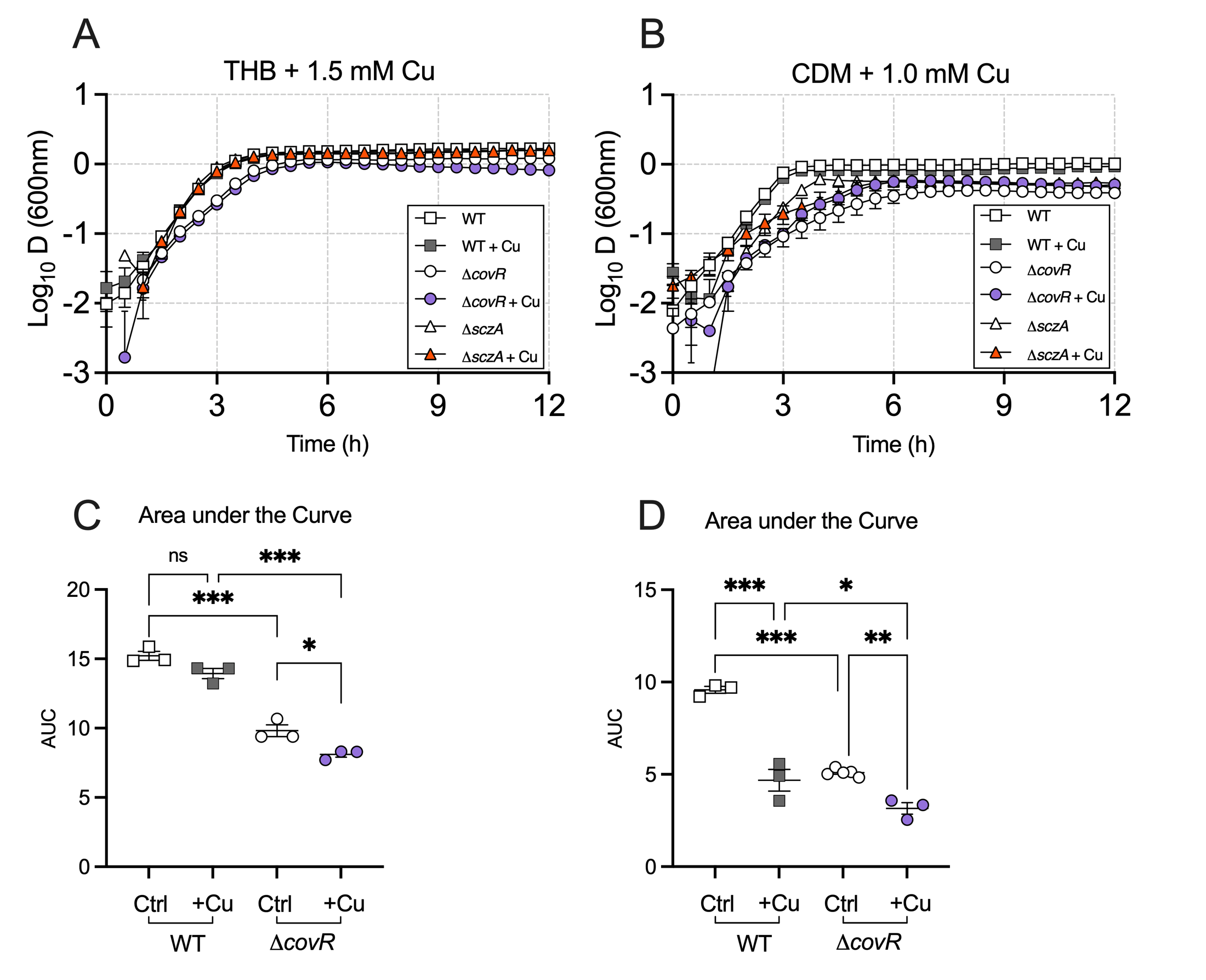

Supplement: S3 Fig — Additional growth analyses of GBS WT (squares), ΔsczA (triangles) and ΔcovR (circles) mutants in rich (THB) or limiting (CDM) medium and subjected to Cu intoxication (filled; 1.5 mM Cu for THB, 1 mM for CDM) vs control conditions (empty; medium alone without supplemental Cu) as indicated. Bars show mean ± S.E.M (n≥3 biological repeats). Data (A, B) were compared using one-way ANOVA with Holm-Sidak multiple comparisons (C,D; * P < 0.05 ** P < 0.01, *** P < 0.005). (TIFF) [file ppat.1010607.s006.tiff]

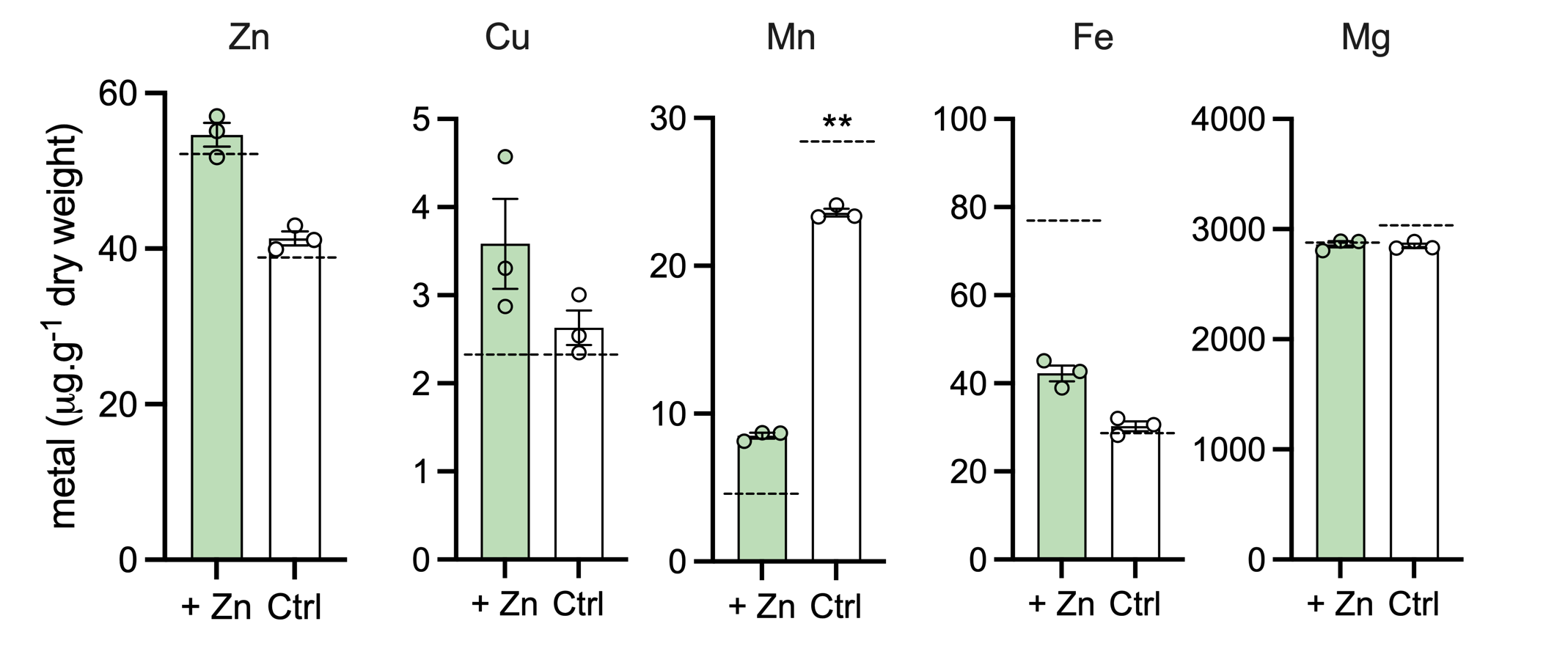

Supplement: S4 Fig — Cells of ΔcovR GBS were exposed to Zn (0.25 mM; green bars) and cellular metal content was compared to unexposed controls (THB only; white bars) using Inductively coupled plasma optical emission spectrometry (ICP-OES). Metal content was normalised using pellet dry weight (μg.g-1 dry weight biomass). Dotted lines indicate mean values from WT GBS from the same condition, published previously [21]. Bars show mean ± S.E.M (n = 3 biological repeats). Means were compared to mean data for WT values in the same conditions using ordinary one-way ANOVA followed by Holm-Sidak multiple comparisons (* P < 0.05). (TIFF) [file ppat.1010607.s007.tiff]

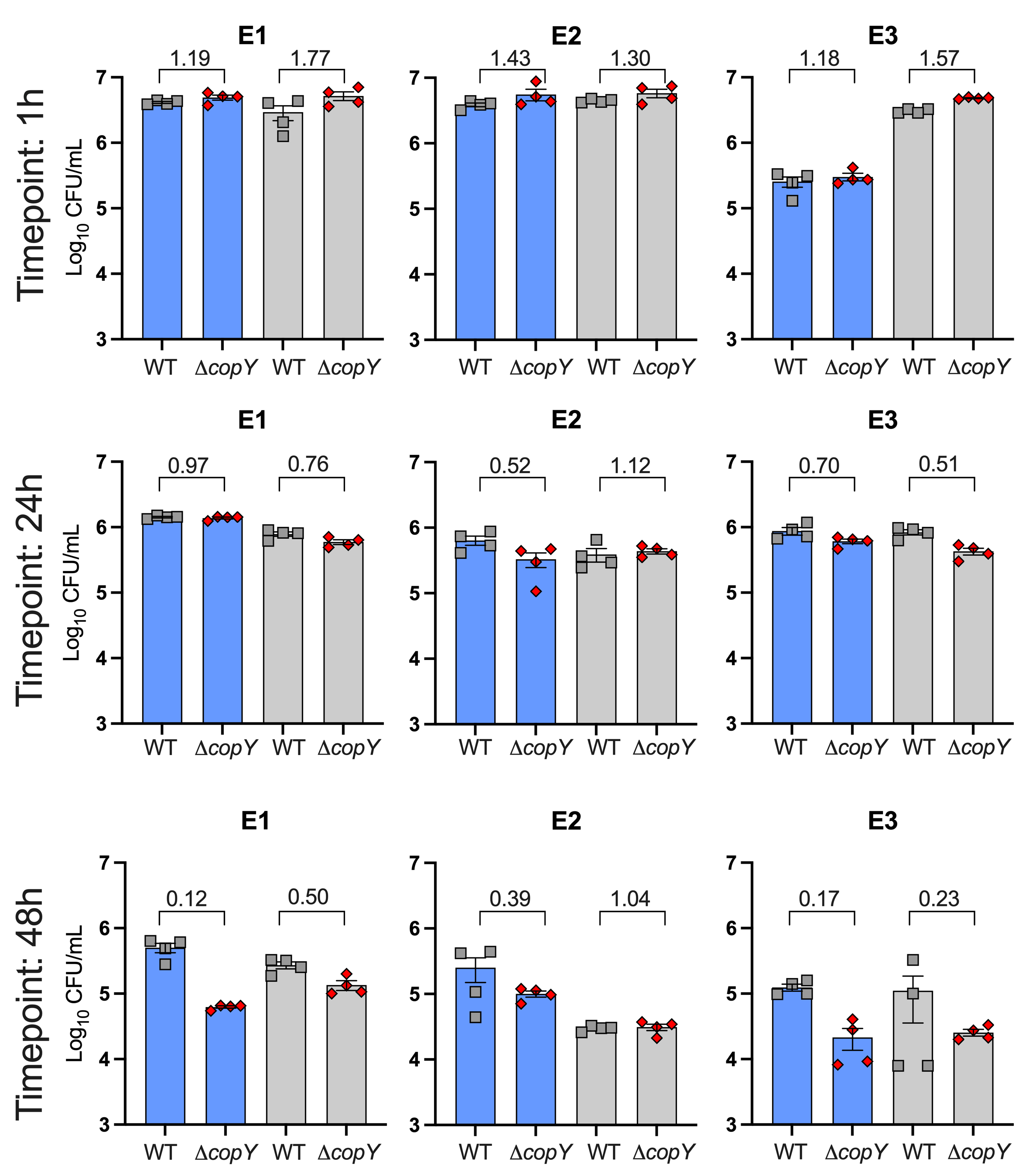

Supplement: S5 Fig — Intramacrophage survival index assays of ΔcopY vs WT shown in Fig 3 comprise three independent experiments (E1-E3), derived from colony forming units per millilitre (CFU/mL) data of WT (grey squares) and ΔcopY GBS (red diamonds) quantified following gentamicin protection assays at 1h, 24h and 48h after addition of antibiotics. Blue shading indicate assays performed using RPMI with 20 μM supplemental Cu, and grey shading indicate RPMI without supplemental Cu. Numbers above brackets indicate the survival index from each strain-strain comparison at each timepoint, for each experiment, relating to the individual datapoints shown in Fig 3. Bars show means ± S.E.M and individual technical replicates (n = 4) are shown for each experiment. (TIFF) [file ppat.1010607.s008.tiff]

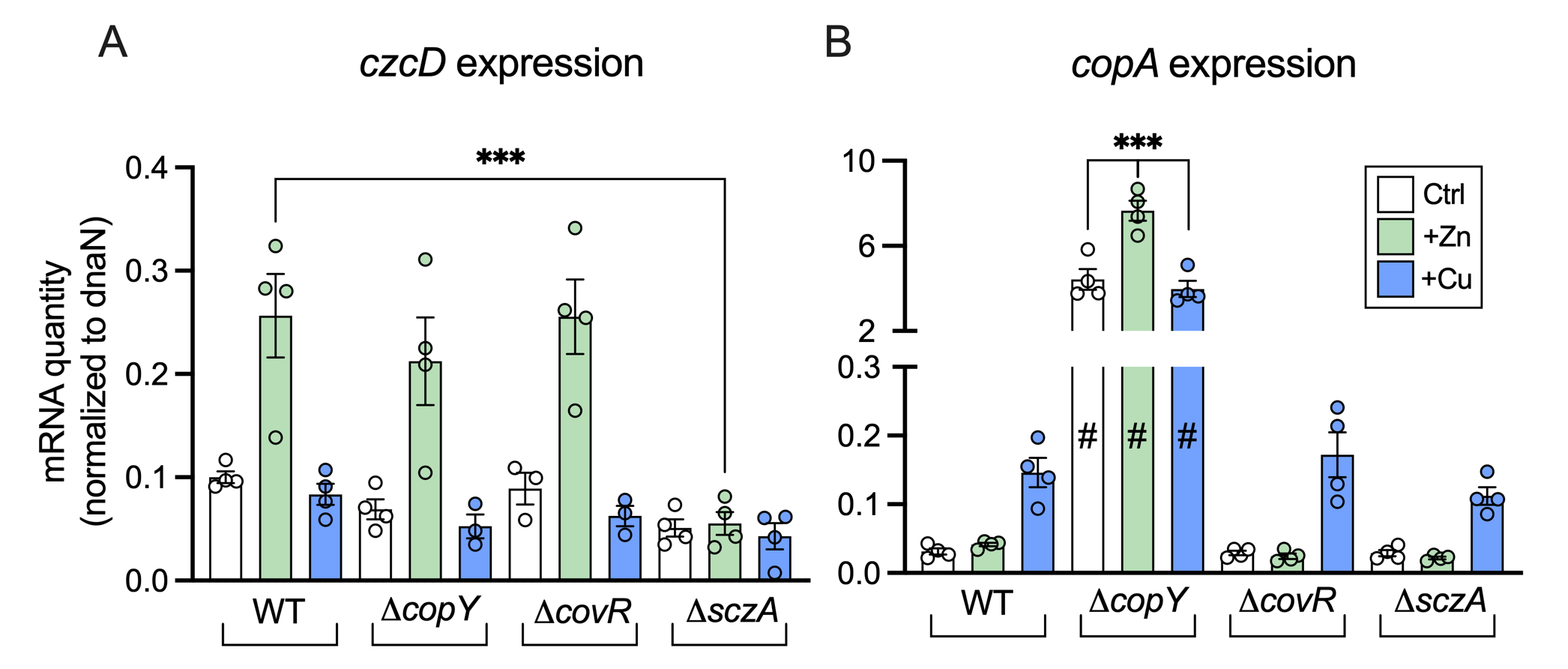

Supplement: S6 Fig — Transcripts of czcD (A) and copA (B) were quantified by qRTPCR from cultures of WT, ΔcopY, ΔcovR and ΔsczA mutants supplemented with Zn (0.25 mM) or Cu (0.5 mM) and compared to non-exposed (THB only) controls (n = 4). Absolute transcript amounts were normalized using housekeeping dnaN and generated from standard curves using GBS genomic DNA. Bars show mean ± S.E.M (n = 3–4 biological repeats). Quantities were compared using ordinary one-way ANOVA and Holm Sidak multiple comparisons (***P < 0.001) noting (#) de-regulation of copA in the ΔcopY strain was significantly different to all other GBS strains and conditions. (TIFF) [file ppat.1010607.s009.tiff]

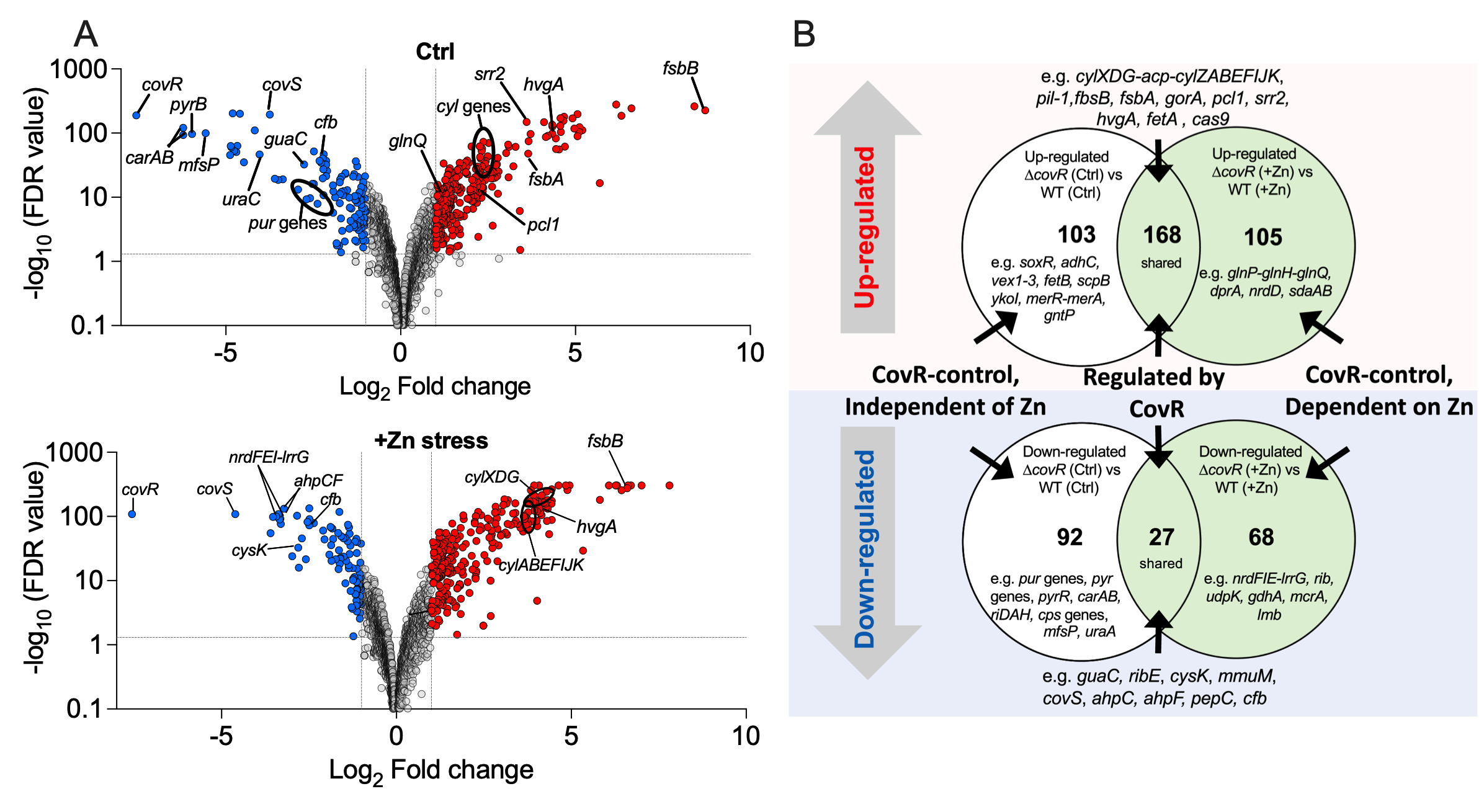

Supplement: S7 Fig — (A) Volcano plots showing data from RNASeq of WT GBS cultures compared to ΔcovR GBS in THB (Ctrl), or THB supplemented with 0.25 mM Zn (+Zn stress). Transcripts up- or down-regulated in response to Zn (n = 4, >± 2-fold, FDR <0.05) are highlighted in red and blue, respectively. Dotted lines show False discovery rate (FDR; q-value) and fold change cut-offs (± 2-fold). Grey points indicate genes that were unchanged, selected genes are identified individually with black lines. FDR values (y-axes) were displayed by -log10 (Y) transformation followed by plotting on a log10 y-axis to generate volcano plots. (B) Venn comparison showing the number of up-regulated and down-regulated genes shared (Regulated by CovR) or unique to control (CovR-control independent of Zn) or +Zn stress conditions (CovR-control dependent on Zn). Selected genes are listed and the complete list is available as S4 Data. (TIFF) [file ppat.1010607.s010.tiff]

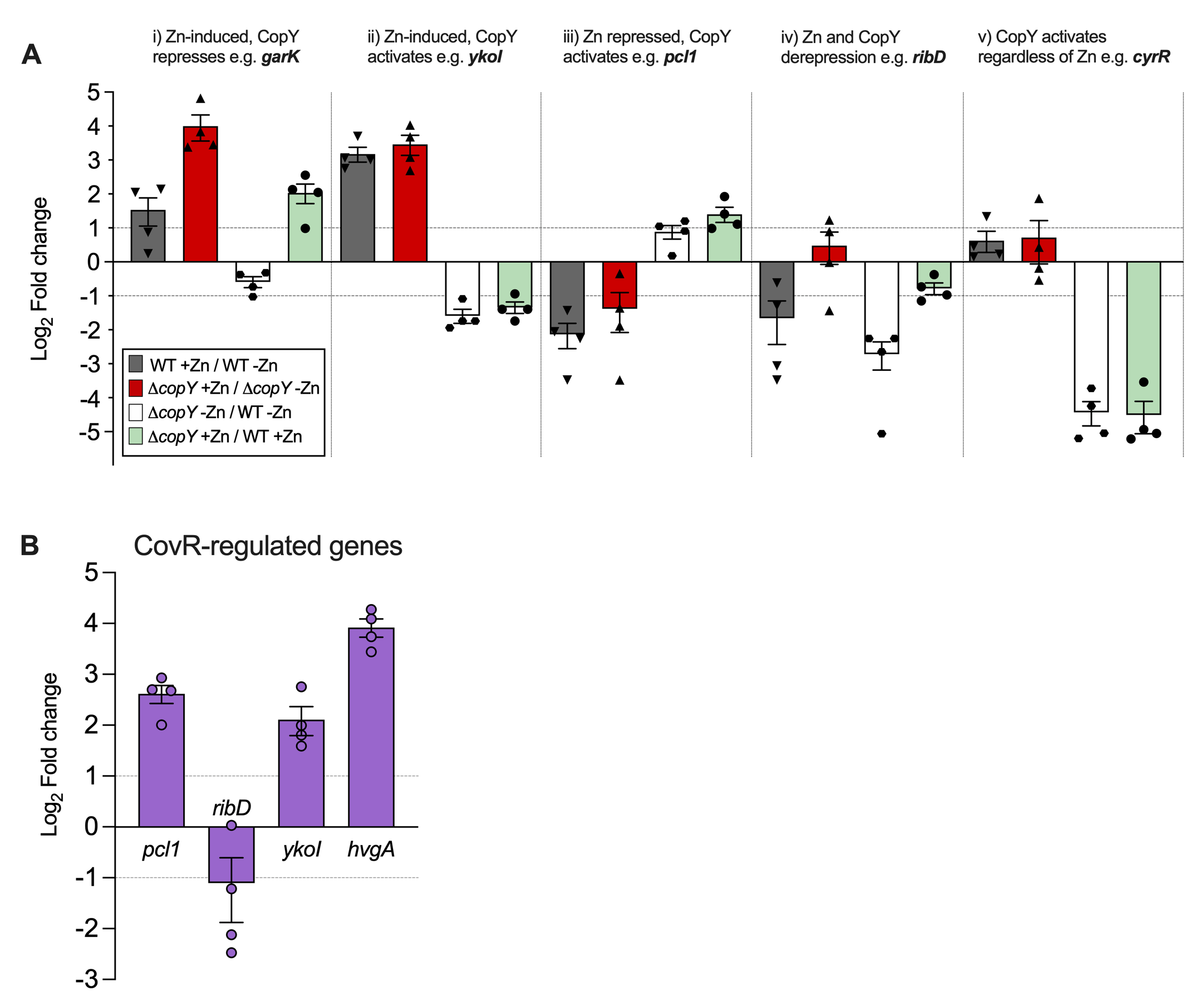

Supplement: S8 Fig — (A) Expression ratios (log2FC) of selected genes, identified by RNAseq as linked to CopY and Zn stress, were compared using qRTPCR as indicated, using RNA isolated from WT or copY- GBS grown in THB (-Zn) or THB supplemented with 0.25 mM Zn (+Zn). Five distinct activation states were apparent based on Zn and/or CopY dependency. Fold change values were calculated using dnaN as housekeeper and ΔΔCT values incorporated primer efficiency values. (B) Transcripts of pcl1, ribD, ykoI and hvgA were quantified from WT and ΔcovR GBS grown in THB by qRT-PCR. Fold change values (ratio of covR- / WT) were calculated using dnaN as housekeeper and ΔΔCT values incorporated primer efficiency values as previously described [82]. Bars show mean ± S.E.M (n = 4 biological repeats). Dotted horizontal lines in A and B indicate ±2 fold change. (TIFF) [file ppat.1010607.s011.tiff]

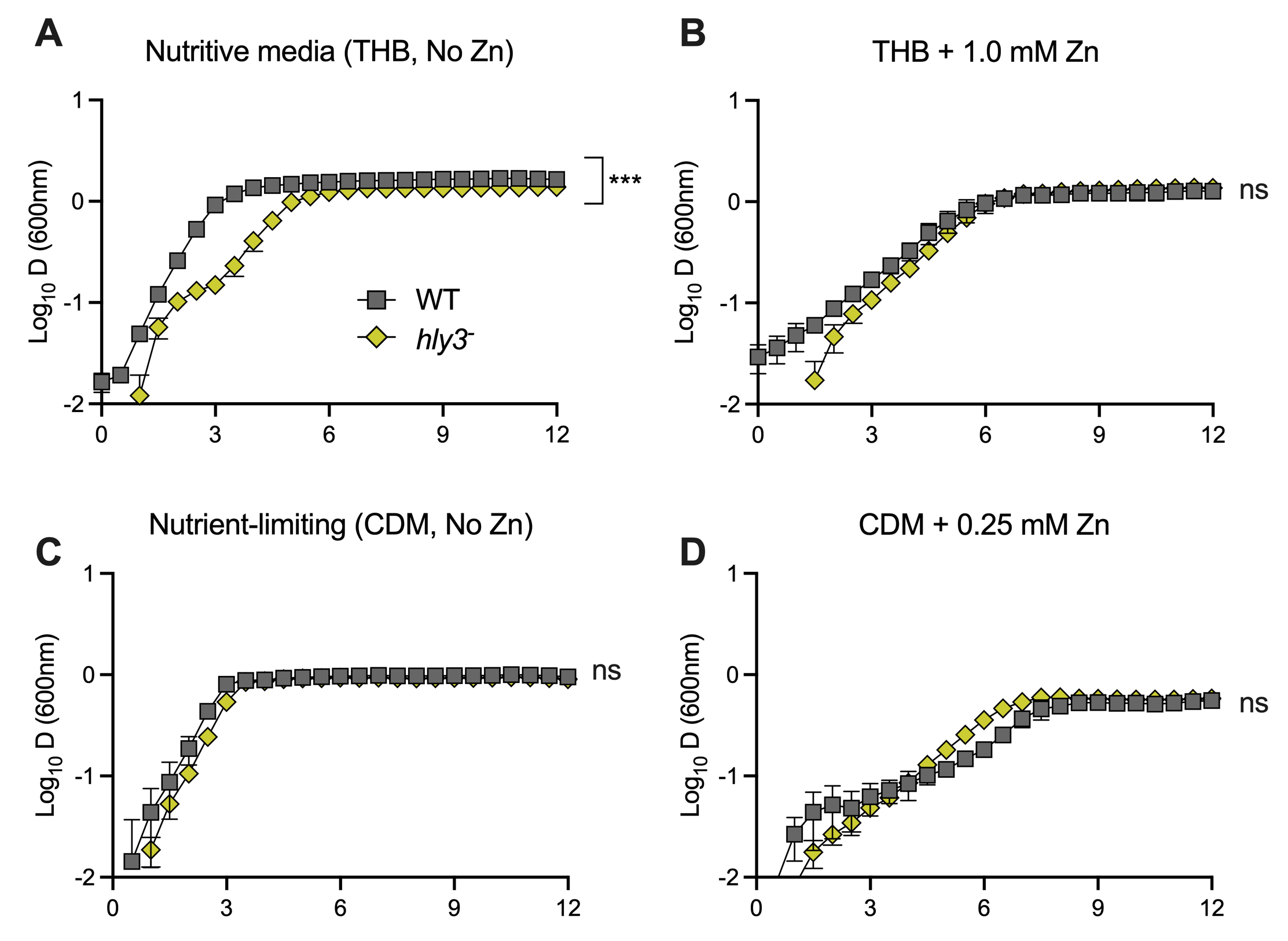

Supplement: S9 Fig — WT and Δhly3 GBS strains were grown in THB without Zn (A) or THB supplemented with 1.0 mM Zn (B), or (C) CDM without Zn, or (D) CDM supplemented with 0.25 mM Zn. Bars show mean ± S.E.M (n = 3 biological repeats) measures of attenuance (D at 600nm). Strains were compared by Area Under the Curve analysis followed by unpaired t-tests for each condition (*** P<0.001). (TIFF) [file ppat.1010607.s012.tiff]

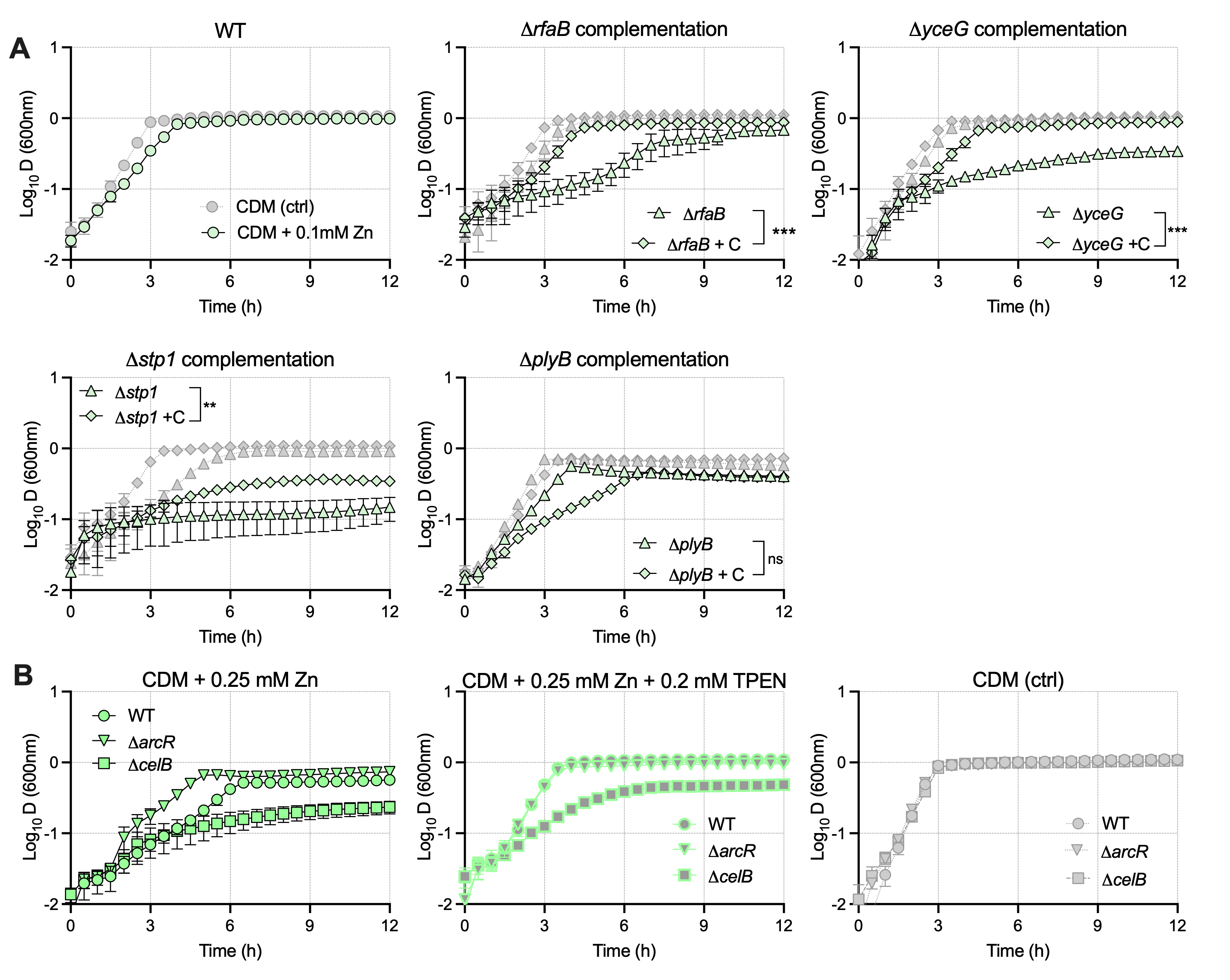

Supplement: S10 Fig — WT and isogenic mutants from TraDIS assays were grown in CDM (ctrl; grey) or CDM +0.1 mM Zn as indicated (A) and compared to growth of complemented strains for each mutant. Bars show mean ± S.E.M (n = 3 biological repeats) measures of attenuance (D at 600nm). Strains were compared by Area Under the Curve analysis followed by unpaired t-tests for each condition (** P < 0.01, *** P<0.001). WT, and the ΔarcA and ΔcelB mutants, for which complementation plasmids are not available (B), were grown in high Zn stress (CDM +0.25 mM Zn), high Zn stress supplemented with TPEN (CDM +0.25 mM Zn + 0.2 mM TPEN) or control conditions (CDM). Bars show mean ± S.E.M (n = 3 biological repeats) measures of attenuance (D at 600nm). (TIFF) [file ppat.1010607.s013.tiff]

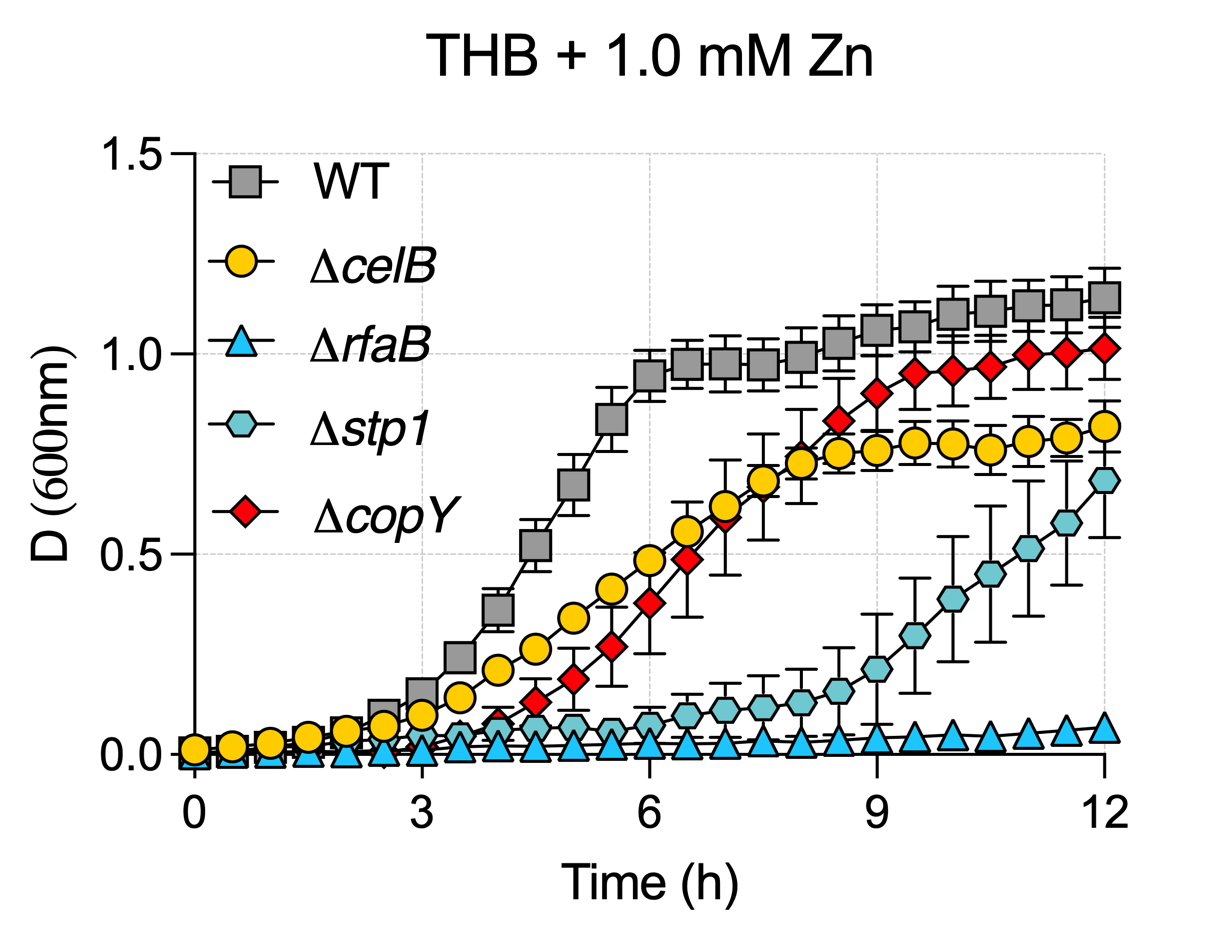

Supplement: S11 Fig — WT GBS and ΔcopY, ΔcelB, ΔrfaB and Δstp1 mutants were grown in THB supplemented with 1.0 mM Zn for 12h, to monitor growth in conditions identical to those used for TraDIS analysis. Bars show mean ± S.E.M (n = 3 biological repeats) measures of attenuance (D at 600nm). (TIFF) [file ppat.1010607.s014.tiff]
